# Supplementary material for: Genetic association meta-analysis is susceptible to confounding by between-study cryptic relatedness
Source: HGG Adv. 2026 Jul 27;7(4):100657. doi: 10.1016/j.xhgg.2026.100657 (PMC13487679; doi:10.1016/j.xhgg.2026.100657)
Supplement: Supplementary file 1 [file mmc1.pdf]

HGGA, Volume *7*

Supplemental information

Genetic association meta-analysis is susceptible to confounding by between-study cryptic relatedness

Tiffany Tu and Alejandro Ochoa

Supplemental Figures

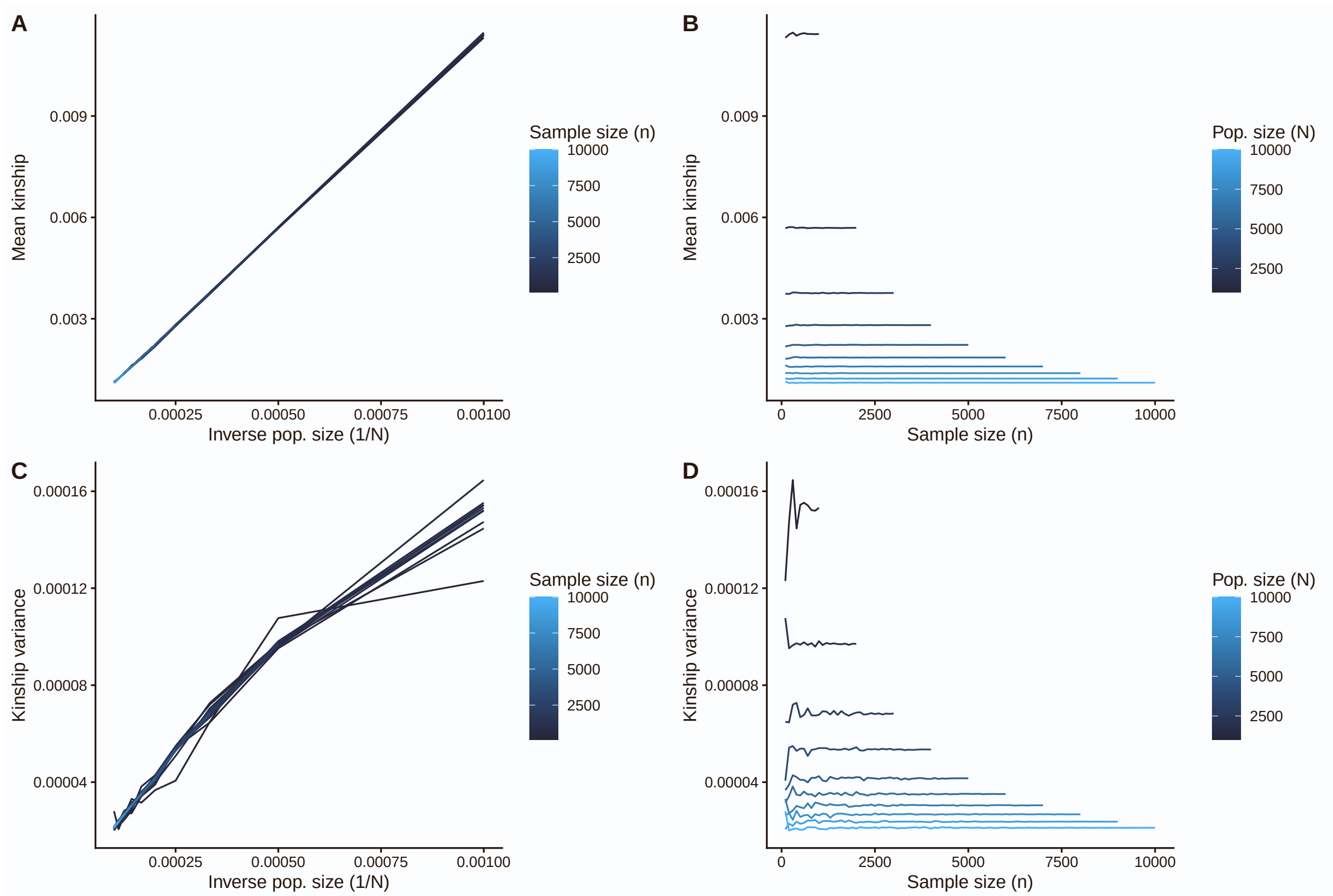

Fig. S1: **Mean and variance of kinship values between studies depend on population size N but not sample size n.** We drew random large population pedigrees, random subsets of them to form samples, and calculated kinship mean and variance between two random partitions of the sample. Sample estimates plotted are averaged over 10 replicates. **A.** Mean kinship is very well predicted by the inverse of the population size (sample size slices are shown in color, they all overlap). Our best model $\bar{\varphi} = 11.29/N$ had a practically perfect fit ($R^2 = 0.9999$). **B.** Mean kinship does not depend on sample size given the population size (note every population size slice is approximately constant). **C.** Kinship variance is again well predicted by the inverse of the population size, with more noise at the smaller sample size slices. Our best model $\sigma^2_\varphi \approx 0.1125/N + 0.001419/\sqrt{N}$ was not visually as good fit ($R^2 = 0.9958$ on the curve averaged over sample sizes weighted by sample size) compared to our model for the mean. **D.** Kinship variance also does not depend on sample size given the population size, but are again noisier for smaller n.

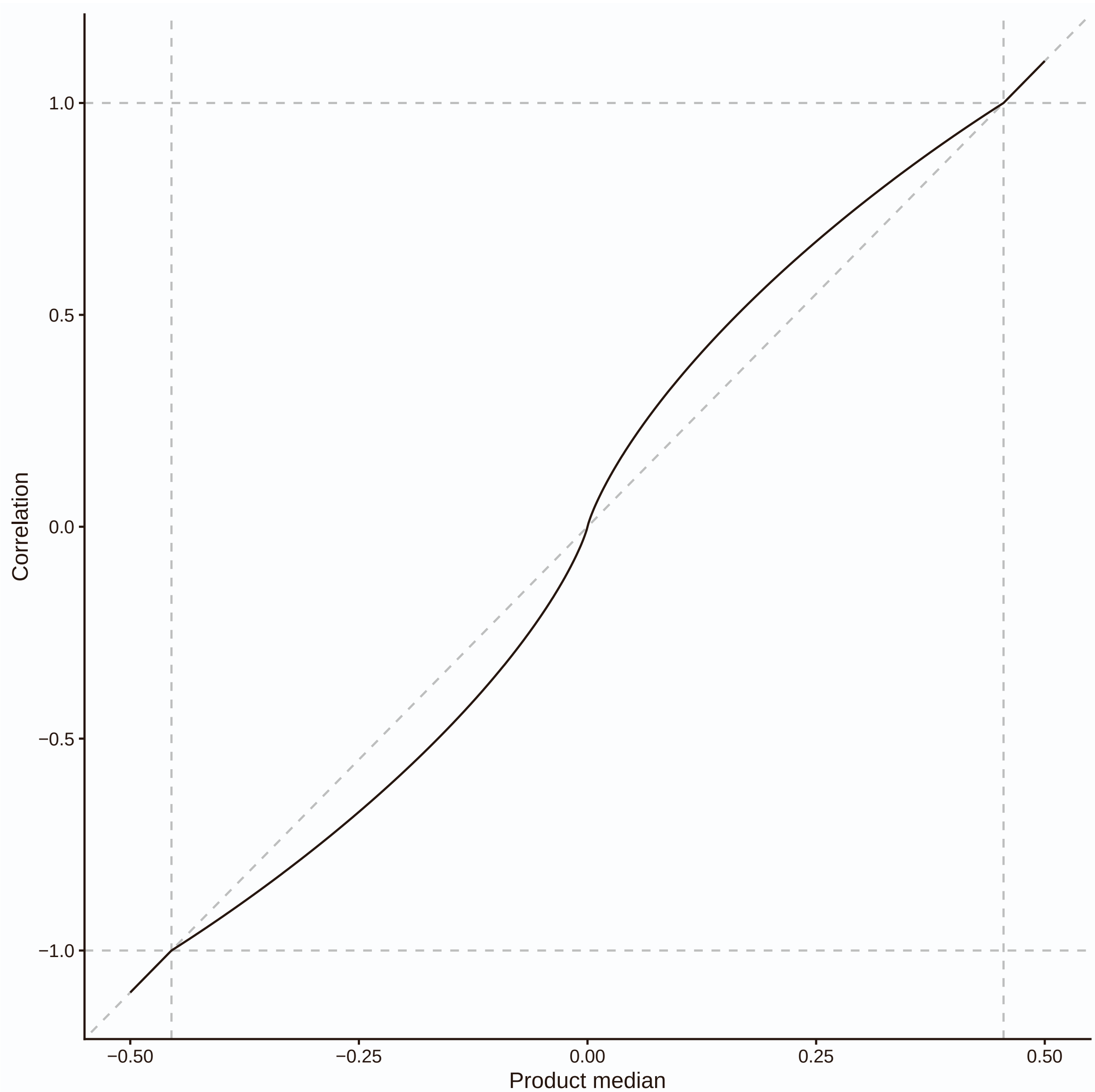

Fig. S2: **Function mapping correlated z-score product medians to correlation parameters.** The vertical gray lines mark $x = -q$ and $x = q$, where $q \approx 0.455$ is the median χ^2_1. The horizontal gray lines mark $y = -1$ and $y = 1$. The diagonal line corresponds to $y = x/q$, which is the standard inflation factor when the two z-scores in the product are equal. The curve and the diagonal line cross at the origin. Thus, for $0 < x < q$, the naive estimate x/q underestimates the true correlation ρ.

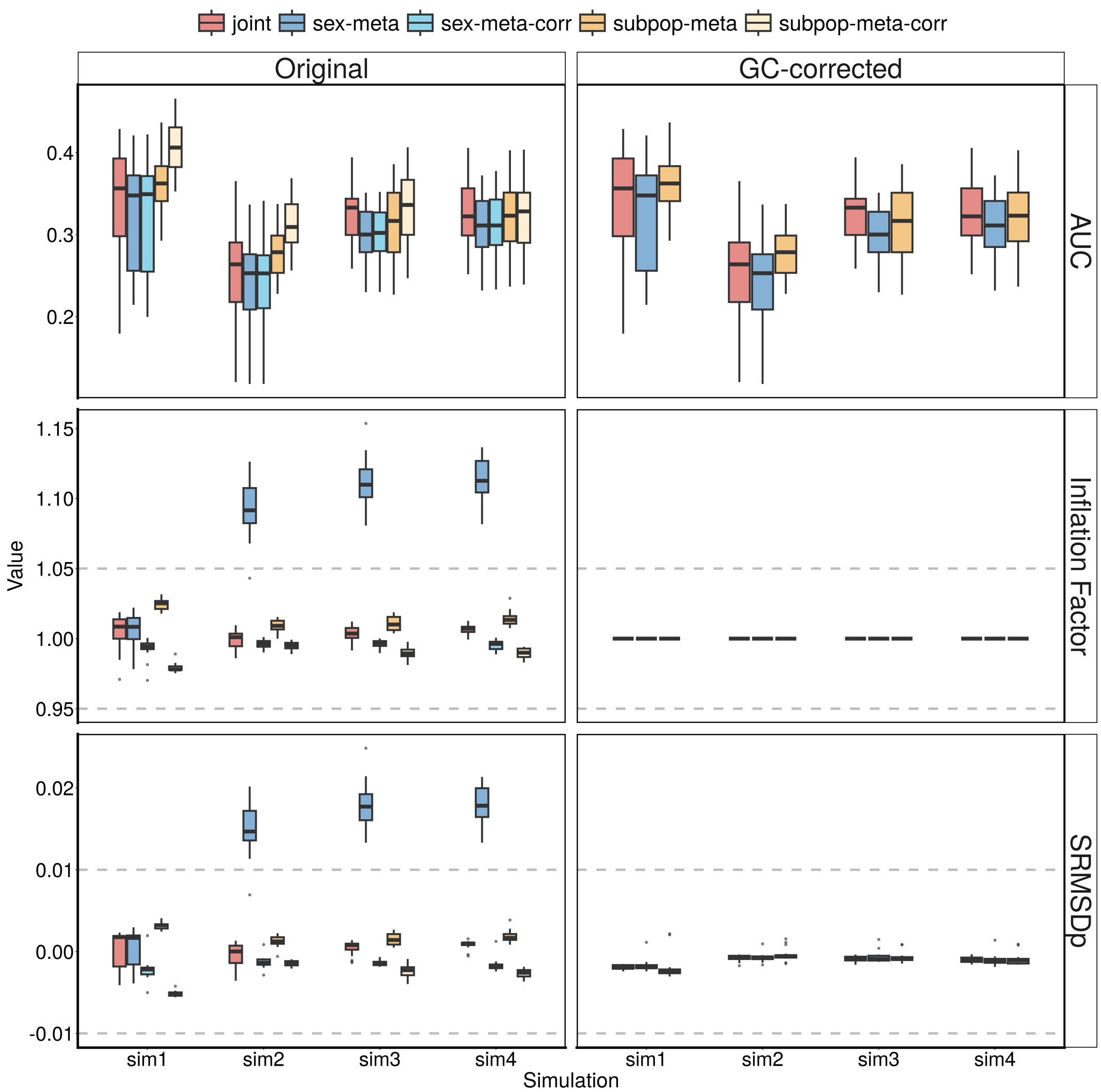

Fig. S3: **Simulation results for binary traits.** Results from 20 replicates of binary traits exhibit similar patterns as for quantitative traits (Figure 4) across all four simulation scenarios.

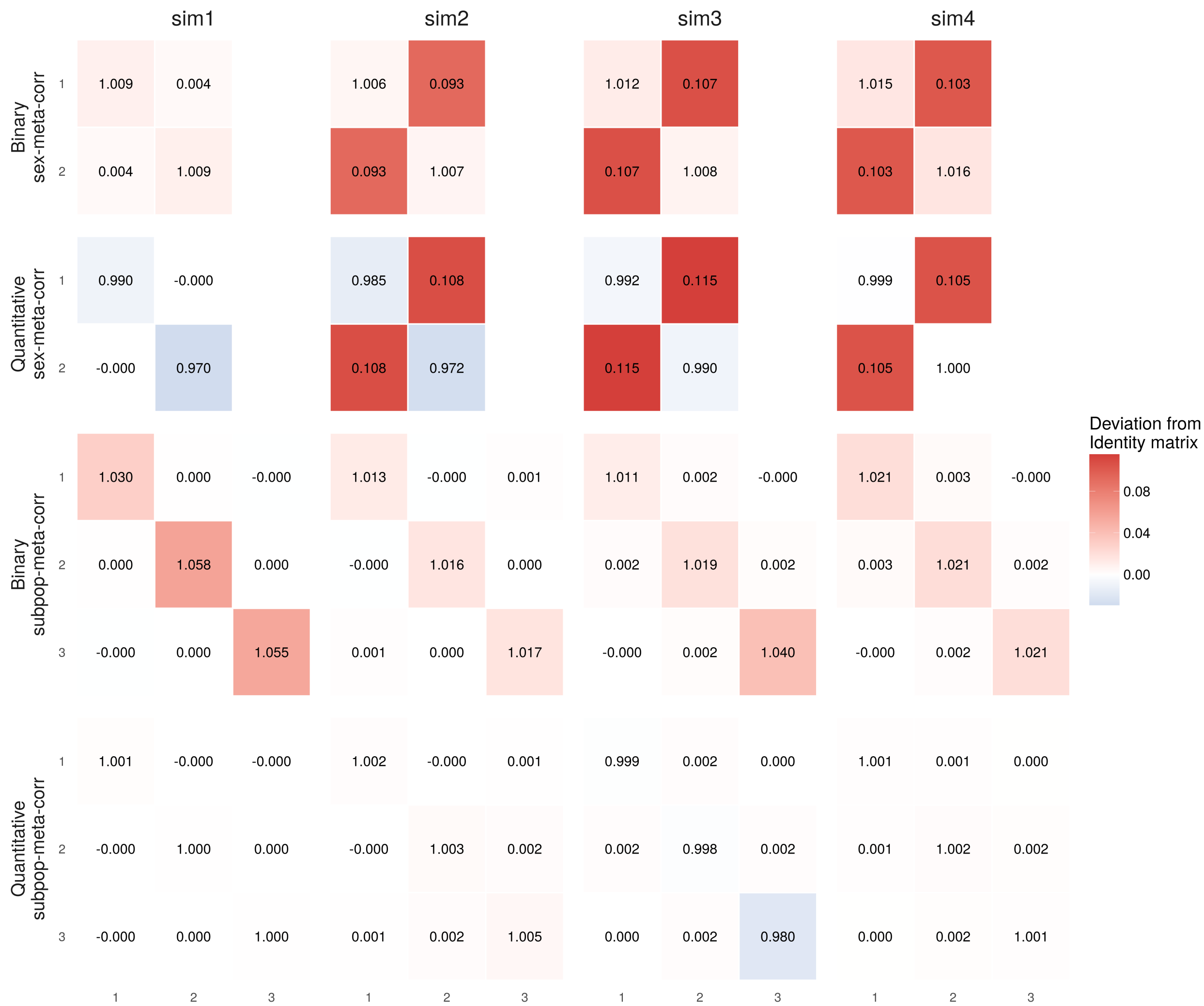

Fig. S4: **Estimated correlation matrices R from meta-corr.** Each cell displays in the number the raw $\mathbf{R}$ value, and in color the value of $\mathbf{R} - \mathbf{I}$, with white corresponds to no deviation from the standard meta-analysis assumption of $\mathbf{R} = \mathbf{I}$ (cases where studies are independent for off-diagonal values, calibrated for diagonal). Deviations reveal where our method provides a correction. All values are averages over the 20 replicates. Correlation matrices are estimated using the median approach.

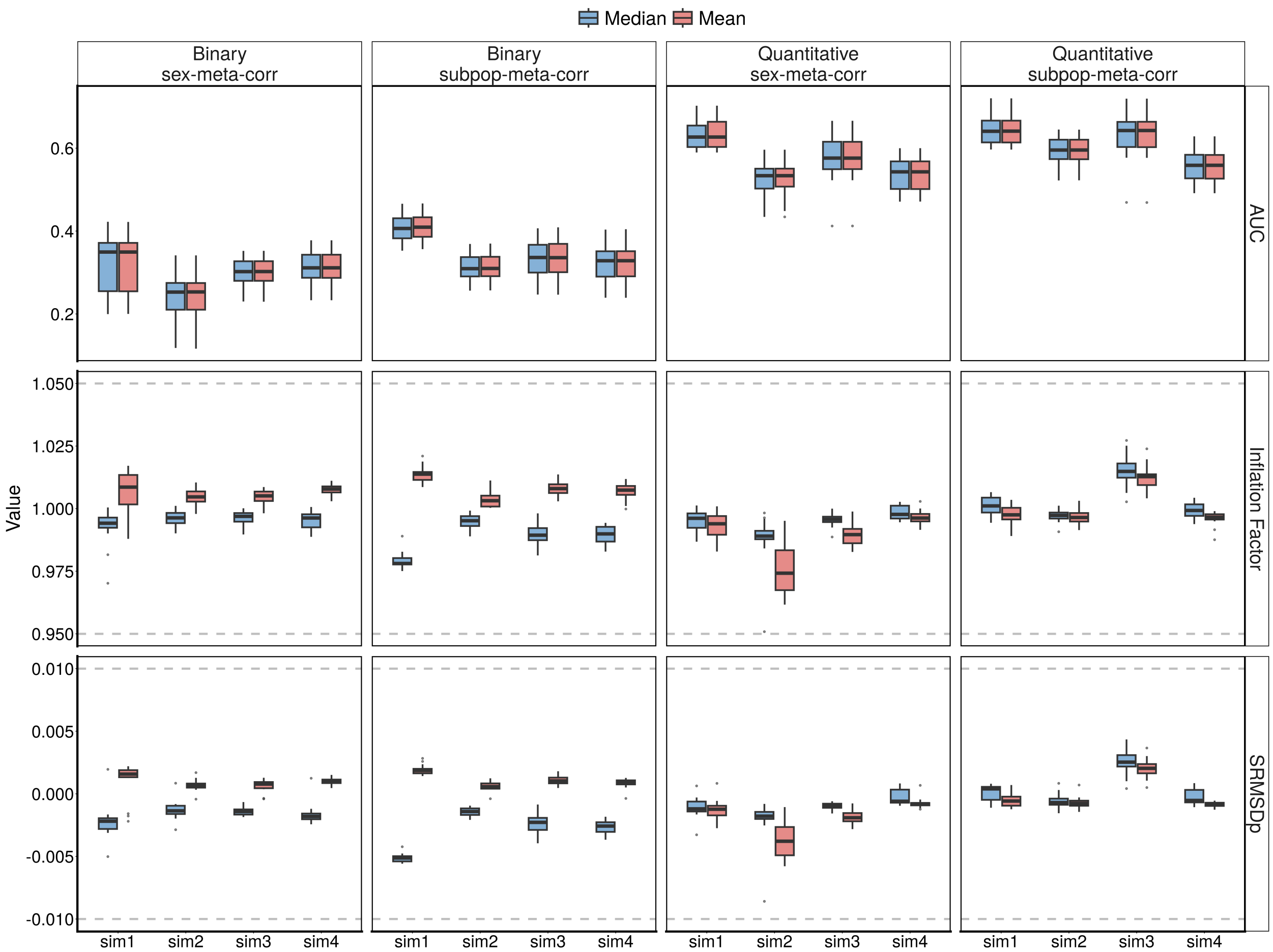

Fig. S5: **Median-based estimation of the covariance matrix performs marginally better than the mean-based approach with 100 causal variants.** We compare median and mean estimators of the covariance in our proposed meta-analysis method for both sex-stratified and subpopulation-stratified meta-analyses. AUC_{PR}, $SRMSD_p$ and inflation are comparable between the two estimators across trait types. Note that all values are well within the thresholds for calibration. For binary traits, however, the mean-based approach produced relatively higher inflation factors.

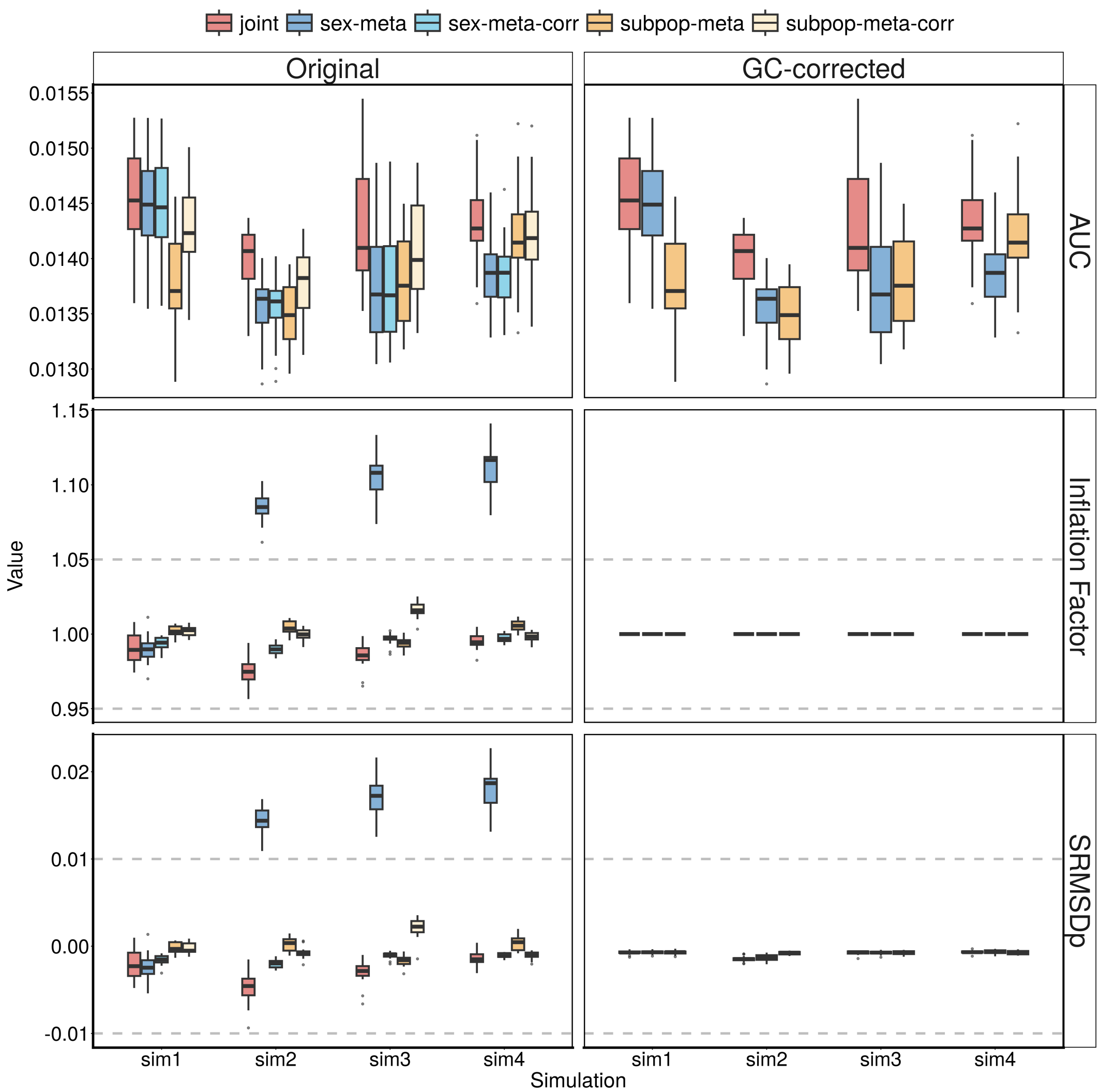

Fig. S6: **Simulation results for quantitative traits with 5,000 causal variants.** Across all four simulations, results from 20 replicates of 5,000 causal variants exhibit similar pattern as the main quantitative analysis (with 100 causal variants; Figure 4). Only trait is re-simulated, keeping genotypes, allele frequencies, and population structure unchanged. AUC_{PR} values are substantially lower partly due to the absense of LD in our simulation framework.

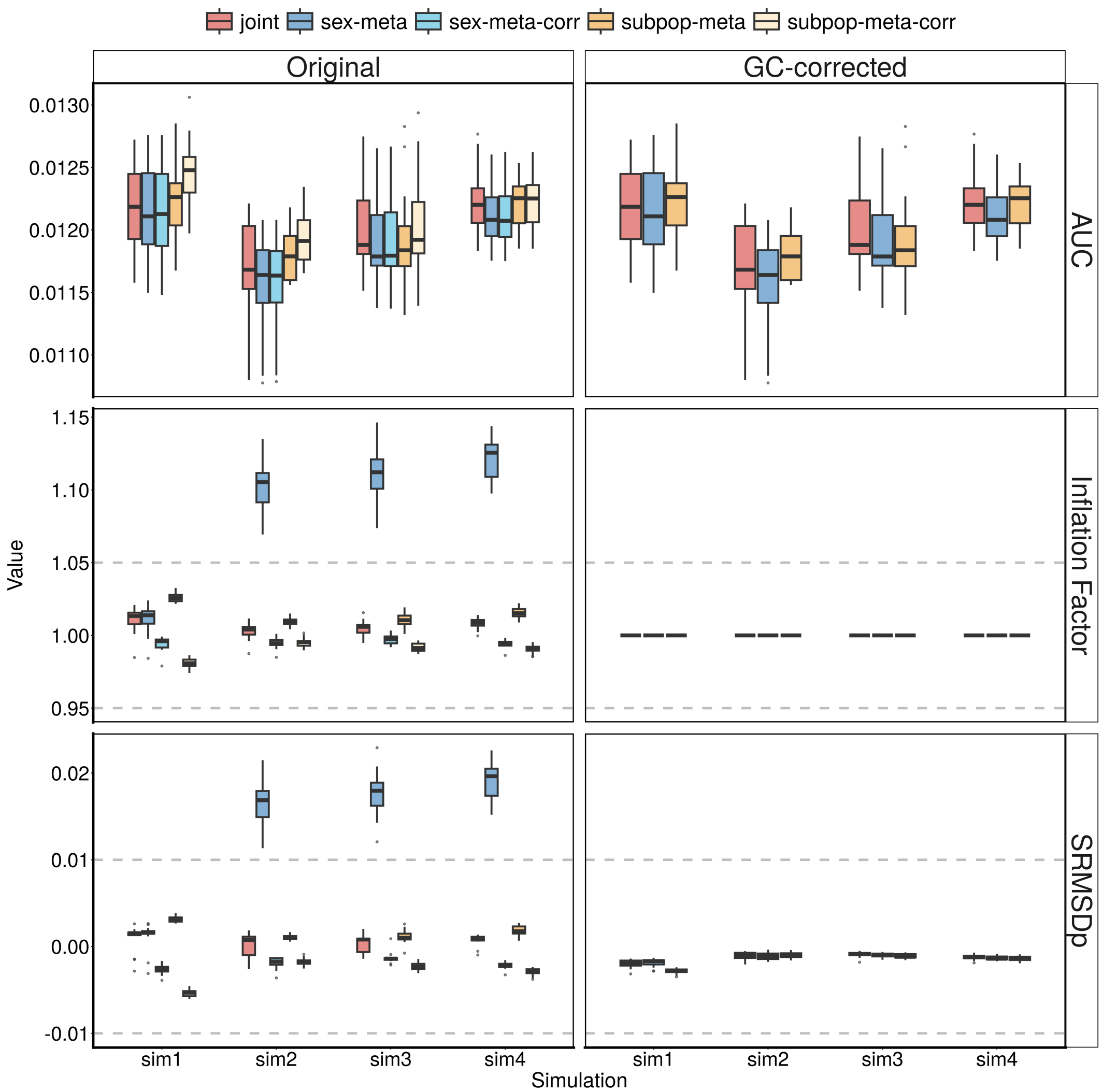

Fig. S7: **Simulation results for binary traits with 5,000 causal variants.** Across all four simulations, results from 20 replicates of 5,000 causal variants exhibit similar pattern as the main binary analysis (with 100 causal variants; Figure S3). Similar to Figure S6, AUC_{PR} values are substantially lower than in the main analysis.

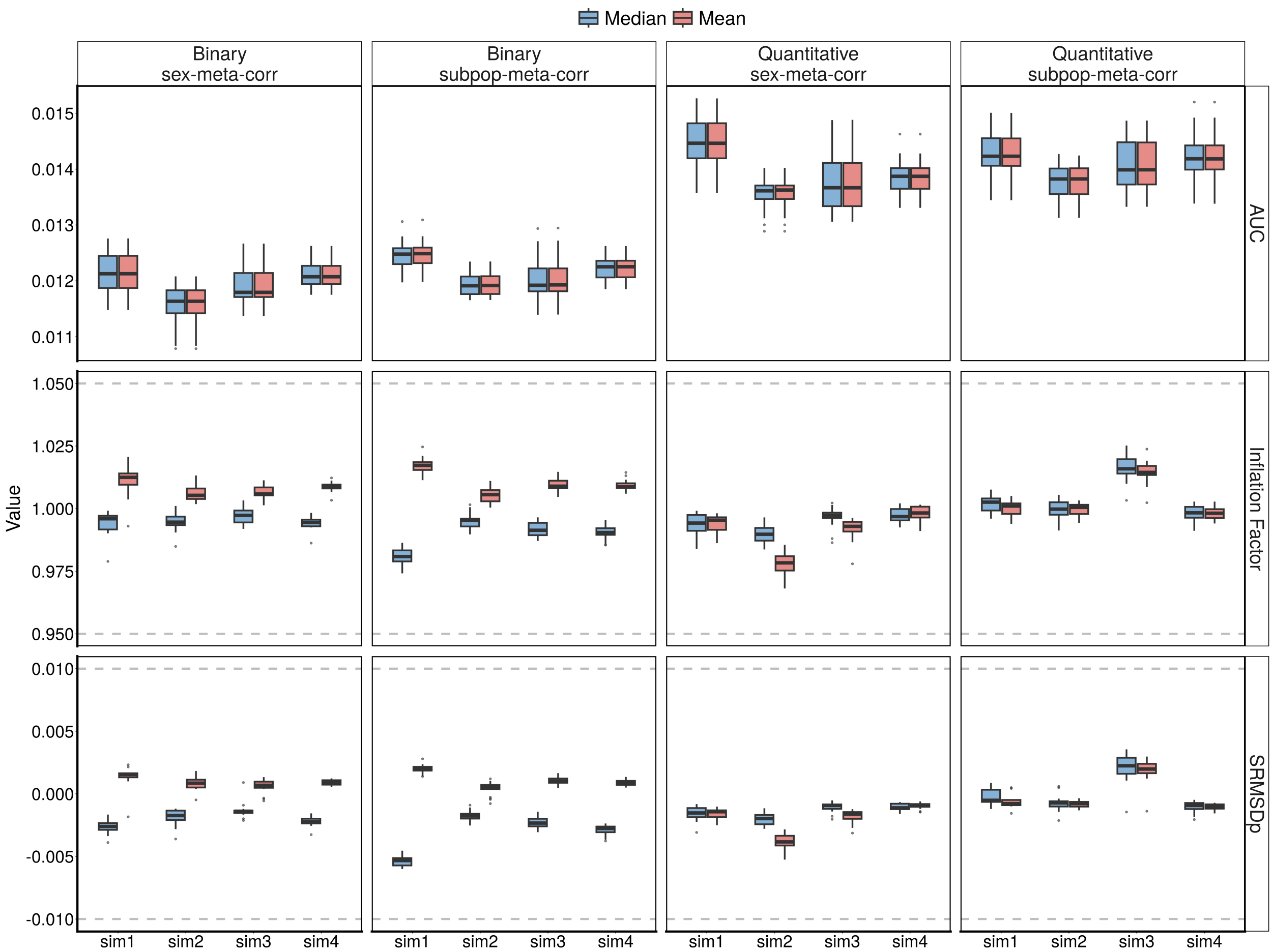

Fig. S8: **Under a more polygenic model of 5,000 causal variants, median-based covariance estimation maintains its marginal advantage over mean-based approach.** We repeat the median and mean estimator comparisons from Figure S5 after re-simulating traits with 50 times more causal SNPs. Inflation factors and SRMSD_p remain well calibrated within thresholds for both estimators, and the mean-based approach again shows higher inflation factors for binary traits. $\mathrm{AUC}_{\mathrm{PR}}$ values collapse to a much narrower range.

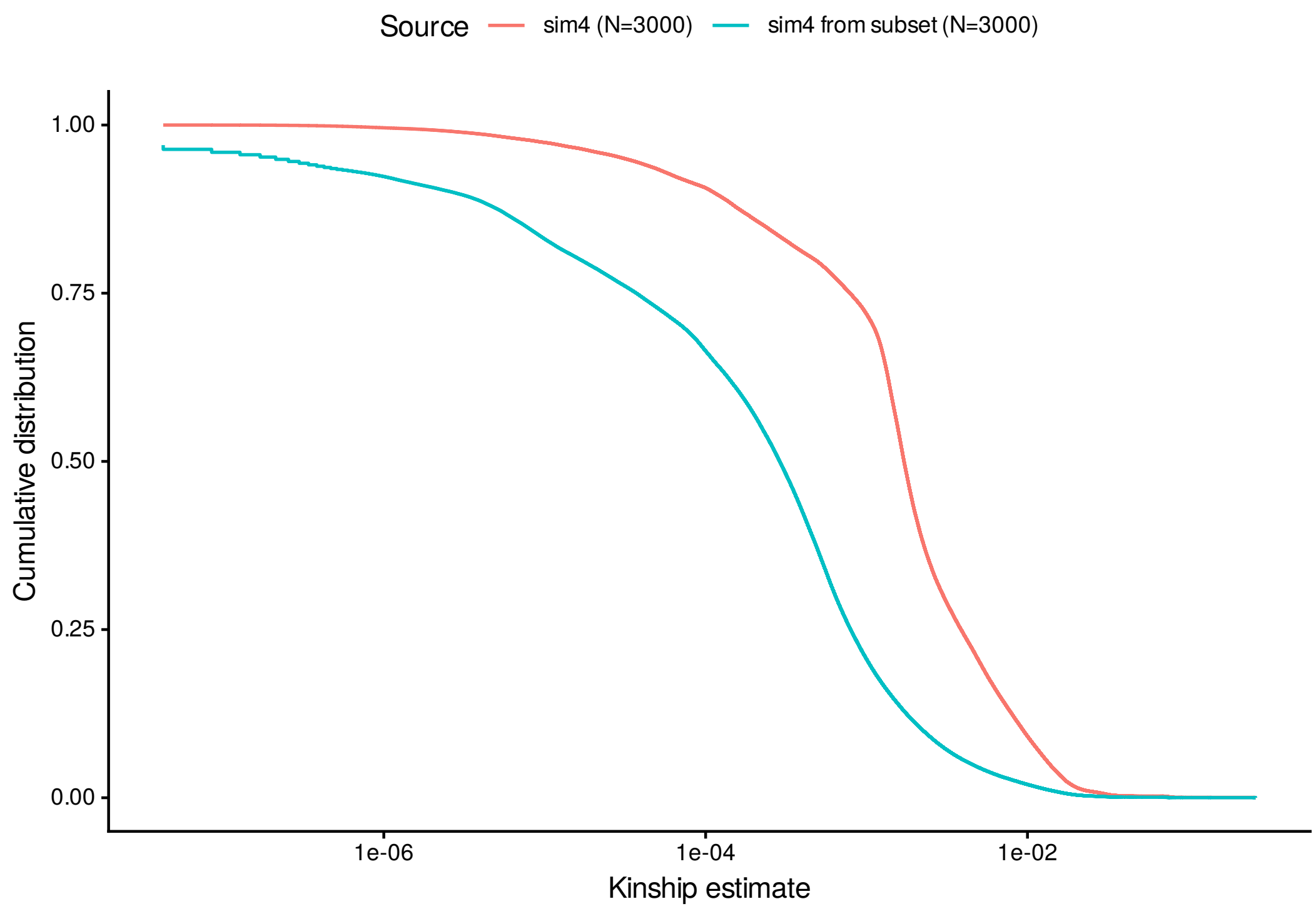

Fig. S9: **Cumulative distribution of pairwise kinship estimates at $n = 3,000$ under different starting population sizes.** True kinship values from each simulation were used. Directly simulating 3,000 individuals over 30 generations (sim4) gives us higher pairwise kinship overall, whereas simulating $n = 10,000$ first and then subsetting to $n = 3,000$ produces proportionally fewer large kinship values (for example, many fewer sibling and first cousin pairs). A typical fourth-degree relatedness cutoff value is 2.2e-02. One replicate from each simulation is plotted. Note x-axis is on a log-scale.

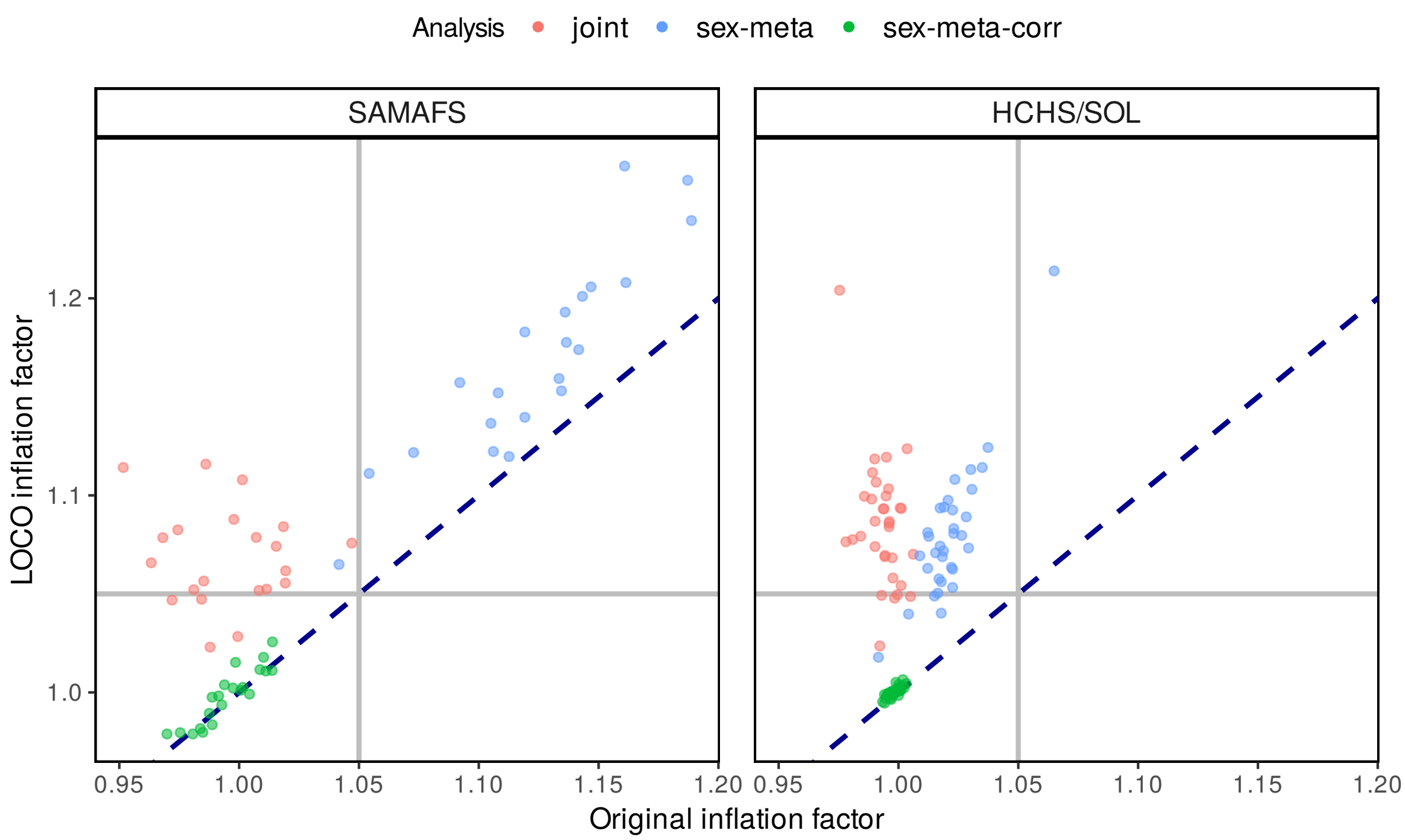

Fig. S10: **LOCO increases inflation factors, but corrected sex-stratified meta-analysis recovers well-calibrated test statistics.** Every trait in both SAMAFS and HCHS/SOL shows greater genomic inflation under LOCO compared to the original analysis for joint and sex-meta (all points above the $y = x$ diagonal). This inflation is successfully eliminated in sex-meta-corr, which clusters near $\lambda = 1.0$.

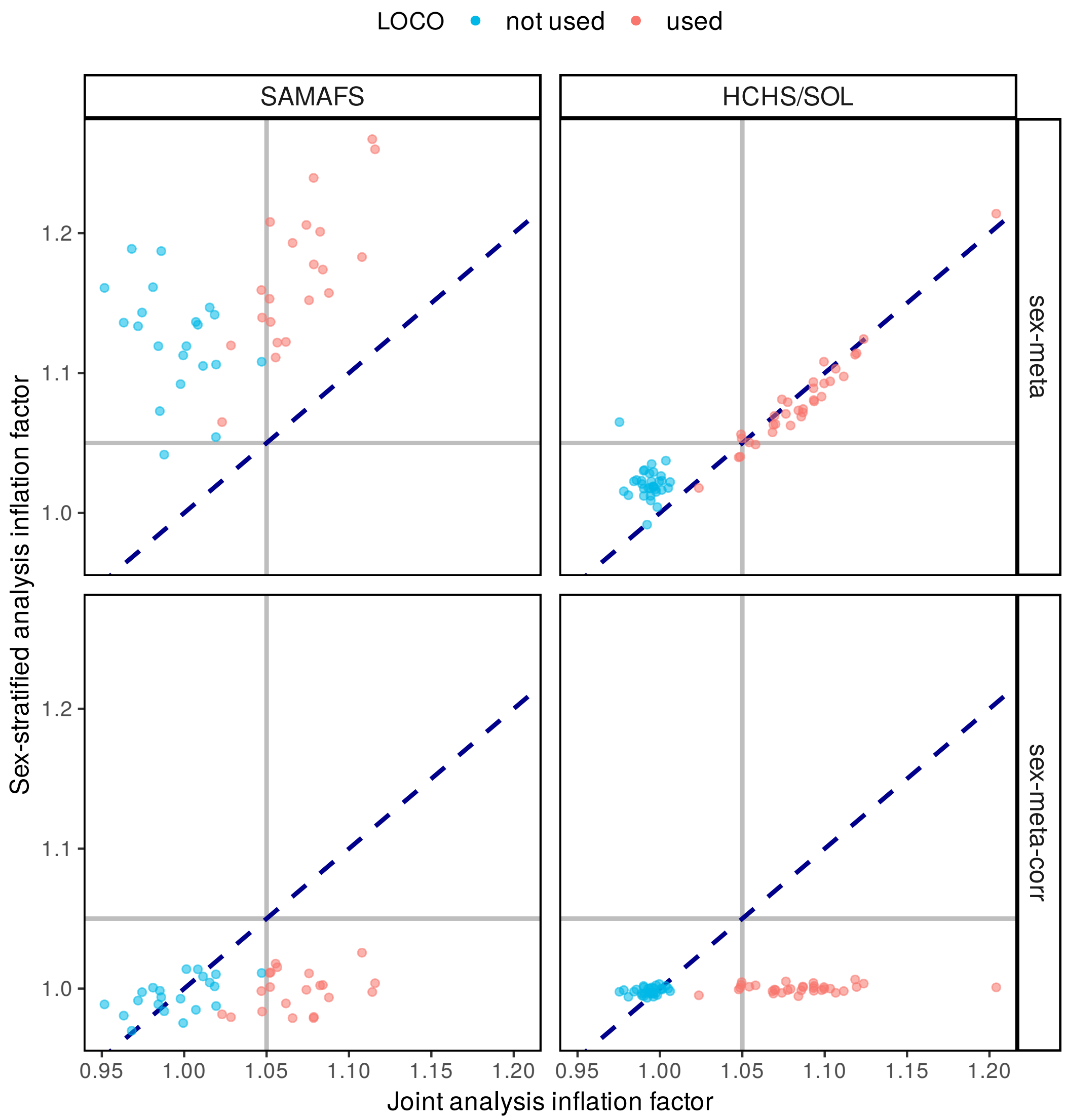

Fig. S11: **Effect of LOCO on inflation factors of joint and sex-stratified meta-analyses.** For joint and sex-meta analyses (top row), inflation factors are greater using LOCO compared to not using it. In SAMAFS, the increases in inflation due to LOCO are such that our main conclusion still holds, namely that meta-analysis has greater inflation compared to the joint analysis (pink points above the $y = x$ diagonal). However, in HCHS/SOL the conclusion no longer holds when using LOCO (pink points are around or below the diagonal). In contrast, sex-meta-corr (bottom row) yields inflation factors close to 1.0, so the correlated meta-analysis method effectively corrects inflated test statistics regardless of use of LOCO.

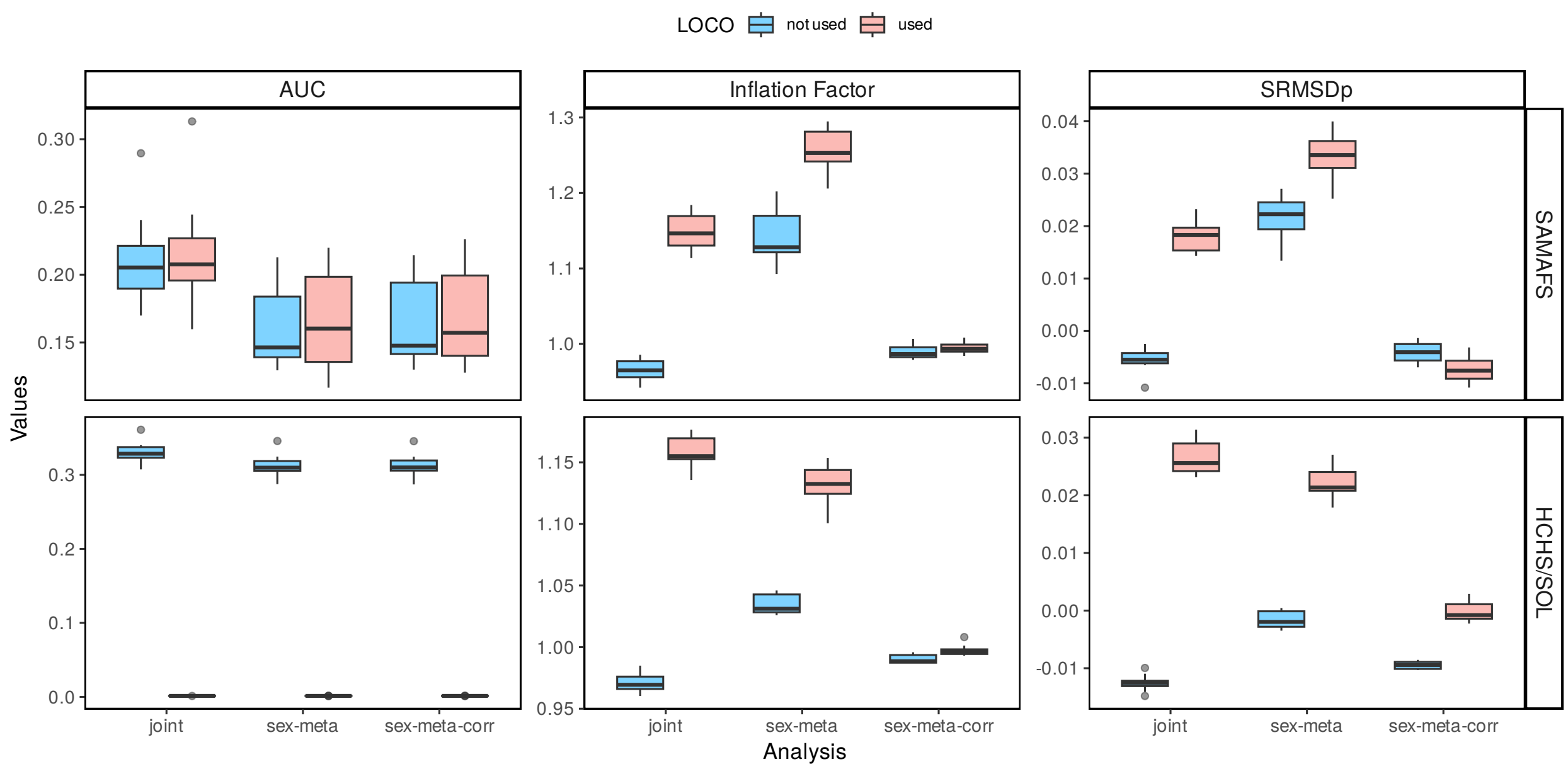

Fig. S12: **Evaluation metrics for LOCO in joint versus sex-stratified meta-analysis on real datasets with simulated traits of 10 replicates.** For both SAMAFS and HCHS/SOL, we observe more favorable metrics when LOCO is not used, with higher AUC_{PR} and lower inflation factors. LOCO inflation is most severe in sex-meta, consistent with our earlier findings. Sex-meta-corr substantially reduces both inflation factor and $SRMSD_p$ relative to sex-meta.

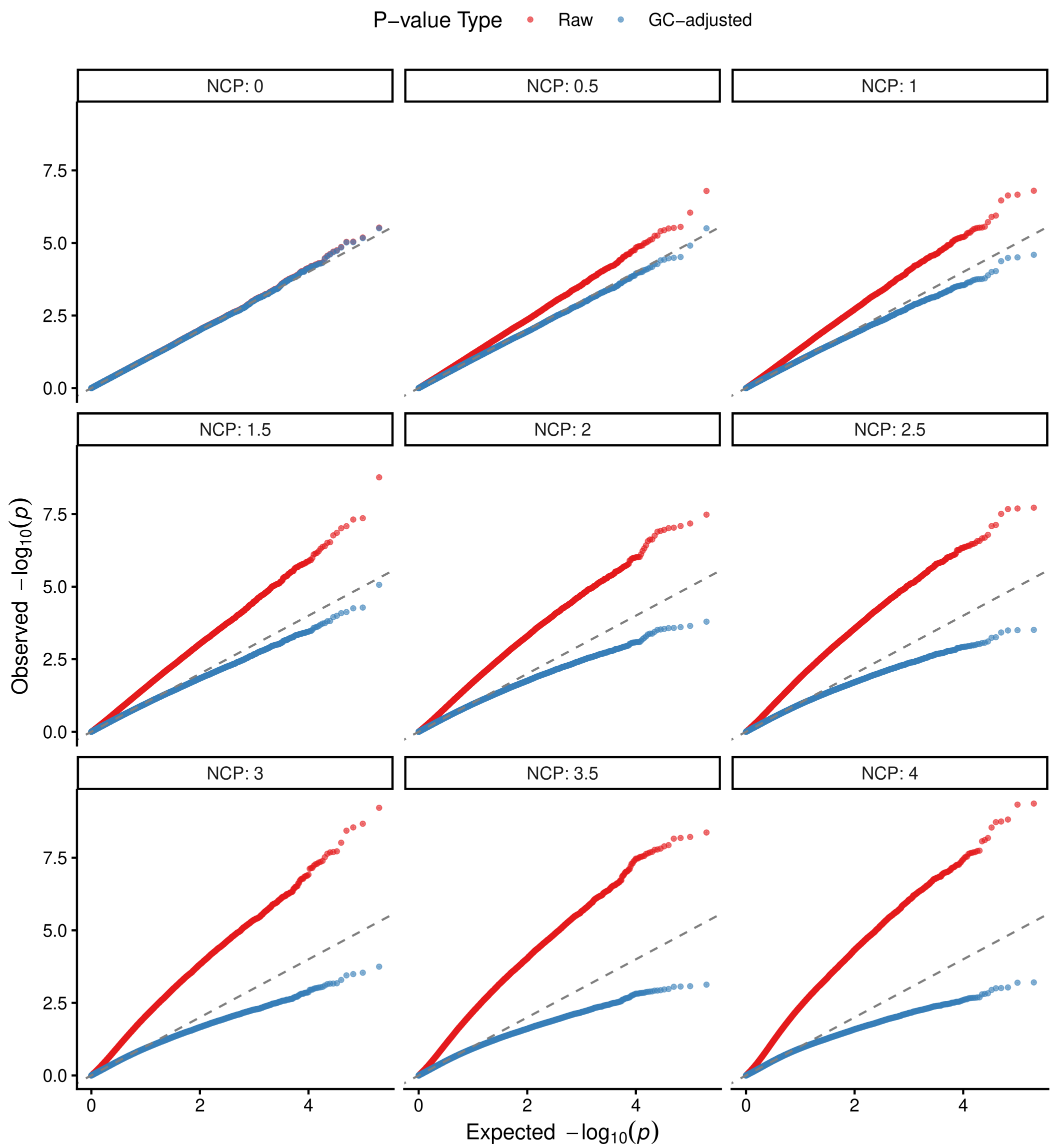

Fig. S13: **Quantile-quantile plots for simulated high polygenicity and high power p-values before and after GC adjustment.** Each panel simulated 200,000 p-values, half from a uniform distribution (null) and half from an alternative distribution, thus mimicking a highly polygenic trait. Alternative test statistics are drawn from $t \sim \chi^2_1(\lambda_{\mathrm{NCP}})$, which is a chi-squared distribution with one degree of freedom and a non-centrality parameter (NCP) λ_{NCP}, then p-values are calculated assuming the null hypothesis $t \sim \chi^2_1(0)$. The NCP increases the mean of the alternative statistics to $1 + \lambda_{\mathrm{NCP}}$ (increasing the resulting power), and is varied by panel: 0 represents a case with no power to detect alternatives, and 4 is very high power. GC overcorrects more strongly when there is higher power, which manifests as a large segments of the curve well below the $y = x$ diagonal (dashed gray line).

Supplemental Tables

Table S1: Traits included in SAMAFS analysis, including data transformation applied.

Trait	Code	Min	Max	Data Transformation
Cystatin C	cystatin_c	0.72	998.45	Log-transformed
2-hour Insulin	a2h_ins	2.4	686.05	Log-transformed
Creatinine	creatinine	35.36	769.08	Log-transformed
Adiponectin	adiponectin	0.48	87.28	Log-transformed
Leptin	leptin	0.01	161.96	Log-transformed
Cholesterol	chol	1.99	9.88	Log-transformed
LDL-cholesterol	ldl	0.51	6.95	Log-transformed
HDL-cholesterol	hdl	0.44	3.21	Log-transformed
Triglycerides	tg	0.33	15.35	Log-transformed
Body mass index	bmi	14.43	69.67	Log-transformed
Hip circumference	hipc	52	230.5	Log-transformed
Waist circumference	wasitc	63.5	223	Log-transformed
Wasit to hip ratio	whr	0.44	1.69	Log-transformed
Diastolic blood pressure	dbp	44	126	Log-transformed
Height	height	121	188.65	None
Fasting glucose	fast_glu	2.35	21.04	Log-transformed
Systolic blood pressure	sbp	92	232.5	Log-transformed
Fasting insulin	fast_ins	1.25	264.21	Log-transformed
2-hour glucose	a2h_glu	2.33	31.91	Log-transformed
Weight	weight	38.33	187.97	Log-transformed
Type 2 diabetes	t2d	0	1	None (binary)

Table S2: Traits included in HCHS/SOL analysis, including data transformation applied.

Trait	Code	Units	Min	Max	Data Transformation
Height	HEIGHT	cm	122	199	None
Body mass index	BMI	kg/m2	14.28	70.35	Log-transformed
Waist to hip ratio	WASIT_HIP	NA	0.52	1.31	Log-transformed
Insulin fasting	INSULIN_FAST	calibrated to mU/L	0.33	726	Log-transformed
Glucose fasting	LABA70	mg/dL	52	642	Log-transformed
Glucose post Oral Glucose Tolerance Test (OGTT)	LABA71	mg/dL	29	389	Log-transformed
Cystatin c.	LABA101	mg/L	0.34	10.1	Log-transformed
High-sensitivity c-reactive protein	LABA91	mg/L	0.11	316.9	Log-transformed
Total cholesterol	LABA66	mg/dL	62	587	Log-transformed
Triglycerides	LABA67	mg/dL	20	6366	Log-transformed
HDL-cholesterol	LABA68	mg/dL	9	141	Log-transformed
LDL-cholesterol	LABA69	mg/dL	22	417	Log-transformed
Weight	ANTA4	kg	34.5	189.2	Log-transformed
Waist girth	ANTA10A	cm	31	202	Log-transformed
Hip girth	ANTA10B	cm	35	306	Log-transformed
Average systolic	SBPA5	mmHg	74	232	Log-transformed
Average diastolic	SBPA6	mmHg	39	135	Log-transformed
Insulin post OGTT	INSULIN_OGTT	calibrated to mU/L	1.5	960.5	Log-transformed
White blood count	LABA1	count × 10e9	1.2	31.8	None
% Neutrophils	LABA10	percentage	0	92	None
% Lymphocytes	LABA11	percentage	5	90	None
% Monocytes	LABA12	percentage	0	47	None
% Eosinophils	LABA13	percentage	0	36	None
% Basophils	LABA14	percentage	0	5.5	None
Red blood cell count	LABA2	count × 10e12	2.21	7.61	Log-transformed
Hemoglobin	LABA3	g/dL	4.3	20.6	Log-transformed
Platelet count	LABA9	count × 10e9	26	874	Log-transformed
Alanine Aminotransferase	LABA74	U/L	5	651	Log-transformed
Aspartate Aminotransferase	LABA75	U/L	5	515	Log-transformed
Gamma-Glutamyl Transferase (GGT)	LABA102	U/L	2	1660	Log-transformed
Ferritin	LABA103	ug/L	7	12745	Log-transformed
Iron	LABA82	ug/dL	8	320	Log-transformed
Apnea/Hypopnea Index	SLPA54	events/hour	0	141.67	Log-transformed

Table S3: Significance of AUC_{PR} overperformance of subpop-meta-corr versus joint.

Num causal	Simulation	Trait type	P-value[a]
100	sim1	Quant	5.08e-04 *
100	sim2	Quant	9.54e-07 *
100	sim3	Quant	6.04e-03
100	sim4	Quant	1.00
100	sim1	Binary	2.38e-05 *
100	sim2	Binary	1.61e-04 *
100	sim3	Binary	0.194
100	sim4	Binary	0.536
5,000	sim1	Quant	1.00
5,000	sim2	Quant	1.00
5,000	sim3	Quant	1.00
5,000	sim4	Quant	1.00
5,000	sim1	Binary	2.93e-04 *
5,000	sim2	Binary	2.93e-04 *
5,000	sim3	Binary	0.478
5,000	sim4	Binary	0.449

[a]All are paired one-sided Wilcoxon tests with 20 replicates. P=1 indicates that joint performed better than subpop-meta-corr.

*Significant at 0.05 level after Bonferroni correction for 16 tests (threshold of 3.13e-03).

Supplemental Acknowledgments

We would like to thank the staff and participants of the SAMAFS and HCHS/SOL study for their important contributions. The research reported in this article was supported by National Institutes of Health grants. The genetic and phenotypic data were provided by the San Antonio Family Heart Study (SAFHS) investigators and supported by the National Heart, Lung, and Blood Institute (NHLBI) [P01 HL045222] and the San Antonio Family Diabetes/Gallbladder Study (SAFDGS) investigators and supported by the National Institute of Diabetes and Digestive and Kidney Diseases (NIDDK) [R01 DK047482, R01 DK053889]. The phenotypic data were also provided by the Veterans Administration Genetic Epidemiology Study (VAGES) investigators and supported by the Health Services Research and Development, U.S., Department of Veteran Affairs and the Family Investigation of Nephropathy and Diabetes (FIND) - San Antonio (FIND-SA) Component and its extension called the Extended FIND (E-FIND) [U01 DK57295] investigators and supported by the NIDDK. The SAFHS gene expression assays were supported by a donation from the Azar and Shepperd families. The exome sequencing, exome chip genotypic, and whole genome sequencing data were provided by the T2D-GENES Consortium grants U01 DK085524, U01 DK085584, U01 DK085501, U01 DK085526, and U01 DK085545 and investigators supported by the NIDDK. This manuscript was not prepared in collaboration with investigators of the T2D-GENES SAMAFS/Consortium and does not necessarily reflect the opinions or views of the members of the T2D-GENES SAMAFS/Consortium, or the NIDDK. The Hispanic Community Health Study/Study of Latinos is supported by contracts from the National Heart, Lung, and Blood Institute (NHLBI) to the University of North Carolina at Chapel Hill, Chapel Hill, NC (N01-HC65233), University of Miami, Miami, FL (N01-HC65234), Albert Einstein College of Medicine, Bronx NY (N01-HC65235), University of Illinois, Chicago IL (N01-HC65236), and San Diego State University, San Diego CA (N01-HC65237). The following Institutes/Centers/Offices contribute to the HCHS/SOL through a transfer of funds to the NHLBI: National Center on Minority Health and Health Disparities, the National Institute of Deafness and Other Communications Disorders, the National Institute of Dental and Craniofacial Research, the National Institute of Diabetes and Digestive and Kidney Diseases, The National Institute of Neurological Disorders and Stroke, and the Office of Dietary Supplements.
