# Supplementary material for: Genetic association meta-analysis is susceptible to confounding by between-study cryptic relatedness
Source: HGG Adv. 2026 Jul 27;7(4):100657. doi: 10.1016/j.xhgg.2026.100657 (PMC13487679; doi:10.1016/j.xhgg.2026.100657)
Supplement: Supplementary file 2 [file mmc2.pdf]

HGG Advances

ARTICLE

Genetic association meta-analysis is susceptible to confounding by between-study cryptic relatedness

Tiffany Tu[1,2,3] and Alejandro Ochoa[1,2,3,4,*]

Summary

Meta-analysis of genome-wide association studies (GWASs) has important advantages, but it assumes that studies are independent, which does not hold when there is relatedness between studies. As a motivating example, recent work suggested applying sex-stratified meta-analysis to correct for participation bias, without considering that men and women from the same population will be highly related. Our theory demonstrates how cryptic relatedness results in correlated test statistics between studies, inflating meta-analysis. We characterize the effects of different between-study relatedness scenarios, particularly population structure and recent family relatedness, on meta-analysis type I error control and power. We simulated data with no family relatedness between subpopulations, family relatedness within subpopulations, family relatedness across subpopulations, and a single population with family relatedness. We evaluated joint GWAS, standard meta-analysis, and our proposed meta-analysis method for correlated studies (R package `metalcor`) on both binary and quantitative traits. In scenarios with family relatedness, standard sex-stratified meta-analysis exhibits severe inflation and lower area under the curve (AUC) than joint and subpopulation meta-analyses, which our method improves by modeling correlation. Genomic control also corrects for inflation but does not alter calibrated power and may fail under high power and high polygenicity. Inflation in standard meta-analysis increases with sample size, which our proposed method avoids. Analysis of real datasets confirms severe inflation for standard sex-stratified meta-analysis in family studies but a negligible effect for population studies with up to 10,000 individuals. Meta-analyses of studies of the same population have increased risk of between-study cryptic relatedness and should be avoided.

Introduction

Meta-analysis of genome-wide association studies (GWASs) combines summary statistics from multiple studies, which routinely increases power compared to a single GWAS, permits analysis without sharing individual-level data, and better accounts for potential heterogeneity between different populations, among other advantages[1,2] (Figure 1A). Population structure due to ancestry differences and cryptic relatedness, which can be understood as ancient and recent (family) relatedness that are unknown to researchers, respectively (Figure 1B), are important confounders in GWASs when they are not adequately modeled.[4,5] Modern GWASs successfully model population structure with principal component analysis (PCA) covariates[6] and address both population structure and cryptic relatedness with linear and logistic mixed-effects models.[3,7–12] However, some meta-analysis designs can result in non-negligible cryptic relatedness between studies, which is an additional confounding factor that cannot be adequately addressed by modeling relatedness solely within studies. Cryptic relatedness between studies violates the independence assumption that meta-analysis makes, as we show here, leading to correlated errors between test statistics of different studies and resulting in increased type I error rates in the meta-analysis. Thus, confounding can occur in the meta-analysis even when the input studies are not individually confounded.

The importance of sample independence is well known to statistical geneticists, and it is recommended to adjust for population stratification and to identify sample overlap between studies before carrying out meta-analysis.[1] Despite these guidelines, there is still limited attention on addressing cryptic relatedness between studies in meta-analyses. For example, a recent study recommended performing sex-stratified meta-analysis (sex-meta) to correct for sex-differential participation bias, without considering the potential violation of independence between the male and female studies.[13] Meta-analysis does not allow direct modeling of relatedness between studies, but there are potential alternatives. The ideal solution is joint GWAS of the substudies (or mega-analysis), which effectively handles population structure and cryptic relatedness, but it requires access to individual data, which can be a major hindrance. Federated learning privacy approaches are recent proposals addressing this problem, performing joint analyses of data across institutes without sharing individual-level data; instead, they perform calculations using encrypted genotypes to both detect genetic relatedness across cohorts[14–16] and perform association tests.[17–19] A recent study compared joint (or mega) GWAS to meta-analysis in a multi-ancestry setting using data from the Hyperglycemia and Adverse Pregnancy Outcome (HAPO) study and found

[1]Program of Computational Biology and Bioinformatics, Duke University, Durham, NC, USA; [2]Department of Biostatistics and Bioinformatics, Duke University, Durham, NC, USA; [3]Duke Center for Statistical Genetics and Genomics, Duke University, Durham, NC, USA
[4]Lead contact
*Correspondence: alejandro.ochoa@duke.edu
https://doi.org/10.1016/j.xhgg.2026.100657.
© 2026 The Author(s). Published by Elsevier Inc. on behalf of American Society of Human Genetics.
This is an open access article under the CC BY license (http://creativecommons.org/licenses/by/4.0/).

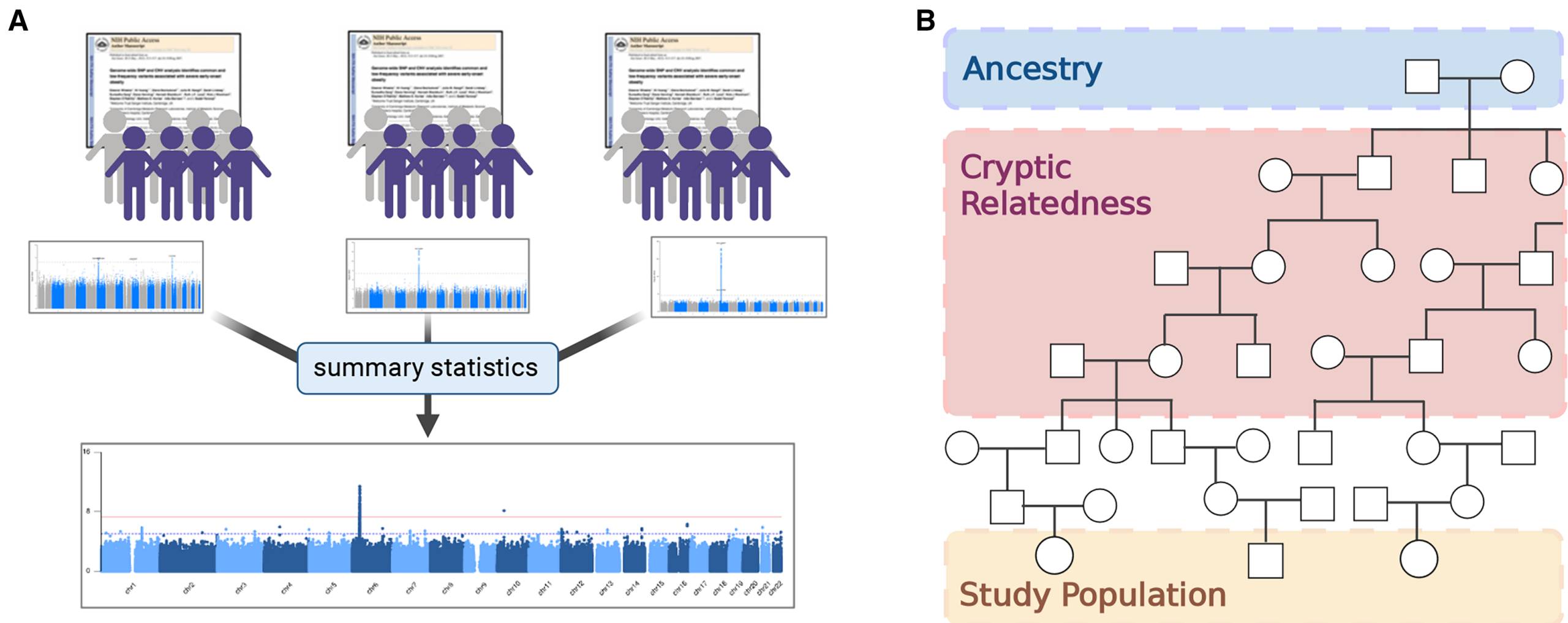

Figure 1. Cryptic relatedness can cause inflation in GWAS meta-analysis
(A) Illustration of a standard GWAS meta-analysis pipeline, which combines summary statistics from multiple studies while assuming data independence. Population structure due to ancestry differences and cryptic relatedness are both common causes of dependence between individuals in GWAS.
(B) Different degrees of relatedness visualized through a pedigree. Cryptic relatedness is a form of family relatedness that is recent but unknown to the researchers. Family structure is high dimensional and must be modeled with linear mixed-effects models in GWAS.[3] In contrast, ancestry is a more ancient form of relatedness that is broadly shared across the population, so it can be modeled with low-dimensional models such as PCA. This figure was created with BioRender.com.

that joint GWAS has more variability in genomic inflation but improved power and identified more significant genetic associations.[20] Simulations and real data from the UK Biobank and All of Us Research Programs demonstrate that joint (or pooled) analysis exhibits better statistical power while effectively adjusting for population stratification.[21] Lastly, output statistics could be adjusted using genomic control (GC), which calibrates the median statistic to its expected value.[4,10,22–25] However, while GC was originally proposed to correct for population stratification, it is often unsuccessful,[6,7,25–27] and it has been studied even less for correcting for cryptic relatedness.

In this work, we demonstrate that cryptic relatedness between studies can cause confounding in standard meta-analysis and propose a meta-analysis method that accounts for correlations between studies. We simulate data with combinations of population and family structure and identify family structure as the only severe source of confounding. Our method successfully eliminates the inflation present in standard meta-analysis but does not usually improve calibrated power. We also find that GC can correct inflated meta-analysis results in small studies without improving loss of calibrated power, and GC is expected to overcorrect for highly polygenic traits under high statistical power. Analysis of real human datasets confirms our findings, with strong confounding observed in family studies, although the effect is negligible in population studies. Furthermore, both theory and simulations show that inflation for standard sex-meta grows steadily with sample size, while our proposed meta-analysis method for correlated studies maintains calibration across all simulation scenarios. As sample sizes grow with modern biobank-scale studies, cryptic relatedness will become an increasingly severe confounder of standard meta-analysis, a problem our method addresses.

Methods

Genetic association method

Joint and stratified GWASs are performed using SAIGE (scalable and accurate implementation of generalized mixed model), a linear and logistic mixed model that controls for unbalanced case-control ratio and sample relatedness at efficient runtime and scalability.[11] The only exception is a simulation without any structure (simulation 1 below, subpopulation-stratified quantitative trait analysis only), which required a standard linear regression performed with PLINK2 `glm`,[28] since here the kinship matrix is approximately a multiple of the identity matrix, so linear mixed models have ill-defined variance components and often fail to converge. The approach for quantitative traits is a linear model (shown below), while for binary traits, a logistic mixed model is used instead (equation not shown). Each of our GWASs incorporates as covariates sex, age (for real datasets), and subpopulation label (for simulated datasets), if they are not constant, along with the top 10 principal components (PCs) of the genotype matrix. Main analyses were performed without using the leave-one-chromosome-out (LOCO) option, while supplemental analyses for the real datasets used LOCO when indicated. We use METAL[29] to perform all of our "standard" fixed-effects GWAS meta-analyses.

The linear mixed-effects model (LMM) used at SNP i for a given study j with n_j individuals is given by

$$\begin{aligned} \mathbf{y}_j &= \mathbf{1}_{n_j}\alpha + \mathbf{x}_{ij}\beta_{ij} + \mathbf{s} + \boldsymbol{\varepsilon}, \\ \mathbf{s} + \boldsymbol{\varepsilon} &\sim \text{Normal}\left(\mathbf{0}, \sigma_y^2\mathbf{V}_j\right), \\ \mathbf{V}_j &= 2h^2\boldsymbol{\Phi}_j + \left(1 - h^2\right)\mathbf{I}_{n_j}, \end{aligned} \quad \text{(Equation 1)}$$

where $\mathbf{y}_j$ is a length-n_j vector of individual trait values, $\mathbf{1}_{n_j}$ is a length-n_j vector of ones, α is a scalar of intercept coefficients, $\mathbf{x}_{ij}$ is the length-n_j vector of genotypes at SNP i, β_{ij} is the genetic effect coefficient, $\mathbf{s}$ is a length-n_j structured residual vector, $\boldsymbol{\varepsilon}$ is a length-n_j vector of residual errors, σ_y^2 is a trait-specific variance factor, $\mathbf{V}_j$ is the $n_j \times n_j$ scale-free covariance matrix of the trait, h^2 is the heritability, $\boldsymbol{\Phi}_j$ is the $n_j \times n_j$ kinship matrix, and $\mathbf{I}_{n_j}$ is the $n_j \times n_j$ identity matrix. For simplicity, covariates are omitted in this formulation. The genetic effect coefficient $\widehat{\beta}_{ij}$ and its standard error $\widehat{\sigma}_{ij}$ are estimated from this model as shown in Appendix A.

Theoretical cause of inflation in standard association meta-analysis due to between-study relatedness

For the following derivations, we will assume the fixed-effects meta-analysis framework implemented by METAL.[29] Suppose that there are S studies we wish to meta-analyze; each study $j \in \{1, \ldots, S\}$ with estimated coefficients $\widehat{\beta}_{ij}$ and standard errors $\widehat{\sigma}_{ij}$ at SNP i. The Z scores are

$$z_{ij} = \frac{\widehat{\beta}_{ij}}{\widehat{\sigma}_{ij}}.$$

If the individual studies are calibrated, under the null hypothesis of no association, we have $\mathrm{E}[z_{ij}] = 0$ and $\mathrm{Var}(z_{ij}) = 1$. The combined Z score z_i is the average per-study Z score weighted by the per-study standard error, namely

$$\frac{1}{\widehat{\sigma}_i^2} = \sum_{j=1}^{S} \frac{1}{\widehat{\sigma}_{ij}^2},$$

$$z_i = \sum_{j=1}^{S} \frac{\widehat{\sigma}_i}{\widehat{\sigma}_{ij}} z_{ij}.$$

When studies are calibrated and independent of each other, the combined Z score is also calibrated. In particular, treating z_{ij} as random but $\widehat{\sigma}_{ij}$ as fixed for simplicity, it follows under the null hypothesis that

$$\mathrm{E}[z_i] = \sum_{j=1}^{S} \frac{\widehat{\sigma}_i}{\widehat{\sigma}_{ij}} \mathrm{E}[z_{ij}] = 0,$$

$$\mathrm{Var}(z_i) = \sum_{j=1}^{S} \frac{\widehat{\sigma}_i^2}{\widehat{\sigma}_{ij}^2} \mathrm{Var}(z_{ij}) = 1.$$

However, when studies have correlated statistics, the combined variance exceeds 1, making these statistics inflated even if the individual studies are calibrated:

$$\begin{aligned}\mathrm{Var}(z_i) &= \sum_{j=1}^{S} \sum_{k=1}^{S} \frac{\widehat{\sigma}_i^2}{\widehat{\sigma}_{ij}\widehat{\sigma}_{ik}} \mathrm{Cov}(z_{ij}, z_{ik}) \\ &= 1 + 2\sum_{j=2}^{S} \sum_{k=1}^{j-1} \frac{\widehat{\sigma}_i^2}{\widehat{\sigma}_{ij}\widehat{\sigma}_{ik}} \mathrm{Cov}(z_{ij}, z_{ik}).\end{aligned}$$

In Appendix A, we derive a complete form for $\mathrm{Cov}(z_{ij}, z_{ik})$ under a joint LMM as the true model, which has a complicated dependence on the kinship matrix between studies j and k, the trait heritability h^2, and the model trait covariance matrices $\mathbf{V}_j$ of each separate study j. Treating the relatedness values between studies as random and independent, the covariance term that leads to inflation has the approximate form

$$\begin{aligned}\mathrm{Cov}(z_{ij}, z_{ik}) &= r_{jk} \approx 4h^2\sigma_\varphi^2\sqrt{f(\mathbf{V}_j)f(\mathbf{V}_k)}, \\ f(\mathbf{V}_j) &= \frac{h^2\mathrm{Tr}(\mathbf{M}_j)^2}{n_j - 1 - (1 - h^2)\mathrm{Tr}(\mathbf{M}_j)}, \\ \mathbf{M}_j &= \mathbf{V}_j^{-1} - \frac{\mathbf{V}_j^{-1}\mathbf{1}_{n_j}\mathbf{1}_{n_j}'\mathbf{V}_j^{-1}}{\mathbf{1}_{n_j}'\mathbf{V}_j^{-1}\mathbf{1}_{n_j}},\end{aligned} \qquad \text{(Equation 2)}$$

where σ_φ^2 is the variance in between-study kinship values and $f(\mathbf{V}_j) \geq n_j$ is a function (derived in Appendix A) that empirically scales with study j sample size (linearly for population structure and superlinearly under within-study cryptic relatedness) and further increases with heritability and study j's F_{ST} (Figure 2). Each factor of the covariance is non-negative, proving that relatedness between studies results in inflation upon standard meta-analysis. Further, for a fixed amount of cryptic relatedness variance between studies σ_φ^2, set indirectly via a threshold on kinship estimates, for example, inflation will grow with sample size, so very distant relatives could result in substantial inflation for meta-analysis of large, biobank-scale datasets.

We next empirically characterized basic properties of the variance in between-study kinship values σ_φ^2 using the same family simulation 4 described later in the methods. Here, we only simulated random pedigrees and their true kinship matrices for the last of $G = 30$ generations; let N denote the population size and n the sample size of a random subset of the population. For each such subset, we randomly split it into two "studies," extracted the kinship matrix between studies, and calculated the mean and variance of those kinship values. We repeated this for 10 replicates each of combinations of $N \in \{1{,}000, 2{,}000, \ldots, 10{,}000\}$ and $n \in \{100, 200, \ldots, N\}$. We found that these kinship means and variances depend on the population size N but not on sample sizes n (Figure S1). Therefore, for two studies drawn from the same given population with a fixed demographic history, we can expect σ_φ^2 to be fixed, resulting in excess inflation (r_{jk}) increasing proportionally to study size, as a rough approximation.

Our proposed meta-analysis method for correlated studies: `metalcor`

Under the model we just derived (both the approximation in Equation 2 above and the more precise form in Equation A2 in Appendix A), the covariance r_{jk} between Z scores of studies j and k is the same at all SNPs i, so it can be estimated from summary statistics only. In the next subsection, we present methods for estimating r_{jk}; for now, assume these values are available. Switching to matrix notation, the model under study's covariance is

$$\begin{aligned}\mathrm{Cov}(\mathbf{z}_i) &= \mathbf{R}, \\ \mathrm{E}[\widehat{\boldsymbol{\beta}}_i] &= \beta_i\mathbf{1}_S, \\ \mathrm{Cov}(\widehat{\boldsymbol{\beta}}_i) &= \boldsymbol{\Sigma}_i,\end{aligned}$$

where $\mathbf{z}_i = (z_{ij})$ and $\widehat{\boldsymbol{\beta}}_i = \left(\widehat{\beta}_{ij}\right)$ are length-S vectors, $\mathbf{R} = (r_{jk})$ and $\boldsymbol{\Sigma}_i = \left(r_{jk}\widehat{\sigma}_{ij}\widehat{\sigma}_{ik}\right)$ are $S \times S$ matrices, and the covariance for regression coefficients follows from that of Z scores, as they are related by the fixed factors $\widehat{\sigma}_{ij}$ and $\widehat{\sigma}_{ik}$. The expectation follows since coefficient estimates are unbiased, where β_i is the true coefficient at SNP I, and it is the same across studies. Note that the diagonal values of $\mathbf{R}$ are 1 when input studies are calibrated, but we estimate these values as well in order to correct for miscalibration if present.

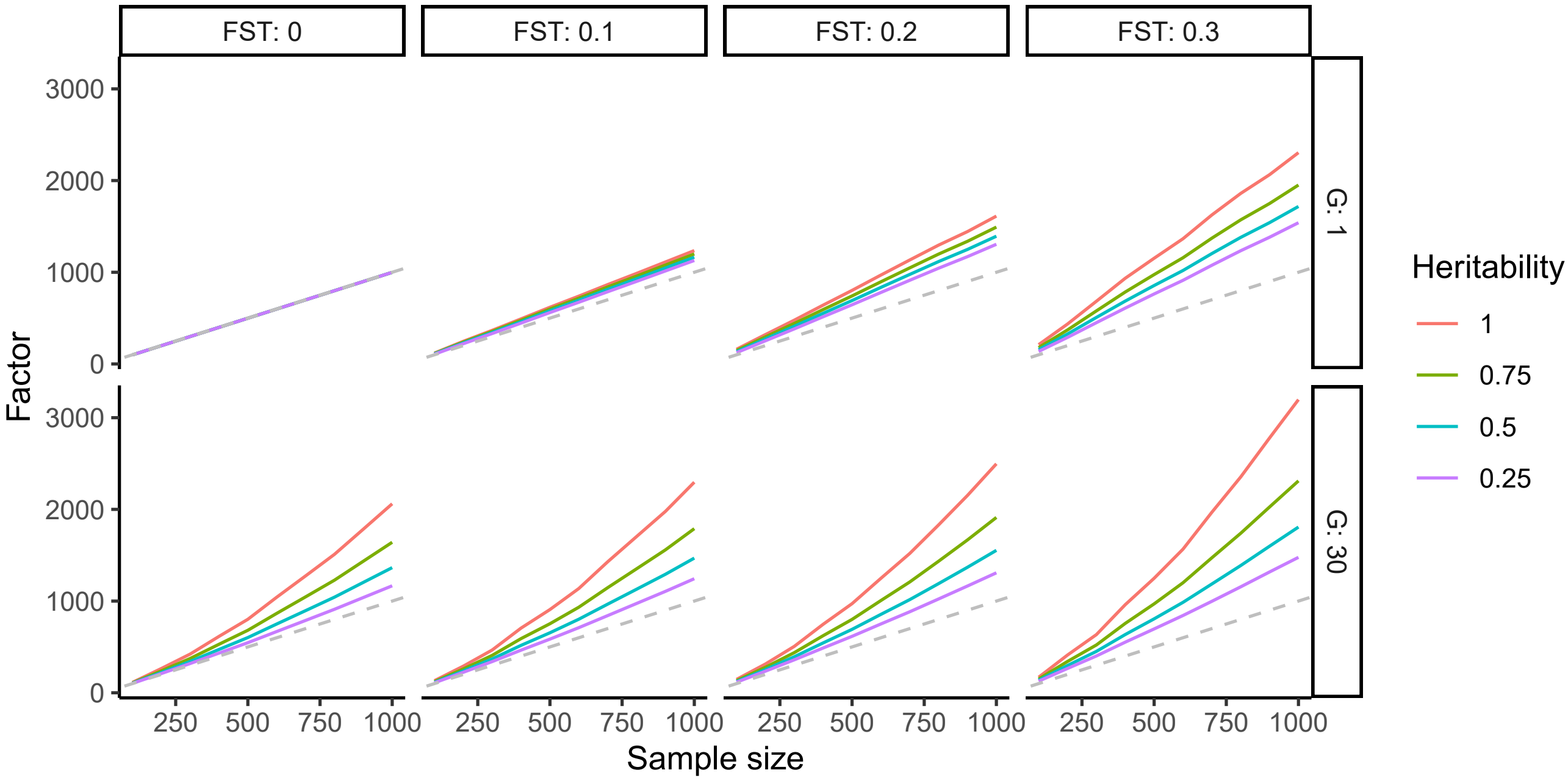

Figure 2. Dependence of within-study factor on sample size, population and family structure, and heritability
We plotted n_j versus the within-study factor $f(V_j)$ in Equation 2 for a single study j, separately for combinations of F_{ST}, heritability, and family structure ($G = 1$ versus 30 generations). The dashed gray line is $y = x$. For each combination of G and F_{ST}, kinship matrices Φ_j were constructed for $n = 1{,}000$ individuals from the one-dimensional admixture model used in Ochoa and Storey.[30] followed by G generations of family structure (methods), then $V_j = 2h^2\Phi_j + \left(1 - h^2\right)I_{n_j}$ was calculated according to the desired heritability. Lower sample sizes were obtained by subsampling.

The proposed meta-analysis model that accounts for correlation between studies is the multivariate normal approximation of our above model,

$$\widehat{\boldsymbol{\beta}}_i \sim \text{Normal}(\beta_i \mathbf{1}_S, \boldsymbol{\Sigma}_i),$$

and the meta-analyzed result is simply the maximum likelihood estimate of β_i under this model, again treating $\boldsymbol{\Sigma}_i$ as fixed, which is also the best linear unbiased estimator of β_i under covariance,[31]

$$\widehat{\beta}_i = \frac{\mathbf{1}_S' \boldsymbol{\Sigma}_i^{-1} \widehat{\boldsymbol{\beta}}_i}{\mathbf{1}_S' \boldsymbol{\Sigma}_i^{-1} \mathbf{1}_S},$$

where the prime (') denotes matrix transposition. The variance of this meta-analyzed estimate is

$$\begin{aligned}\widehat{\sigma}_i^2 &= \text{Var}(\widehat{\beta}_i) \\ &= \text{Var}\left(\frac{\mathbf{1}_S' \boldsymbol{\Sigma}_i^{-1} \widehat{\boldsymbol{\beta}}_i}{\mathbf{1}_S' \boldsymbol{\Sigma}_i^{-1} \mathbf{1}_S}\right) \\ &= \frac{\mathbf{1}_S' \boldsymbol{\Sigma}_i^{-1}}{\mathbf{1}_S' \boldsymbol{\Sigma}_i^{-1} \mathbf{1}_S} \text{Cov}(\widehat{\boldsymbol{\beta}}_i) \frac{\boldsymbol{\Sigma}_i^{-1} \mathbf{1}_S'}{\mathbf{1}_S' \boldsymbol{\Sigma}_i^{-1} \mathbf{1}_S} \\ &= \frac{\mathbf{1}_S' \boldsymbol{\Sigma}_i^{-1} \boldsymbol{\Sigma}_i \boldsymbol{\Sigma}_i^{-1} \mathbf{1}_S'}{\left(\mathbf{1}_S' \boldsymbol{\Sigma}_i^{-1} \mathbf{1}_S\right)^2} \\ &= \frac{1}{\mathbf{1}_S' \boldsymbol{\Sigma}_i^{-1} \mathbf{1}_S}.\end{aligned}$$

Thus, as long as the covariance $\boldsymbol{\Sigma}_i$ of the study coefficients is well estimated, this method calculates $\widehat{\sigma}_i$ correctly and results in calibrated meta-analyzed Z scores $z_i = \widehat{\beta}_i / \widehat{\sigma}_i$ and also their resulting p values assuming the null hypothesis $z_i \sim \chi_1^2$. Furthermore, since the above is also the best linear unbiased estimator of the coefficients, it has less variance than the standard estimate when there is covariance.

It is straightforward to show that when $\mathbf{R} = \mathbf{I}$, these estimators are identical to those of standard meta-analysis. In other words, inverse variance weighting is implemented as a special case. Therefore, our method generalizes standard meta-analysis for correlated studies. This method, including the estimation of study covariance presented in the next subsection, is implemented in the R package `metalcor`, available on the Comprehensive R Archive Network (CRAN).

Estimation of study covariance matrix

We present two estimators for $\mathbf{R}$ from Z scores: a simple one that calculates mean Z score product values and a more elaborate approach that uses medians instead, following the intuition from GC.

The mean-based estimator assumes that the whole genome satisfies the null hypothesis of no association in all studies, in which case $E[z_{ij}] = 0$, so the covariance of interest also equals $r_{jk} = E[z_{ij} z_{ik}]$. Thus, the naive estimate is given by

$$\widehat{r}_{jk} = \frac{1}{m} \sum_{i=1}^{m} z_{ij} z_{ik},$$

where m is the number of SNPs. However, under the alternative hypothesis, this overestimates the covariance because true coefficients will incorrectly contribute to the estimate, and the bias will depend on the proportion of the genome that is causal variants or in linkage disequilibrium (LD) with them.

The median-based estimator is more robust than the mean version because it ignores true associations more effectively, as they tend to have larger Z scores and thus are high outliers, as

long as polygenicity is moderate and power is not extremely high. However, the median value of $z_{ij}z_{ik}$ does not equal the mean because its distribution is skewed with a heavy tail, so additional calculations are needed to adjust this median to yield an estimate of r_{jk}. Assume the null hypothesis and calibration within studies, namely

$$\begin{pmatrix} z_{ij} \\ z_{ik} \end{pmatrix} \sim \text{Normal}\left(\begin{pmatrix} 0 \\ 0 \end{pmatrix}, \begin{pmatrix} 1 & \rho \\ \rho & 1 \end{pmatrix}\right).$$

The probability density function for the product random variable $x = z_{ij}z_{ik}$ (domain in all real numbers) is given by[32]

$$f(x;\rho) = \frac{1}{\pi\sqrt{1-\rho^2}}\exp\left(\frac{\rho x}{1-\rho^2}\right)\mathrm{K}_0\left(\frac{|x|}{1-\rho^2}\right),$$

where K_0 is the modified Bessel function of the second kind of order zero. Note that $f(x;-\rho) = f(-x;\rho)$, so without loss of generality, we can assume $\rho > 0$. For large values of $|x|$, and for ρ near 1, we encountered NaN values (when the exponential evaluates to zero while K_0 returns infinity), which were successfully avoided using the asymptotic approximation[33] $\mathrm{K}_0(y) \approx \sqrt{\frac{\pi}{2y}}\exp(-y)$ as $y \to \infty$, which results for $\rho > 0$ in

$$f(x;\rho) \approx \begin{cases} \frac{1}{\sqrt{2\pi x}}\exp\left(\frac{-x}{1+\rho}\right) & \text{if} \quad x \geq 0, \\ 0 & \text{if} \quad x < 0. \end{cases}$$

This approximation was only employed when the explicit formula failed. The cumulative probability distribution (CDF) was calculated by numerical integration, and the median was calculated from the CDF using a root finder (for the value x that has a CDF of 0.5). The median x is a function of ρ, so the final estimate of r_{jk} is the value of ρ whose median equals the sample median, which is also found using a root finder. As ρ approaches 1, where $z_{ij} = z_{ik}$ and thus $x \sim \chi^2_1$, values larger than the χ^2_1 median are simply divided by that median, yielding the same inflation factors that GC calculates for continuity. Thus, this formula is also applied when $j = k$ and can return values of $r_{jj} \neq 1$, in which case our method also corrects the inflation or deflation in the input studies. The final curve mapping product medians x to covariance estimates is shown in Figure S2.

Inflation factor and the GC method

A common metric to assess confounding due to population stratification in GWASs, or statistical miscalibration more broadly, is the inflation factor λ, defined as the median test statistic across SNPs divided by the theoretical median under the expected null χ^2 distribution.[4,10,22–25] Calibrated statistics have $\lambda \approx 1$, while $\lambda > 1.05$ is considered inflated.[10] The GC method consists of dividing the test statistics by the inflation factor, thus ensuring that the inflation factor is exactly one after adjustment,[4] although this does not guarantee calibration at other quantiles. Despite this potential limitation, we consider GC as a possible solution to meta-analysis inflation caused by relatedness between studies.

Genotype and trait simulations

Genotypes with population and family structure are simulated using the R packages `bnpsd` and `simfam`, respectively, following previous work.[3,30] Briefly, an ancestral population T has allele frequencies for 500,000 SNPs drawn from a uniform distribution, then subpopulations S_i for $i \in \{1, 2, 3\}$ have allele frequencies drawn from the Balding-Nichols model,[34] from which genotypes are drawn binomially for 1,000 individuals per subpopulation (3,000 in total). We generate correlated S_2 and S_3 by drawing them from a common ancestor population, denoted S_{23}, while S_1 is simulated independently. Subpopulations S_1 and S_{23} both have an inbreeding coefficient from T of $f^T_{S_i} = 0.18$. Subpopulations S_2 and S_3 have $f^{S_{23}}_{S_i} = 0.22$ from their common ancestor, resulting in a total inbreeding coefficient from T of $f^T_{S_i} = 1 - \left(1 - f^T_{S_{23}}\right)\left(1 - f^{S_{23}}_{S_i}\right) = 0.36$ (formula from Ochoa and Storey[30]). The average inbreeding coefficient across the combined population is $F_{\mathrm{ST}} = 0.3$. Sex is drawn randomly for each individual.

When there is family structure, pedigrees are constructed by randomly pairing individuals of opposite sexes, biased by proximity in their one-dimensional coordinates (initially contiguous by subpopulation), with variable numbers of children constrained to a total desired population size, which is fixed throughout the generations. Founders have genotypes drawn from the same population structure described above. Child genotypes are drawn from their parents' genotypes following Mendelian inheritance, independently per SNP.

To characterize the effect of between-study relatedness scenarios, we considered four simulations: (1) no family relatedness across 3 subpopulations or otherwise 30 generations of family relatedness with these configurations: (2) within subpopulations, (3) across subpopulations, and (4) in a single population (Figure 3). Since simulation 4 has a single population (with 3,000 individuals in total, matching the rest), for testing subpop-meta, it is partitioned into three subpopulations of 1,000 individuals each grouped by their one-dimensional coordinates.

Quantitative traits for the simulated genotypes are simulated using the R package `simtrait`, following a fixed-effect size (FES) model for the coefficients with $h^2 = 0.8$ and 100 (main results) or 5,000 (supplement) causal loci, as before.[3] Binary traits are generated by thresholding the previous quantitative traits at their median. We took this measure because, due to the strong population structure, case-control ratios in subpopulations ended up differing greatly from the global 1:1 ratio, and the problem was much worse at more skewed global ratios. Traits are resimulated for a replicate if SAIGE does not converge, which occurs if case-control ratios in a given subpopulation are extremely imbalanced. (Real biobanks have very skewed ratios but larger total case numbers than our simulations, which are handled well by methods such as SAIGE, which was specifically developed for this purpose.[11]) For LOCO evaluations using real genotypes (San Antonio Mexican American Family Study [SAMAFS] and the Hispanic Community Health Study/Study of Latinos [HCHS/SOL]), we simulated quantitative traits with 100 causal loci for SAMAFS and 1,000 causal loci for HCHS/SOL, which were selected to scale roughly with sample size as before.[3]

To test the relationship between inflation and sample size, we extended simulation 4 (single population of 30 generations) to 10,000 individuals and ran 5 replicates. For each replicate, we drew subsets of n = 8,000, 6,000, 4,000, and 2,000 individuals and repeated the joint and sex-stratified meta-analyses at each sample size.

Evaluation metrics

Our simulations are evaluated using (1) the inflation factor λ defined earlier, (2) $\mathrm{AUC_{PR}}$ (precision-recall area under the curve),

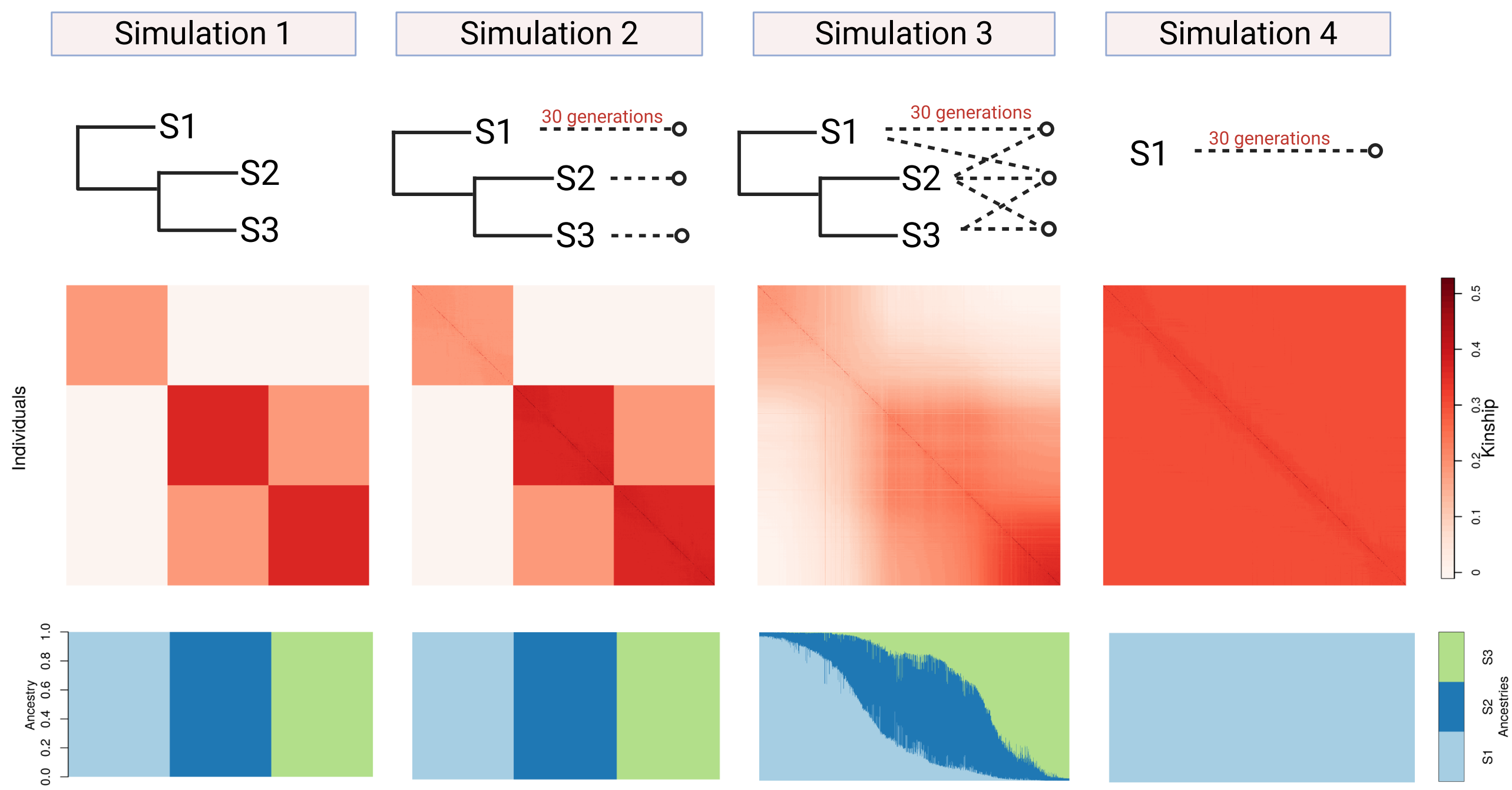

Figure 3. Overview of the population and family structure of simulation scenarios
Top row: phylogenetic trees summarize the key features of each relatedness structure. Population and family structure are represented by solid and dashed lines, respectively. Middle row: kinship matrices show the covariance structure between each pair of individuals (along both x and y axes, using the same order of the underlying one-dimensional space of the simulations), where color represents their total kinship coefficient (reflecting both population and family relatedness). Diagonal plots show inbreeding coefficients instead of self-kinship since inbreeding is on the same scale as the rest of the kinship values. Note that family structure results in more high-kinship pairs that appear near the diagonal of simulations 2–4. Bottom row: admixture proportions better summarize the population structure in these data, ignoring family structure. Each individual (x axis) has a stacked barplot of ancestry proportions (colors) that sums to one. Only simulation 3 has admixture (individuals with more than one ancestry).

and (3) SRMSD_p (p value signed root-mean-square deviation), following earlier work.[3] Briefly, we use the R package PRROC[35] to compute $\mathrm{AUC}_{\mathrm{PR}}$, where given the total number of true positives (TPs), false positives (FP), and false negatives (FNs) at some p value threshold t,

$$\mathrm{Precision}(t) = \frac{\mathrm{TP}(t)}{\mathrm{TP}(t) + \mathrm{FP}(t)},$$

$$\mathrm{Recall}(t) = \frac{\mathrm{TP}(t)}{\mathrm{TP}(t) + \mathrm{FN}(t)}.$$

The resulting AUC, $\mathrm{AUC}_{\mathrm{PR}}$, measures causal locus classification accuracy and reflects calibrated statistical power while being robust to miscalibrated models.[3]

SRMSD_p measures the difference between the observed null p value quantiles and the expected uniform quantiles:

$$\mathrm{SRMSD}_p = \mathrm{sgn}\left(u_{\mathrm{median}} - p_{\mathrm{median}}\right)\sqrt{\frac{1}{m_0}\sum_{i=1}^{m_0}\left(u_i - p_{(i)}\right)^2},$$

where m_0 is the number of null (non-causal) loci, $p_{(i)}$ is the ith-order null p value, $u_i = (i - 0.5)/m_0$ is its expectation, p_{median} is the median observed null p value, $u_{\mathrm{median}} = \frac{1}{2}$ is its expectation, and sgn is the sign function. Analogous to the $0.95 < \lambda < 1.05$ rule of thumb, $|\mathrm{SRMSD}_p| < 0.01$ is considered calibrated.[3] SRMSD_p is a stricter test of calibration than the inflation factor, since the ideal $\mathrm{SRMSD}_p = 0$ requires null p value uniformity at all quantiles, whereas the ideal $\lambda = 1$ merely requires the median to match its null expectation. However, since both $\mathrm{AUC}_{\mathrm{PR}}$ and SRMSD_p require knowing truly causal loci, they cannot be applied to real data. In contrast, the inflation factor can be computed directly from the observed summary statistics in real datasets.

The $\mathrm{AUC}_{\mathrm{PR}}$ and SRMSD_p statistics implicitly assume no LD between causal and non-causal variants, so minor adjustments are needed to apply them to simulated traits using real genotypes with LD, such as our LOCO evaluations using SAMAFS and HCHS/SOL. In particular, we masked every non-causal SNP within 1 Mb of a causal variant, so those SNPs are treated neither as TPs nor FPs, before calculating our statistics. In contrast, all SNPs were used to calculate inflation factors, to match how inflation is measured in practice.

Real datasets

The secondary data analysis of dbGaP datasets was reviewed by the Duke University Health System (DUHS) Institutional Review Board (IRB) and was determined to be exempt from full review (Pro00108518).

T2D-GENES SAMAFS consortium

We analyzed 914 individuals (380 males and 534 females) for 21 traits from the SAMAFS Project 2,[36] using exome chip data to maximize the number of individuals from 20 large Mexican American pedigrees (dbGaP: phs000847.v2.p1). Starting from 79,272 SNPs, we applied filters of Hardy-Weinberg equilibrium (HWE) $p < 1\mathrm{e}{-10}$ and a minor-allele frequency (MAF) < 0.01, resulting in 36,293 SNPs. A total of 20 quantitative traits and one binary trait (type 2 diabetes) were analyzed (Table S1). Quantitative traits were log transformed where appropriate to improve normality.

HCHS/SOL

We analyzed 11,721 individuals (6,831 males and 4,890 females) from the HCHS/SOL[37] (dbGaP: phs000810.v2.p2) using array data from substudy `SOL_phaseIa_genotyping`. We applied HWE $p < 1\text{e}{-10}$ and MAF < 0.01 filters, resulting in 1,651,164 SNPs. A total of 33 quantitative traits were analyzed and transformed as needed (Table S2).

Results

Meta-analysis inflation caused by between-study cryptic relatedness confirmed in simulated data

In all of our evaluations, we contrast three types of studies. The joint analysis fully accounts for population structure and cryptic relatedness and is taken as the baseline for good performance. The subpopulation-stratified meta-analysis (subpop-meta) is a common study design where individual studies are performed in single populations before combining resulting summary statistics for meta-analysis. Subpop-meta, also known as trans-ethnic meta-analysis, potentially handles cryptic relatedness successfully, as distant family members are expected primarily within subpopulations rather than across them. Lastly, the sex-meta has the opposite property, namely that it is expected to result in considerable cryptic relatedness between studies (that is, between male and female subsets) to the degree that it is present in the study overall. Sex was chosen partly because it has been suggested in the literature[13] but also for convenience. In practice, it is not important that the meta-analysis is sex stratified, as sex is random compared to all other variables. Many other designs can result in similarly extreme cryptic relatedness between studies, particularly separate studies of the same population. In addition to standard meta-analysis (labeled as sex-meta and subpop-meta), we include our proposed meta-analysis method for correlated studies (labeled as sex-meta-corr and subpop-meta-corr).

We ran 20 replicates of simulated genotypes with both binary and quantitative traits across the 4 scenarios (Figure 3) and evaluated the various GWAS methods just described. Simulation 1 has population structure but no family structure and is treated as the baseline against which the effects of cryptic relatedness are compared, as the rest contain family structure. In simulation 1, subpop-meta represents common trans-ethnic meta-analyses, whereas sex-meta is a meta-analysis of individually multiethnic studies. Simulation 2 adds 30 generations of family structure only within populations. Subpop-meta is another common design for studies of each of a few distant global populations, so cryptic relatedness is adequately modeled within studies, while no cryptic relatedness exists between studies. Simulation 3 simulates admixture across populations, but family structure remains relatively localized, so it is expected to continue to have a larger effect for sex-meta versus subpop-meta. Lastly, simulation 4 is like simulation 3 without the population structure, for another comparison between the two effects. Simulation 4 has artificial subpopulations constructed by grouping individuals consistent with their family structure, which again yields minimal family structure between studies for subpop-meta compared to sex-meta.

In simulations with family relatedness (simulations 2, 3, and 4), we see both severe inflation ($\lambda > 1.05$ and $\text{SRMSD}_p > 0.01$) and reduced AUC_{PR} for sex-meta compared to subpop-meta and joint analysis (see Figures 4 and S3 for quantitative and binary traits, respectively). When family relatedness exists across subpopulations (simulations 3 and 4), we observe higher inflation in sex-meta compared to when family relatedness exists only within subpopulation (simulation 2). In contrast, sex-meta-corr and subpop-meta-corr are well calibrated in all cases. However, sex-meta-corr does not improve the AUC_{PR} of sex-meta and remains below that of joint analysis when there is confounding.

Remarkably, unlike family structure, differences in ancestry (population structure) between studies appear to have a negligible effect on inflation and calibrated power upon standard meta-analysis, particularly for simulations 1–3 where it would have caused subpop-meta to differ more from joint analysis. Instead, joint is only slightly more calibrated than subpop-meta in binary traits, though the reverse holds in quantitative traits (both are considered effectively calibrated in all of these cases), and there is no clear difference in calibrated power between them except for one case: simulation 1 for quantitative traits only, where joint substantially outperforms subpop-meta. There is also no clear difference in performance between cases where subpopulations are entirely within studies (subpop-meta simulations 1–2) or when several subpopulations are present within each study so there is structure but not relatedness across studies (sex-meta for simulation 1) and when there is admixture so ancestries are mostly but not exclusively within single studies (subpop-meta simulation 3), compared to their joint counterparts or when there is no population structure (subpop-meta simulation 4). Subpop-meta-corr substantially increases AUC_{PR} compared to subpop-meta, in some cases exceeding that of joint. We considered whether correlation between studies explains the improved performance of subpop-meta-corr, but our estimates instead show that the subpop-meta-corr studies are roughly independent, whereas sex-meta-corr studies are predictably correlated (Figure S4). Therefore, we suspect the lower performance of subpop-meta is due to some unknown implementation detail in METAL, since otherwise both meta-analysis methods are expected to give similar results.

We also noticed that subpop-meta-corr often outperformed joint in terms of AUC_{PR}, which we confirmed is a small but statistically significant effect in many of our simulation 1–2 evaluations but never in simulations 3–4 (Table S3). Our best explanation is that, since subpop-meta-corr has additional degrees of freedom (because

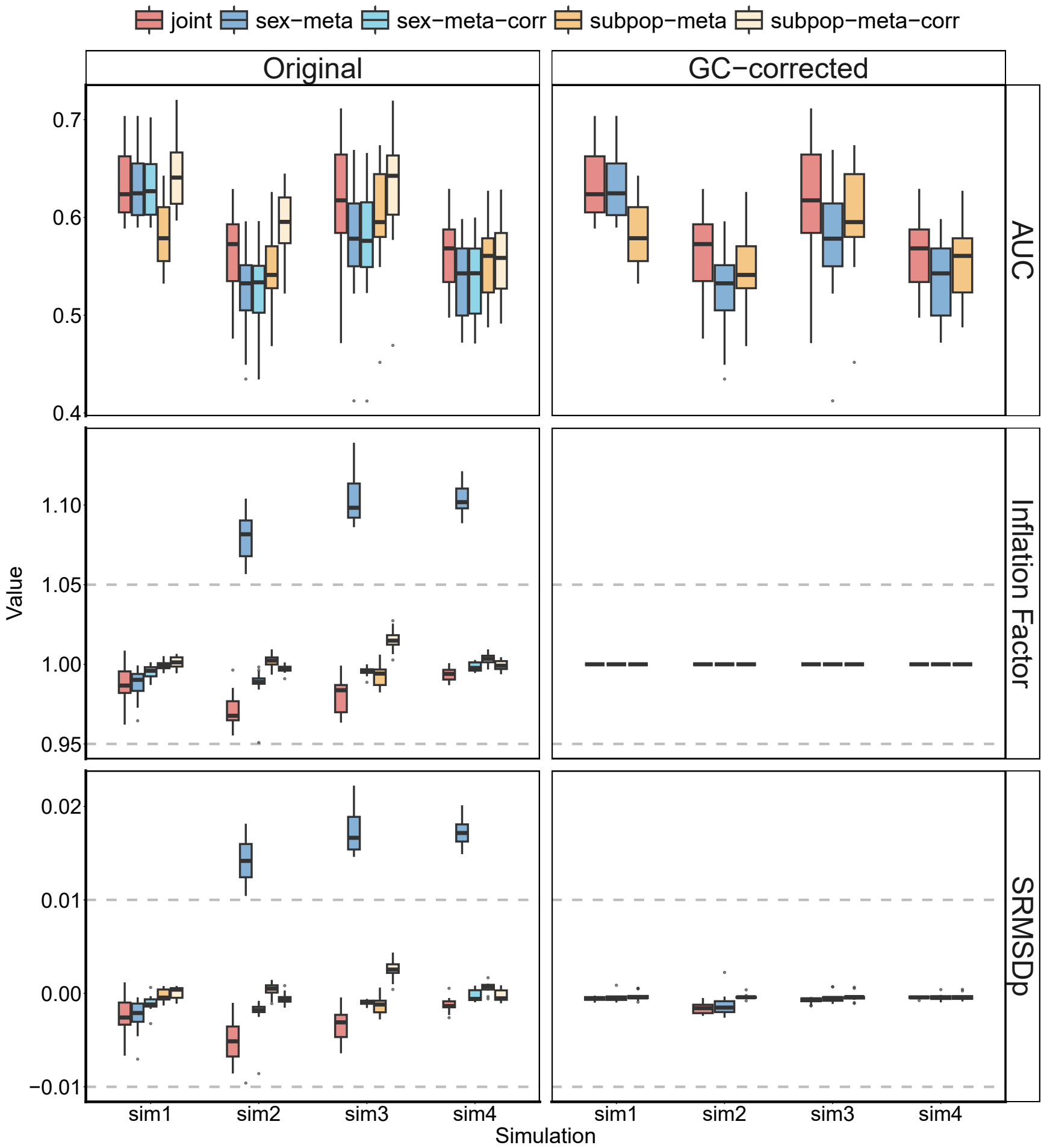

Figure 4. Simulations confirm cryptic relatedness between studies results in considerable inflation upon standard meta-analysis

20 replicates of quantitative traits were simulated here. Simulations (x axis) have different presence and arrangement of population and family structure. AUC_{PR} reflects calibrated power (higher is better), while both inflation factor (1 is best, > 1.05 is inflated) and $SRMSD_p$ (0 is best, > 0.01 is inflated) measure null p value calibration, the latter more strictly (see methods). For simulations with 30 generations of family relatedness (simulations [sim] 2, 3, and 4), sex-meta suffered high inflation and $SRMSD_p$ and lower AUC_{PR} compared to subpop-meta and joint analyses, while sex-meta-corr and subpop-meta-corr are calibrated. GC improves inflation factor and $SRMSD_p$ but does not alter the loss of calibrated power (AUC_{PR}) due to confounding.

each study fits its own intercept), in those cases where studies align perfectly with discrete population structures (simulations 1–2), the meta-analyzed version can better model population structure compared to the random effect version employed by joint. Nevertheless, simulations 1–2 are highly artificial in this regard, and in real data with more heterogeneous subpopulations, we may see results more akin to simulation 3, where joint tended to perform about the same if not better than subpop-meta-corr.

We also compared the default median-based method for estimating study covariance, which has been used so far, to the mean-based method. Both methods had broadly comparable performance across studies and metrics (Figure S5). AUC_{PR} values are practically indistinguishable, and the other measures indicate sufficient calibration in all cases, though inflation is slightly but consistently greater for the mean-based method in binary traits. We further considered more polygenic traits by increasing the number of causal variants to 5,000, which is 1% of 500,000 total loci. We observe the same pattern across all simulations and for both binary and quantitative traits (Figures S6 and S7). Predictably, such an increase in causal variants results in large reductions in AUC_{PR} overall. Nevertheless, median- and mean-based estimators yield comparable AUC_{PR}, inflation factor, and $SRMSD_p$, with mean-based estimation again producing slightly higher inflation for binary traits (Figure S8). Our simulations do not have LD, so each causal variant contributes a signal only at its own locus. Thus, even at higher polygenicity, the additional inflation observable in our framework is limited compared to a real-data analysis at comparable causal density. After considering these limitations, the median-based method remains the best choice moving forward.

Next, we consider the GC adjustment of standard meta-analysis. Note that our proposed method's covariance modeling performs an adjustment similar but less aggressive than GC, so additional adjustment is not needed. By construction, GC always results in perfect inflation factors of one (Figures 4 and S3). For that reason, here in particular we relied on the stricter $SRMSD_p$ statistics to determine if GC actually results in improved null p value calibration. Remarkably, GC adjustment consistently resulted in $|SRMSD_p| < 0.01$, which is considered calibrated,[3] for all approaches, including sex-meta that was highly inflated before the adjustment. Nevertheless, AUC_{PR} is unaffected by GC because GC does not rerank SNPs, while AUC_{PR} is a function of only SNP ranks and their true classes (causal or null). Overall, we find that in these scenarios, GC successfully corrects the type 1 error rate control that otherwise results from inflation, but it does not correct the loss of calibrated power due to confounding by cryptic relatedness.

Lastly, to show that inflation increases with sample size, we extended simulation 4 (single population generated for 30 generations) to 10,000 individuals and drew a series of smaller subsets from it (Figure 5). Sex-meta shows

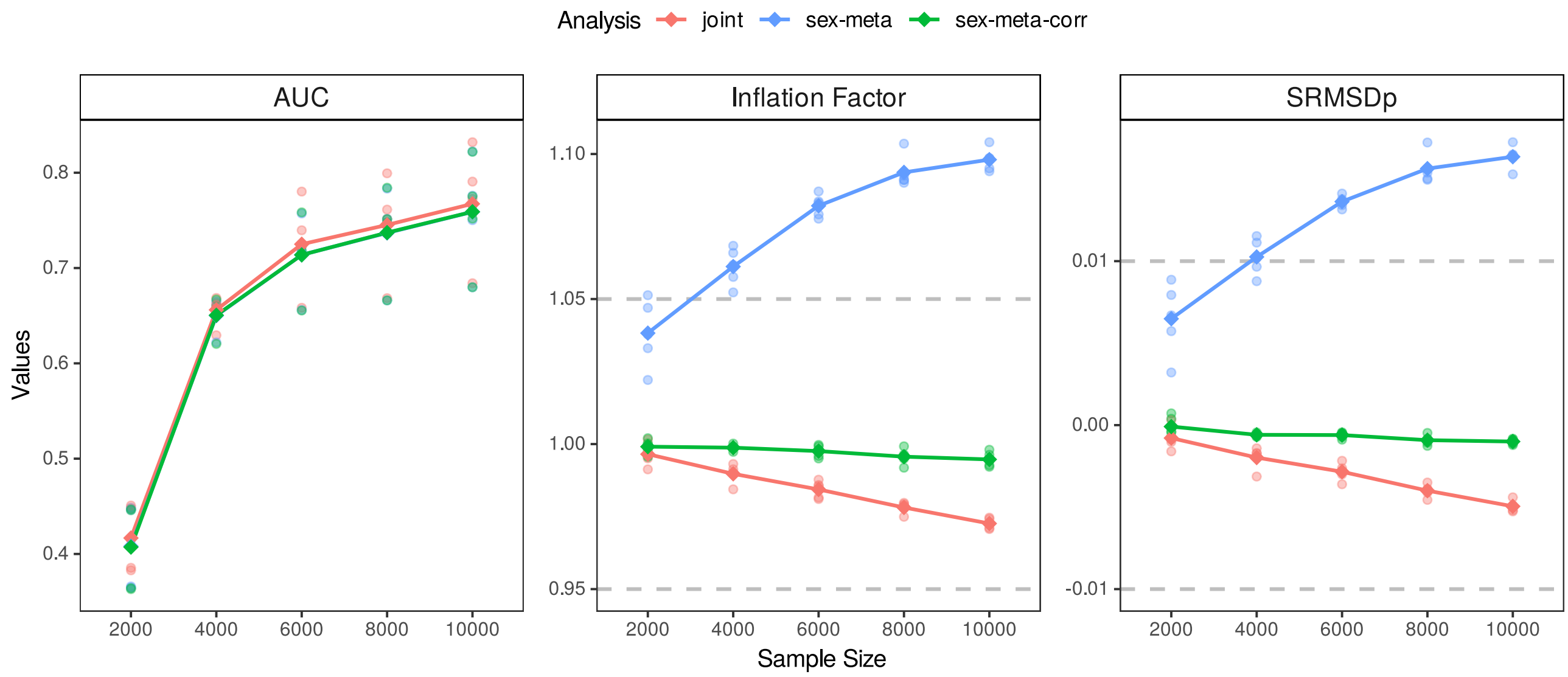

Figure 5. Effect of sample size on joint and sex-stratified meta-analysis
Mean values across 5 replicates using a quantitative trait LMM tested on simulated data (single population of 30 generations) across varying sample sizes. Points show individual replicate values. Inflation factor and $SRMSD_p$ remain well calibrated under joint analysis and sex-meta-corr but increase with sample size for sex-meta. AUC_{PR} improves with sample size comparably for all methods (sex-meta and sex-meta-corr curves overlap).

increased inflation with increasing sample size, exceeding $\lambda > 1.05$ for $n \geq 4{,}000$, consistent with our theoretical prediction. Our proposed sex-meta-corr effectively eliminates inflation at all sample sizes, maintaining calibration better than even joint analysis, consistent with our method correcting for the input deflation that appears to be present here as sample sizes increase. However, in all cases, joint analysis has the highest AUC_{PR} by a small margin. Note that because the starting population here is larger than for the earlier simulation 4 ($n = 3{,}000$), there are proportionally fewer closer relatives (Figure S9), resulting in slightly lower inflation values than in our earlier results (Figures 4 and S3).

Inflation in meta-analyses of real data

We analyze two datasets with real genotypes and phenotypes to determine whether the effects and magnitudes observed in our simulations are also present in practice. However, since true causal variants are generally unknown for real phenotypes, it is not possible to calculate AUC_{PR} and $SRMSD_p$ in these cases, so we focus on inflation factors and quantile-quantile (QQ) plots. We focus on joint and sex-stratified meta-analyses: standard (sex-meta) and our method for correlated studies (sex-corr).

First, we considered T2D-GENES SAMAFS,[36] a study that is large for a family study but small compared to population studies ($n = 914$ individuals). Since there will be considerable family relatedness between the male and female subsets, this dataset clearly demonstrates the inflation in sex-meta that we already illustrated with our simulations. All 21 traits exhibited severe inflation factors for sex-meta, with values exceeding the standard 1.05 threshold except for cystatin C ($\lambda = 1.042$; Figure 6). In contrast, both joint analysis and sex-meta-corr were well calibrated ($0.95 < \lambda < 1.05$), although two traits in the joint analysis approached the thresholds: height ($\lambda = 0.952$) and cholesterol ($\lambda = 1.047$).

Next, we considered HCHS/SOL, a large population study of Hispanic individuals ($n = 11{,}721$) that did not specifically sample close individuals[37] but nevertheless likely contains cryptic relatedness, as do most large studies. Only one of the 33 quantitative traits tested, height, exhibits a high inflation factor ($\lambda = 1.065$) with sex-meta. Nevertheless, calibration is exceptional under joint analysis, and all but one trait (iron blood concentration) show a higher inflation factor in sex-meta than in joint analysis (Figure 6). Sex-meta-corr further reduces inflation factors compared to joint analysis across all traits. Thus, while the effect of cryptic relatedness is less severe in this population study than in SAMAFS, it is still measurable and highly consistent.

We considered whether using LOCO changes our conclusions on real datasets with LD, as there is a possibility that proximal contamination is exacerbated by LD and reduces power.[38–40] LOCO increases inflation factors for both joint and standard sex-meta (Figure S10). Sex-meta-corr is mostly unaffected by this increase in inflation due to LOCO, with points clustering near $\lambda = 1.0$ in both datasets regardless of LOCO status. In SAMAFS, sex-meta still has greater inflation factors than the joint analysis when both are performed using LOCO; however, in HCHS/SOL, LOCO erases the difference in inflation between joint and sex-meta (Figure S11). Sex-meta-corr yields inflation factors close to 1.0 and near the diagonal for both datasets. Since real traits do not allow us to easily disentangle increases in inflation due to higher type I error

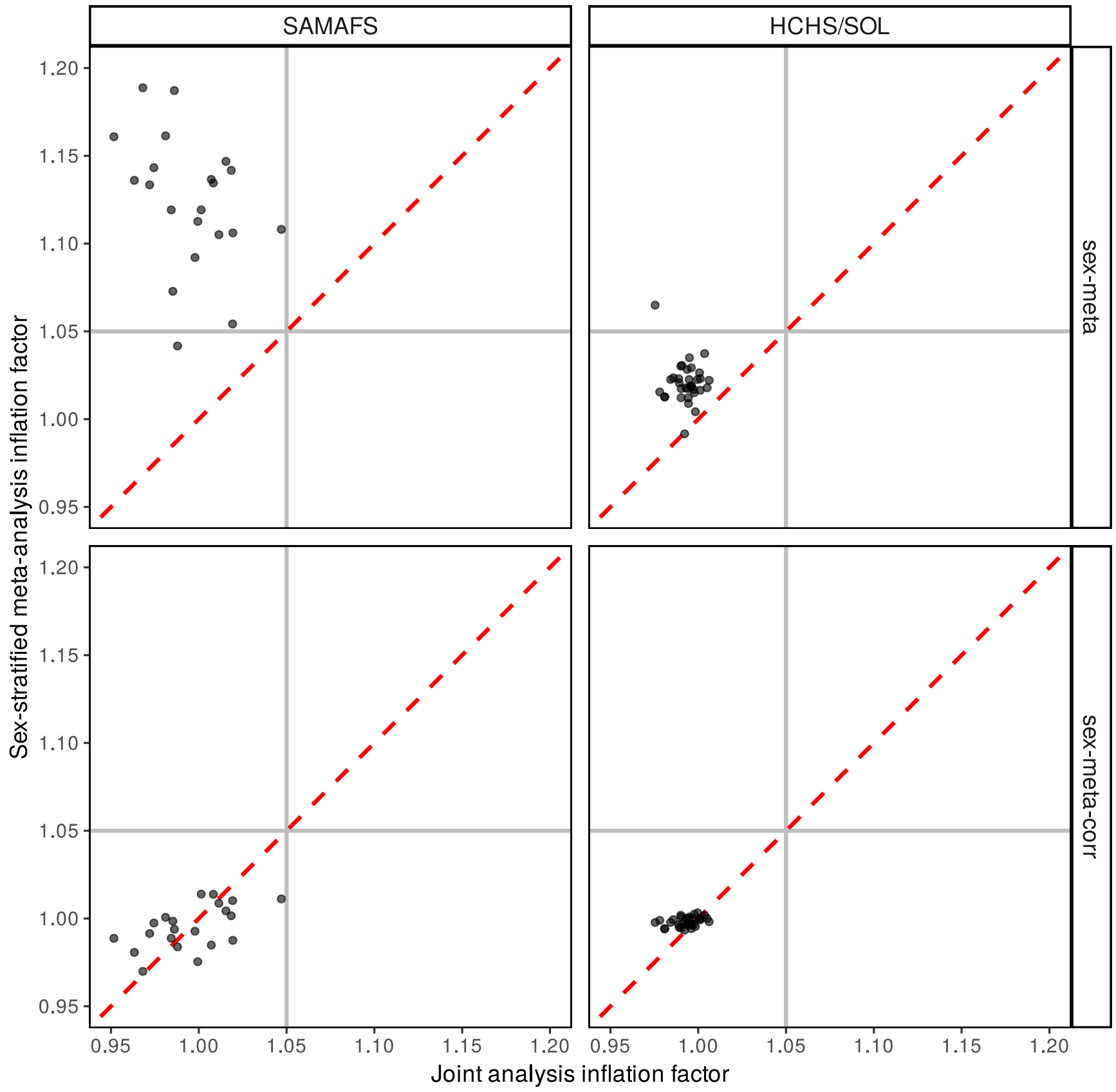

Figure 6. Inflation is greater in sex-stratified standard meta-analysis of real genotypes and phenotypes
Inflation factors from joint analysis (x axis) are compared to those from sex-stratified meta-analysis (y axis) across traits in the T2D-GENES SAMAFS family study (20 quantitative traits and 1 binary trait) and the HCHS/SOL population study (33 quantitative traits). Under standard meta-analysis (sex-meta), nearly all traits fall above the $y = x$ diagonal, indicating greater inflation than in the joint analysis, particularly in SAMAFS, where all but one trait exceed the $\lambda = 1.05$ threshold. Our method (sex-meta-corr) has inflation factors that fall even closer to 1.0 than the joint analysis in both datasets.

rates from increases in polygenic power, we next simulated traits with the real genotypes. In these simulations, where true causal variants are known, we can calculate AUC_{PR} and $SRMSD_p$ to distinguish the two explanations for increased inflation. In both datasets, we recapitulate the behaviors observed for the real traits with the simulated traits concerning LOCO and find that AUC_{PR} is either the same or reduced while $SRMSD_p$ increases drastically when LOCO is used (Figure S12). Sex-meta-corr inflation factors and $SRMSD_p$ remain well calibrated in all cases, but AUC_{PR} is not again improved relative to sex-meta and remains lower than for joint analysis. Thus, at least in these small datasets, we find that LOCO severely increases type I error rates and reduces calibrated power, which confounds the comparison of interest between joint and standard sex-meta.

Lastly, we consider the effect of GC adjustment on inflated standard meta-analysis p values of the real phenotypes. As explained earlier, it is not possible to evaluate GC using inflation factors λ, as GC always results in $\lambda = 1$, while that does not guarantee adequate type I error control at other quantiles. Absent more precise measures such as $SRMSD_p$, which is only available for simulated datasets with known causal variants, we performed a more holistic assessment by visualizing calibration across quantiles using QQ plots. For simplicity, we focused on the top three most inflated traits per dataset, which will have larger adjustments performed by GC and thus a greater potential for overcorrection. We again found good performance for GC adjustment of meta-analyzed p values, which are visually close to the null expectation across a large portion of the curves, especially on the lower end as desired, and overlapping joint and sex-meta-corr (Figure 7). In contrast, inflation for the SAMAFS traits is visually noticeable, whereas HCHS/SOL has negligible inflation, as determined earlier by our calculated inflation factors. If GC had overcorrected or otherwise failed, since GC forces the median to go through the diagonal (null expectation), we would expect at least a portion of the QQ curve to fall below the diagonal, but this did not occur in the real data, although sex-meta-corr did overcorrect slightly for BMI in SAMAFS. To confirm and visualize these hypothetical cases of GC overcorrection, we simulated p values under a high polygenicity and high power assumptions, namely that half of the test statistics are alternative from a non-central χ^2 distribution. Indeed, as power increases, so does the GC overcorrection, resulting in large portions of the QQ curve well below the expected diagonal line (Figure S13); again, this was not observed in the real data, so GC adjustment appears to result in acceptable calibration. Therefore, as observed earlier for the simulated data, GC appears to be an adequate solution to correct inflation, but not loss of calibrated power, in meta-analysis due to cryptic relatedness, at least in small studies. In most cases, our method for correlated meta-analysis achieves similar performance using a more principled approach than GC.

Discussion

Meta-analysis is a well-established GWAS approach that aggregates data from multiple smaller studies to achieve a larger effective sample size and utilizes summary

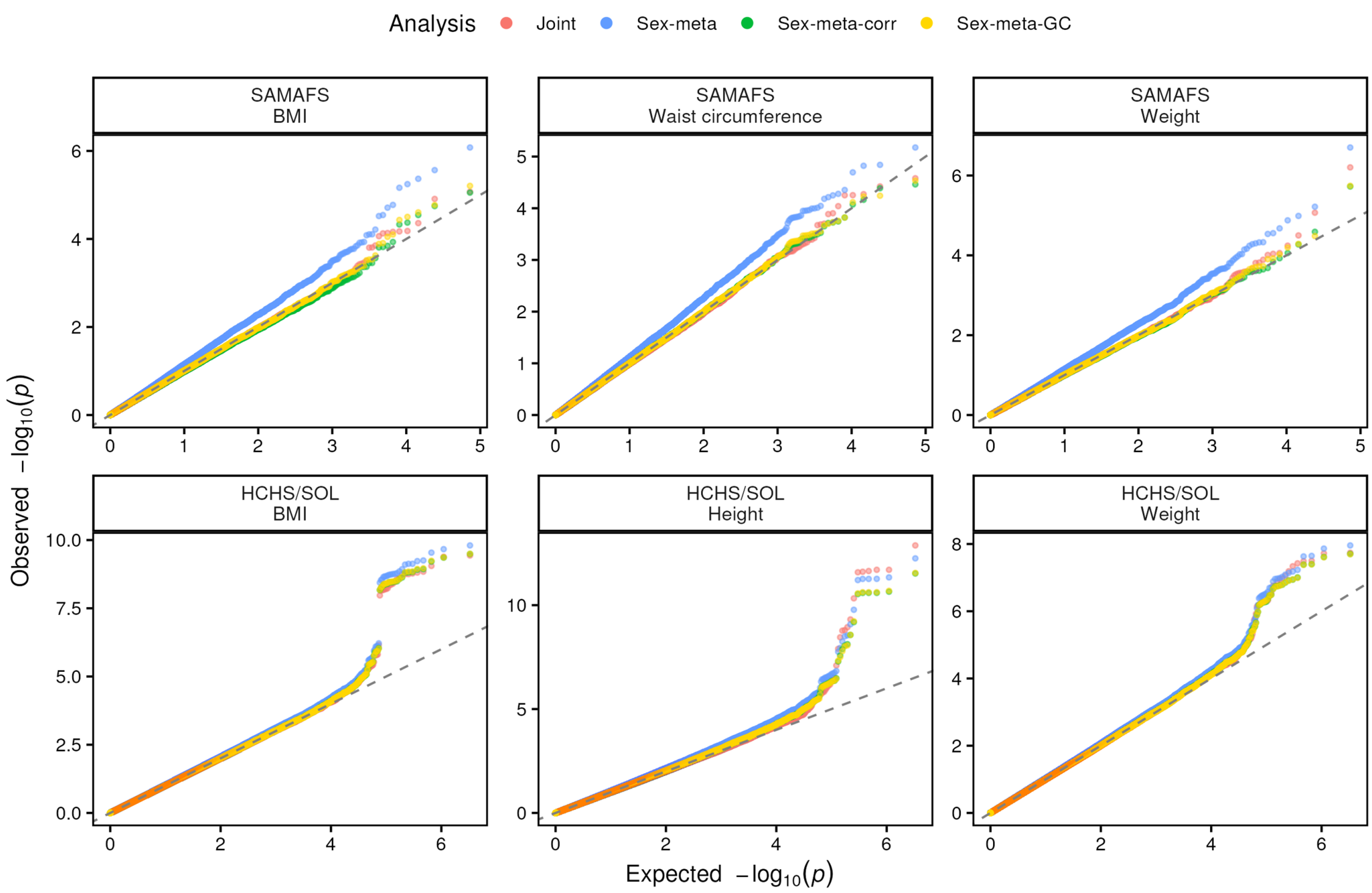

Figure 7. Quantile-quantile plots for most inflated traits upon standard meta-analysis
Top three traits per dataset were ranked by sex-meta inflation factor. The sex-meta curve rises earlier above the rest and above the null expectation (diagonal $y = x$ line), reflecting inflation (higher in SAMAFS), while joint, sex-meta-GC, and sex-meta-corr overlap the diagonal and each other across much of the range, reflecting comparable calibration and power. However, the joint analysis curve stands out among the calibrated methods for rising higher at greater $-\log 10(p)$ values, reflecting its higher calibrated power. There is greater power overall in HCHS/SOL compared to SAMAFS due to the difference in sample size. Only one method appears to overcorrect (curve dips slightly below diagonal) in one trait: sex-meta-corr in SAMAFS BMI.

statistics instead of individual-level genotype data to improve power and accessibility. The main drawback is that the independence assumption between studies is not always met, and the effect of cryptic relatedness on GWAS meta-analysis was unclear before our work. Using simulations and real data, we confirmed that the presence of family structure between studies causes inflation in standard meta-analysis, which is severe for family studies at low sample sizes and observed to have a smaller effect in population studies with up to $n \approx 10{,}000$ individuals. Both binary and quantitative trait GWAS models show strong inflation in sex-stratified standard meta-analysis. Our theory predicts, and simulations confirm, that if there is cryptic relatedness between studies, increasing sample size also increases confounding (Figures 2 and 5). Even at low sampling fractions of a large population, a substantial fraction of sampled individuals are expected to share at least one second cousin within the sample.[41] Thus, we believe that accounting for cryptic relatedness between large cohort studies, such as biobanks, will be a crucial challenge that existing meta-analysis approaches are ill-equipped for. Here, we proposed a meta-analysis method that estimates and models covariance between the studies, resulting in calibrated statistics and addressing this key shortcoming of standard meta-analysis. However, meta-analysis suffers from reduced calibrated power under confounding, even when study covariance is modeled, which we believe is a general shortcoming of meta-analysis that is only overcome by performing joint GWAS.

Prediction of the amount of inflation due to cryptic relatedness can be attempted given some of our results. One set of simulations suggested that the variance of kinship values between studies, which drives inflation, is constant for a given population and particularly does not depend on sample size (Figure S1). Thus, we could use empirical inflation at one sample size to roughly predict inflation at larger sample sizes for the same population and the same trait. In particular, excess inflation $\lambda - 1$ is predicted to grow proportionally to sample size, under the simplistic assumptions that studies have equal sizes and standard errors and are drawn from the same otherwise unstructured population. Thus, since excess inflation was around 0.025 for many traits in HCHS/SOL (Figure 6) and the study sizes were approximately $n = 5{,}000$, the approximation tells us to expect excess inflation of 2.5 ($\lambda = 3.5$) if we increase sample sizes 100-fold, which is the size of the UK Biobank at half a million individuals. The extent to which such a number can be extrapolated to populations other than HCHS/SOL depends on comparable population history, particularly effective population size, and greater inflation is expected for smaller effective population sizes. On the other hand, a more direct

simulation found a sub-linear dependence between excess inflation and sample size (Figure 5). Therefore, despite some promising results, precise predictions of inflation due to cryptic relatedness in meta-analysis of large studies remain elusive.

Although cryptic relatedness between studies causes inflation in standard meta-analysis, population structure (ancestry differences) between studies does not have the same effect in our simulations. Our hypothesis is that these cases behave differently due to the dimensionality of their structures. Population structure is low dimensional because it is widely shared, so modeling it within studies (via the intercept for single ancestries or via PCA for more complex admixture scenarios) suffices to completely remove the effect between studies, resulting in test statistics that are independent between studies. On the other hand, cryptic (family) relatedness is high dimensional and not broadly shared.[3] In that case, since the relationship between a pair of individuals from different studies is by hypothesis absent within studies, it is not modeled by them and remains a confounder upon meta-analysis. Thus, our results suggest that meta-analyses of studies from different populations, especially distant populations with less migration between them, are likely much less affected by cryptic relatedness and thus less likely to experience inflation, even in the presence of ancestry differences, absent other causes.

Recent work that suggested applying sex-meta to correct for participation bias did not consider relatedness between individuals of opposite sex drawn from the same population.[13] Although we suggested that a joint analysis is preferable to a sex-meta to avoid inflation due to cryptic relatedness, we have not addressed the primary concern of that work, which is that there is a potentially sex-specific effect in the observed association, which is due to sex-differential participation bias that we wish to ignore.[42] Meta-analysis in this case allows each sex to have its own intercept as well as its own genetic effect coefficient, so it successfully makes sex-specific associations vanish. A standard joint analysis allows for a sex-specific intercept by including sex as a fixed covariate, but this does not allow for sex-specific genetic effects unless we introduce an interaction term between genotype and sex and condition the overall association (the genetic effect shared between men and women) by the sex-specific effects. The most popular scalable LMM packages do not allow such interactions between genotypes and covariates, but it is possible to carry out such analyses in custom LMM implementations, although it would be preferable for highly optimized versions to be available.

A recent high-profile example, that height polygenic risk scores from the GIANT consortium show evidence of confounding compared to estimates from the UK Biobank, could be explained by cryptic relatedness between the studies meta-analyzed in GIANT, combined with the large sample sizes involved. GIANT performed a meta-analysis of 79 GWASs of European ancestry, totaling 252,288 individuals, and observed a large inflation factor of 1.94, even though the input studies were corrected using GC.[43] Unfortunately, joint analysis of the GIANT data was not performed, precluding a conclusive argument. However, two different groups used a joint GWAS of the UK Biobank, and their in-depth analysis showed that GIANT suffered from confounding correlated to population structure.[44,45] Following an analogy to our work, we would expect the UK Biobank studies to be well calibrated, but unfortunately, there is inflation there too, and its interpretation is complicated. Because these works did not report inflation factors, we downloaded their height summary statistics and calculated p value inflation using the formula given in Yao and Ochoa.[3] We obtained $\lambda = 1.92$ for GIANT and $\lambda = 1.81$ for UK Biobank under linear regression with PCs (336,474 individuals) and $\lambda = 2.09$ with BOLT-LMM (458,303 individuals).[44] Furthermore, the original BOLT-LMM analysis identified height as the trait with the greatest inflation and used LD score regression to estimate an intercept of 1.47.[46] In principle, this intercept isolates inflation due to confounding only, excluding the polygenic effect,[47] though other deviations from the idealized model appear to result in such inflation at very large sample sizes. This suggests that there is inflation due to both polygenicity and misspecification in the UK Biobank data, though it is unclear to what extent the high inflation in GIANT has the same explanation. Overall, since GIANT meta-analyzed numerous studies from closely related populations in Europe and other European-majority regions, we believe that it is particularly susceptible to confounding due to cryptic relatedness between these studies, although at present it is not possible to be conclusive without a joint GWAS of GIANT or application of our method that models covariance between studies.

We incidentally identified poor behavior when using LOCO in our LMM associations of the two real genotype datasets, HCHS/SOL and SAMAFS, both of which consist of admixed populations, and the latter also has considerable family structure.[36,37] LOCO was developed to prevent the association signal of the locus being tested from being partially conditioned upon by including it and its LD neighbors in the GRM, which could reduce power.[38] However, since it is not feasible to recalculate the GRM at every locus to exclude its neighborhood, LOCO additionally excludes the whole chromosome of the test locus, a trade-off that requires only 22 GRMs (one for each excluded autosome).[39,40] Therefore, we hypothesize that LOCO may insufficiently control for population structure by erring too much in the opposite direction: it excludes from the GRM too many causal loci that are not in LD with the test locus, leading to inadequate modeling of the polygenic random effect, which increases type I error. Furthermore, inflation due to using LOCO increases with greater genetic structure; this also occurs when relatedness is not modeled more broadly, for example by not using LMMs.[3] Thus, we expect that this shortcoming of LOCO was not

identified originally because it was evaluated in relatively unstructured data.[38–40] Consistent with our observations and hypothesis, recent work focused on simulated admixture using real haplotypes from 1000 Genomes also concluded that LOCO results in FPs not found without LOCO, whereas using or not using LOCO performs similarly in the unadmixed cohorts.[48] Overall, evaluations of LOCO have been limited, and more research is needed to determine its behavior in genetically complex cohorts.

There are four potential solutions to the problem of between-study cryptic relatedness, should undesirable inflation be evident: (1) applying joint analysis with a method that models cryptic relatedness such as LMMs, (2) using our proposed meta-analysis method that models covariance between studies, (3) pruning of cryptic relatives between studies for standard meta-analysis, or (4) correcting the inflation after meta-analysis with GC or LD score regression. The first option is preferable, as only complete modeling of cryptic relatedness without exclusion of individuals maximizes power compared to the other options. Besides traditional joint LMM analyses, federated GWASs are another alternative to traditional meta-analysis that utilizes full genomic datasets instead of summary statistics for conducting collaborative GWASs across different institutes without jeopardizing patient privacy. Our work may motivate further developments of such federated approaches or improvements in responsible data-sharing policies to facilitate joint analyses. The second option, our proposed method, is the most principled way to perform meta-analysis under covariance, which solves the inflation problems of standard meta-analysis but does not reliably improve calibrated power compared to joint analysis. Moreover, although our method performed very well in our evaluations, it is possible that study covariance is overestimated in studies with highly polygenic traits and high power, cases where overcorrection by GC is also possible. The MTAG (multi-trait analysis of GWAS) method,[49] though developed for different traits, somewhat overlaps with ours when the trait is the same across studies; the most interesting difference is that their study covariance matrix is estimated using bivariate LD score regression instead of our median and mean approaches, an alternative that deserves further study. For the third option, methods such as KING, GERMLINE, TRUFFLE, or IBIS[50–53] can infer the degrees of relatedness for filtering as a quality control step. Privacy-aware federated approaches for identifying relatives between studies are particularly useful in cases where individual-level data cannot be shared, as long as researchers on both sides are willing to collaborate.[14–16] However, removing increasingly distant relatives can result in large sample size reductions: in datasets such as Human Origins, HGDP, and 1000 Genomes, removing 4th-degree relatives reduces sample sizes by 5%–10%, yet confounding due to cryptic relatedness remained for non-LMM methods, showing that the larger number of very distant relatives poses significant problems when they are not modeled in GWAS.[3] We are not aware of any scenarios where standard meta-analysis after removing relatives is a better choice than meta-analysis modeling covariance between studies. Lastly, for existing standard meta-analyses that cannot feasibly be rerun, GC can adjust inflated test statistics, which in both our simulated and real data results in surprisingly accurate type I error control. However, GC does not improve the calibrated power to detect true signals compared to the joint analysis (Figures 4 and S3), which makes sense since GC does not directly model cryptic relatedness. GC has historically performed poorly for correcting type I error rates,[6,7,25–27] which is an important reason to have reservations despite the positive results we present here. Furthermore, highly polygenic traits, such as height, can exhibit increased genomic inflation factor when the GWAS has high power, in the absence of confounding,[54] so GC could overcorrect p values in such cases. We confirmed such overcorrection by GC in simulations with extreme amounts of polygenicity and power, which is evident in QQ plots (Figure S13), which can be used to diagnose this potential problem. Perhaps a more conservative GC-like adjustment could instead use the intercept of LD score regression, which captures inflation due to confounding while excluding the polygenic effect.[47] However, LD score regression assumes homogeneous populations,[47] and at very large sample sizes, it still results in inflation due to small deviations from theory,[46] so this potentially promising application would require careful evaluation. Ultimately, to prevent these problems, we recommend avoiding meta-analysis of studies that are drawn from the same genetic populations.

Data and code availability

- Our meta-analysis method that estimates and models covariance between studies is implemented in the R package `metalcor`, available on the CRAN and on GitHub at https://github.com/OchoaLab/metalcor.
- The data and analysis code generated during this study are available on GitHub at https://github.com/OchoaLab/meta-gwas-cryptic-inflation.
- The HCHS/SOL[37] is available on dbGaP (dbGaP: phs000810.v2.p2). The SAMAFS[36] is available on dbGaP (dbGaP: phs000847.v2.p1).

Acknowledgments

This work was funded in part by the Duke University School of Medicine Whitehead Scholars Program, a gift from the Whitehead Charitable Foundation. Thanks to Zhuoran Hou for performing quality control of SAMAFS and HCHS/SOL datasets, as well as selecting the traits we analyzed in this work. Please see the supplemental information for acknowledgments regarding the SAMAFS and HCHS/SOL studies.

Declaration of interests

The authors declare no competing interests.

Web resources

dbGaP, https://dbgap.ncbi.nlm.nih.gov/home/

metalcor, https://github.com/OchoaLab/metalcor

Meta-GWAS Cryptic Inflation Project Repository, https://github.com/OchoaLab/meta-gwas-cryptic-inflation

Appendix A: The covariance between the Z scores of two GWASs with relatedness between them

General formula under joint LMM

Here, we derive the form of $\text{Cov}(z_{ij}, z_{ik})$ under the assumptions of the joint LMM of studies j and k, which clearly reveals how relatedness between studies results in within-study Z scores that are correlated across studies. Specifically, we assume the following trait covariance matrix for studies j and k jointly, which is implied by Equation 1, written to clarify its block structure:

$$\text{Cov}\begin{pmatrix} \mathbf{y}_j \\ \mathbf{y}_k \end{pmatrix} = \sigma_y^2 \begin{pmatrix} \mathbf{V}_j & \mathbf{V}_{jk} \\ \mathbf{V}'_{jk} & \mathbf{V}_k \end{pmatrix} = \sigma_y^2 \left(2h^2 \begin{pmatrix} \mathbf{\Phi}_j & \mathbf{\Phi}_{jk} \\ \mathbf{\Phi}'_{jk} & \mathbf{\Phi}_k \end{pmatrix} + (1-h^2) \begin{pmatrix} \mathbf{I}_{n_j} & \mathbf{0}_{n_j \times n_k} \\ \mathbf{0}_{n_k \times n_j} & \mathbf{I}_{n_k} \end{pmatrix} \right),$$

where $\mathbf{V}_{jk}$ and $\mathbf{\Phi}_{jk}$ are $n_j \times n_k$ trait covariance and kinship matrices between the individuals in studies j and k, respectively, $\mathbf{0}_{n_j \times n_k}$ is an $n_j \times n_k$ matrix of zero values, and the rest follows the notation in the main methods. Thus, subsetting to the between-studies block, we obtain the cross-covariance between the trait vectors in two different studies:

$$\text{Cov}\left(\mathbf{y}_j, \mathbf{y}_k\right) = 2\sigma_y^2 h^2 \mathbf{\Phi}_{jk}. \qquad \text{(Equation A1)}$$

The joint LMM assumes that the trait is fundamentally the same across studies, so σ_y^2 and h^2 are the same too. Furthermore, we assume there is no additional covariance due to non-genetic environment that is unmodeled; however, if that were present, it could increase inflation further, so that simplistic assumption does not negate our key conclusion.

Given a known $\mathbf{V}_j$, the standard generalized least-squares solution used by modern LMMs for the genetic effect and its estimated variance for SNP i and study j are

$$\begin{aligned} \mathbf{M}_j &= \mathbf{V}_j^{-1} - \frac{\mathbf{V}_j^{-1}\mathbf{1}_{n_j}\mathbf{1}'_{n_j}\mathbf{V}_j^{-1}}{\mathbf{1}'_{n_j}\mathbf{V}_j^{-1}\mathbf{1}_{n_j}}, \\ \widehat{\beta}_{ij} &= \frac{\mathbf{x}'_{ij}\mathbf{M}_j\mathbf{y}_j}{\mathbf{x}'_{ij}\mathbf{M}_j\mathbf{x}_{ij}}, \\ \widehat{\sigma}^2_{ij} &= \frac{\sigma_y^2}{\mathbf{x}'_{ij}\mathbf{M}_j\mathbf{x}_{ij}}, \end{aligned}$$

where, for notational simplicity, we assumed that no covariates beyond the intercept were present, though the key results hold without this restriction. Note that $\mathbf{M}_j$ is symmetric since $\mathbf{V}_j$ is too. The Z score is therefore

$$\begin{aligned} z_{ij} &= \frac{\widehat{\beta}_{ij}}{\widehat{\sigma}_{ij}} = \mathbf{h}_{ij}\mathbf{y}_j, \\ \mathbf{h}_{ij} &= \frac{\mathbf{x}'_{ij}\mathbf{M}_j}{\sqrt{\sigma_y^2\mathbf{x}'_{ij}\mathbf{M}_j\mathbf{x}_{ij}}}. \end{aligned}$$

Note that $\mathbf{h}_{ij}$ does not depend on the trait, so it is considered fixed for now. Therefore, the covariance between test statistics across studies j and k arises from the covariance between their traits, which is in turn given by the relatedness between studies as stated in Equation A1:

$$\begin{aligned} \text{Cov}(z_{ij}, z_{ik}) &= \text{Cov}\left(\mathbf{h}_{ij}\mathbf{y}_j, \mathbf{h}_{ik}\mathbf{y}_k\right) \\ &= \mathbf{h}_{ij}\text{Cov}\left(\mathbf{y}_j, \mathbf{y}_k\right)\mathbf{h}'_{ik} \\ &= 2\sigma_y^2 h^2 \mathbf{h}_{ij}\mathbf{\Phi}_{jk}\mathbf{h}'_{ik} \\ &= 2h^2 \frac{\mathbf{x}'_{ij}\mathbf{M}_j\mathbf{\Phi}_{jk}\mathbf{M}_k\mathbf{x}_{ik}}{\sqrt{\left(\mathbf{x}'_{ij}\mathbf{M}_j\mathbf{x}_{ij}\right)\left(\mathbf{x}'_{ik}\mathbf{M}_k\mathbf{x}_{ik}\right)}}. \end{aligned}$$

Furthermore, σ_y^2 no longer appears in these formulas, as inflation does not depend on the scale of the trait, but it does depend on the heritability of the trait.

Formula marginalizing genotypes under kinship model

In order to understand the connection between allele frequency, population structure, and inflation, it is further necessary to take expectations of the above covariance over the possible values of the genotype vectors. For this, we consider the kinship model, which is also the motivation behind the LMM.[8] Stated to cover the genotypes of both studies j and k, the kinship model establishes that the mean and covariance structure of these genotypes are given by

$$\begin{aligned} \text{E}\begin{pmatrix} \mathbf{x}_j \\ \mathbf{x}_k \end{pmatrix} &= 2p_i \begin{pmatrix} \mathbf{1}_{n_j} \\ \mathbf{1}_{n_k} \end{pmatrix}, \\ \text{Cov}\begin{pmatrix} \mathbf{x}_j \\ \mathbf{x}_k \end{pmatrix} &= 4p_i(1-p_i)\begin{pmatrix} \mathbf{\Phi}_j & \mathbf{\Phi}_{jk} \\ \mathbf{\Phi}'_{jk} & \mathbf{\Phi}_k \end{pmatrix}. \end{aligned}$$

Using the well-known identity

$$\text{E}[\mathbf{x}'\mathbf{A}\mathbf{y}] = \text{E}[\mathbf{x}]'\mathbf{A}\text{E}[\mathbf{y}] + \text{Tr}(\text{Cov}(\mathbf{x},\mathbf{y})'\mathbf{A}),$$

where $\mathbf{A}$ is fixed, we calculate the expectation over random genotypes of the numerator and the parts of the denominator of the desired covariance:

$$\begin{aligned} \text{E}\left[\mathbf{x}'_{ij}\mathbf{M}_j\mathbf{\Phi}_{jk}\mathbf{M}_k\mathbf{x}_{ik}\right] &= 4p_i(1-p_i)\text{Tr}\left(\mathbf{\Phi}'_{jk}\mathbf{M}_j\mathbf{\Phi}_{jk}\mathbf{M}_k\right), \\ \text{E}\left[\mathbf{x}'_{ij}\mathbf{M}_j\mathbf{x}_{ij}\right] &= 4p_i(1-p_i)\text{Tr}(\mathbf{\Phi}_j\mathbf{M}_j), \end{aligned}$$

where the mean terms vanish because the intercept $\mathbf{1}_{n_j}$ is in the null space of $\mathbf{M}_j$, and the same is true for study k:

$$\mathbf{M}_j\mathbf{1}_{n_j} = \mathbf{V}_j^{-1}\mathbf{1}_{n_j} - \mathbf{V}_j^{-1}\mathbf{1}_{n_j}\frac{\mathbf{1}'_{n_j}\mathbf{V}_j^{-1}\mathbf{1}_{n_j}}{\mathbf{1}'_{n_j}\mathbf{V}_j^{-1}\mathbf{1}_{n_j}} = \mathbf{0}_{n_j}.$$

Thus, assuming we can approximate the expectation of the function of those random variables with the function of their expectations, we get an approximate expectation over genotypes of

$$\text{Cov}(z_{ij}, z_{ik}) \approx 2h^2 \frac{\text{Tr}\left(\mathbf{\Phi}'_{jk}\mathbf{M}_j\mathbf{\Phi}_{jk}\mathbf{M}_k\right)}{\sqrt{\text{Tr}(\mathbf{\Phi}_j\mathbf{M}_j)\text{Tr}(\mathbf{\Phi}_k\mathbf{M}_k)}}. \qquad \text{(Equation A2)}$$

Note that this inflation term therefore does not depend on the allele frequency p_i of the tested SNP, as those factors have canceled out. In fact, the value is the same across all SNPs for the same two studies j and k.

Formula under random kinship distribution

The previous formulas for specific kinship matrices are easier to understand if we treat kinship values themselves as random. To calculate an expectation, we simply need the mean and variance of the distribution of cross-kinship values, and for simplicity, we will assume that different values φ in the cross-kinship matrix are uncorrelated with each other. First note this identity, where the elements of the $m \times n$ matrix $\mathbf{X}$ are independent and identically distributed with variance σ^2, while $\mathbf{A}$ is fixed, then

$$\mathrm{E}[\mathbf{XAX}'] = \mathrm{E}[\mathbf{X}]\mathbf{A}\mathrm{E}[\mathbf{X}]' + \mathbf{I}_m \mathrm{Tr}(\mathbf{A})\sigma^2.$$

Thus, applied to our case, which has $\mathrm{E}\left[\mathbf{\Phi}_{jk}\right] = \mu_\varphi \mathbf{1}_{n_j}\mathbf{1}'_{n_k}$, and variance σ^2_φ, which is appropriate for cryptic relatedness but not population structure, we get

$$\begin{aligned}
\mathrm{E}\left[\mathrm{Tr}\left(\mathbf{\Phi}'_{jk}\mathbf{M}_j\mathbf{\Phi}_{jk}\mathbf{M}_k\right)\right] &= \mathrm{Tr}\left(\mathrm{E}\left[\mathbf{\Phi}'_{jk}\mathbf{M}_j\mathbf{\Phi}_{jk}\right]\mathbf{M}_k\right) \\
&= \mathrm{Tr}\left(\mu_\varphi \mathbf{1}_{n_k}\mathbf{1}'_{n_j}\mathbf{M}_j\mu_\varphi \mathbf{1}_{n_j}\mathbf{1}'_{n_k}\mathbf{M}_k\right) \\
&\quad + \mathrm{Tr}\left(\mathrm{Tr}(\mathbf{M}_j)\sigma^2_\varphi \mathbf{M}_k\right) \\
&= \sigma^2_\varphi \mathrm{Tr}(\mathbf{M}_j)\mathrm{Tr}(\mathbf{M}_k),
\end{aligned}$$

where the mean terms vanish again as before.

The kinship within studies is more challenging to model as random because kinship is also estimated for the LMM, so it appears in the $\mathbf{M}_j$ terms as well (in contrast, kinship between studies does not appear in within-study LMM parameters). Nevertheless, since $\mathbf{V}_j\mathbf{V}_j^{-1} = \mathbf{I}_{n_j}$, and expanding the uninverted $\mathbf{V}_j$, we get a way to rewrite one product involving $\mathbf{\Phi}_j$ into terms that do not involve this kinship except through $\mathbf{V}_j^{-1}$:

$$\begin{aligned}
\mathbf{I}_{n_j} &= \left(2h^2\mathbf{\Phi}_j + (1-h^2)\mathbf{I}_{n_j}\right)\mathbf{V}_j^{-1} \\
&= 2h^2\mathbf{\Phi}_j\mathbf{V}_j^{-1} + (1-h^2)\mathbf{V}_j^{-1}, \\
\mathbf{\Phi}_j\mathbf{V}_j^{-1} &= \frac{1}{2h^2}\left(\mathbf{I}_{n_j} - (1-h^2)\mathbf{V}_j^{-1}\right), \\
\mathbf{\Phi}_j\mathbf{M}_j &= \mathbf{\Phi}_j\mathbf{V}_j^{-1}\left(\mathbf{I}_{n_j} - \frac{\mathbf{1}_{n_j}\mathbf{1}'_{n_j}\mathbf{V}_j^{-1}}{\mathbf{1}'_{n_j}\mathbf{V}_j^{-1}\mathbf{1}_{n_j}}\right) \\
&= \frac{1}{2h^2}\left(\mathbf{I}_{n_j} - (1-h^2)\mathbf{V}_j^{-1}\right)\left(\mathbf{I}_{n_j} - \frac{\mathbf{1}_{n_j}\mathbf{1}'_{n_j}\mathbf{V}_j^{-1}}{\mathbf{1}'_{n_j}\mathbf{V}_j^{-1}\mathbf{1}_{n_j}}\right) \\
&= \frac{1}{2h^2}\left(\mathbf{I}_{n_j} - \frac{\mathbf{1}_{n_j}\mathbf{1}'_{n_j}\mathbf{V}_j^{-1}}{\mathbf{1}'_{n_j}\mathbf{V}_j^{-1}\mathbf{1}_{n_j}} - (1-h^2)\mathbf{M}_j\right), \\
\mathrm{Tr}(\mathbf{\Phi}_j\mathbf{M}_j) &= \frac{1}{2h^2}\mathrm{Tr}\left(\mathbf{I}_{n_j} - \frac{\mathbf{1}_{n_j}\mathbf{1}'_{n_j}\mathbf{V}_j^{-1}}{\mathbf{1}'_{n_j}\mathbf{V}_j^{-1}\mathbf{1}_{n_j}} - (1-h^2)\mathbf{M}_j\right) \\
&= \frac{1}{2h^2}\left(\mathrm{Tr}\left(\mathbf{I}_{n_j}\right) - \frac{\mathrm{Tr}\left(\mathbf{1}_{n_j}\mathbf{1}'_{n_j}\mathbf{V}_j^{-1}\right)}{\mathbf{1}'_{n_j}\mathbf{V}_j^{-1}\mathbf{1}_{n_j}} - (1-h^2)\mathrm{Tr}(\mathbf{M}_j)\right) \\
&= \frac{n_j - 1 - (1-h^2)\mathrm{Tr}(\mathbf{M}_j)}{2h^2}.
\end{aligned}$$

The above assumes $h^2 \neq 0$, which is also necessary to have inflation. Altogether, the Z score covariance becomes

$$\begin{aligned}
\mathrm{Cov}(z_{ij}, z_{ik}) &\approx 4h^2\sigma^2_\varphi\sqrt{f(\mathbf{V}_j)f(\mathbf{V}_k)}, \\
f(\mathbf{V}_j) &= \frac{h^2\mathrm{Tr}(\mathbf{M}_j)^2}{n_j - 1 - (1-h^2)\mathrm{Tr}(\mathbf{M}_j)}.
\end{aligned}$$

Supplemental information

Supplemental information can be found online at https://doi.org/10.1016/j.xhgg.2026.100657.

Received: February 5, 2026
Accepted: July 21, 2026

References

1. Zeggini, E., and Ioannidis, J.P. (2009). Meta-analysis in genome-wide association studies. Pharmacogenomics *10*, 191–201. https://doi.org/10.2217/14622416.10.2.191.
2. Thompson, J.R., Attia, J., and Minelli, C. (2011). The meta-analysis of genome-wide association studies. Briefings Bioinf. *12*, 259–269. https://doi.org/10.1093/bib/bbr020.
3. Yao, Y., and Ochoa, A. (2023). Limitations of principal components in quantitative genetic association models for human studies. eLife *12*, e79238. https://doi.org/10.7554/eLife.79238.
4. Devlin, B., and Roeder, K. (1999). Genomic control for association studies. Biometrics *55*, 997–1004. https://doi.org/10.1111/j.0006-341x.1999.00997.x.
5. Voight, B.F., and Pritchard, J.K. (2005). Confounding from Cryptic Relatedness in Case-Control Association Studies. PLoS Genet. *1*, e32. https://doi.org/10.1371/journal.pgen.0010032.
6. Price, A.L., Patterson, N.J., Plenge, R.M., Weinblatt, M.E., Shadick, N.A., and Reich, D. (2006). Principal components analysis corrects for stratification in genome-wide association studies. Nat. Genet. *38*, 904–909. https://doi.org/10.1038/ng1847.
7. Yu, J., Pressoir, G., Briggs, W.H., Vroh Bi, I., Yamasaki, M., Doebley, J.F., McMullen, M.D., Gaut, B.S., Nielsen, D.M., Holland, J.B., et al. (2006). A unified mixed-model method for association mapping that accounts for multiple levels of relatedness. Nat. Genet. *38*, 203–208. https://doi.org/10.1038/ng1702.
8. Astle, W., and Balding, D.J. (2009). Population Structure and Cryptic Relatedness in Genetic Association Studies. Stat. Sci. *24*, 451–471. https://doi.org/10.1214/09-STS307.
9. Kang, H.M., Sul, J.H., Service, S.K., Zaitlen, N.A., Kong, S.y., Freimer, N.B., Sabatti, C., and Eskin, E. (2010). Variance component model to account for sample structure in genome-wide association studies. Nat. Genet. *42*, 348–354. https://doi.org/10.1038/ng.548.
10. Price, A.L., Zaitlen, N.A., Reich, D., and Patterson, N. (2010). New approaches to population stratification in genome-wide association studies. Nat. Rev. Genet. *11*, 459–463. https://doi.org/10.1038/nrg2813.
11. Zhou, W., Nielsen, J.B., Fritsche, L.G., Dey, R., Gabrielsen, M.E., Wolford, B.N., LeFaive, J., VandeHaar, P., Gagliano, S.A., Gifford, A., et al. (2018). Efficiently controlling for case-control imbalance and sample relatedness in large-scale genetic association studies. Nat. Genet. *50*, 1335–1341. https://doi.org/10.1038/s41588-018-0184-y.
12. Sul, J.H., Martin, L.S., and Eskin, E. (2018). Population structure in genetic studies: Confounding factors and mixed models. PLoS Genet. *14*, e1007309. https://doi.org/10.1371/journal.pgen.1007309.

13. Lee, H., and Han, B. (2022). A theory-based practical solution to correct for sex-differential participation bias. Genome Biol. *23*, 138. https://doi.org/10.1186/s13059-022-02703-0.
14. Hong, M.M., Froelicher, D., Magner, R., Popic, V., Berger, B., and Cho, H. (2024). Secure discovery of genetic relatives across large-scale and distributed genomic data sets. Genome Res. *34*, 1312–1323. https://doi.org/10.1101/gr.279057.124.
15. Zhang, Q.-X., Liu, T., Guo, X., Zhen, J., Yang, M.y., Khederzadeh, S., Zhou, F., Han, X., Zheng, Q., Jia, P., et al. (2024). Searching across-cohort relatives in 54,092 GWAS samples via encrypted genotype regression. PLoS Genet. *20*, e1011037. https://doi.org/10.1371/journal.pgen.1011037.
16. Wang, S., Kim, M., Li, W., Jiang, X., Chen, H., and Harmanci, A. (2022). Privacy-aware estimation of relatedness in admixed populations. Briefings Bioinf. *23*, bbac473. https://doi.org/10.1093/bib/bbac473.
17. Nasirigerdeh, R., Torkzadehmahani, R., Matschinske, J., Frisch, T., List, M., Späth, J., Weiss, S., Völker, U., Pitkänen, E., Heider, D., et al. (2022). sPLINK: a hybrid federated tool as a robust alternative to meta-analysis in genome-wide association studies. Genome Biol. *23*, 32. https://doi.org/10.1186/s13059-021-02562-1.
18. Kolobkov, D., Mishra Sharma, S., Medvedev, A., Lebedev, M., Kosaretskiy, E., and Vakhitov, R. (2024). Efficacy of federated learning on genomic data: a study on the UK Biobank and the 1000 Genomes Project. Front. Big Data *7*, 1266031. https://doi.org/10.3389/fdata.2024.1266031.
19. Cho, H., Froelicher, D., Chen, J., Edupalli, M., Pyrgelis, A., Troncoso-Pastoriza, J.R., Hubaux, J.P., and Berger, B. (2025). Secure and federated genome-wide association studies for biobank-scale datasets. Nat. Genet. *57*, 809–814. https://doi.org/10.1038/s41588-025-02109-1.
20. Kuang, A., Hivert, M.F., Hayes, M.G., Lowe, W.L., Jr., and Scholtens, D.M. (2025). Multi-ancestry genome-wide association analyses: a comparison of meta- and mega-analyses in the Hyperglycemia and Adverse Pregnancy Outcome (HAPO) study. BMC Genom. *26*, 65. https://doi.org/10.1186/s12864-025-11229-1.
21. Dias, J.-A., Chen, T., Xing, H., Wang, X., Rodriguez, A.A., Madduri, R.K., Kraft, P., and Zhang, H. (2025). Evaluating Multi-Ancestry Genome-Wide Association Methods: Statistical Power, Population Structure, and Practical Implications. Am. J. Hum. Genet. *112*, 2493–2508. https://doi.org/10.1101/2025.03.11.25323772.
22. Reich, D.E., and Goldstein, D.B. (2001). Detecting association in a case-control study while correcting for population stratification. Genet. Epidemiol. *20*, 4–16. https://doi.org/10.1002/1098-2272(200101)20:1<4::AID-GEPI2>3.0.CO;2-T.
23. Bacanu, S.-A., Devlin, B., and Roeder, K. (2002). Association studies for quantitative traits in structured populations. Genet. Epidemiol. *22*, 78–93. https://doi.org/10.1002/gepi.1045.
24. Clayton, D.G., Walker, N.M., Smyth, D.J., Pask, R., Cooper, J.D., Maier, L.M., Smink, L.J., Lam, A.C., Ovington, N.R., Stevens, H.E., et al. (2005). Population structure, differential bias and genomic control in a large-scale, case-control association study. Nat. Genet. *37*, 1243–1246. https://doi.org/10.1038/ng1653.
25. Zhang, F., and Deng, H.-W. (2009). Correcting for Cryptic Relatedness in Population-Based Association Studies of Continuous Traits. Hum. Hered. *69*, 28–33. https://doi.org/10.1159/000243151.
26. Epstein, M.P., Allen, A.S., and Satten, G.A. (2007). A Simple and Improved Correction for Population Stratification in Case-Control Studies. Am. J. Hum. Genet. *80*, 921–930. https://doi.org/10.1086/516842.
27. Wawro, N., Bammann, K., and Pigeot, I. (2006). Testing for Association in the Presence of Population Stratification: A Simulation Study Comparing the S-TDT, STRAT and the GC. Biom. J. *48*, 420–434. https://doi.org/10.1002/bimj.200410214.
28. Chang, C.C., Chow, C.C., Tellier, L.C., Vattikuti, S., Purcell, S.M., and Lee, J.J. (2015). Second-generation PLINK: rising to the challenge of larger and richer datasets. GigaScience *4*. https://doi.org/10.1186/s13742-015-0047-8.
29. Willer, C.J., Li, Y., and Abecasis, G.R. (2010). METAL: fast and efficient meta-analysis of genomewide association scans. Bioinformatics *26*, 2190–2191. https://doi.org/10.1093/bioinformatics/btq340.
30. Ochoa, A., and Storey, J.D. (2021). Estimating FST and kinship for arbitrary population structures. PLoS Genet. *17*, e1009241. https://doi.org/10.1371/journal.pgen.1009241.
31. Aitken, A.C. (1936). IV.—On Least Squares and Linear Combination of Observations. Proc. R. Soc. Edinb. *55*, 42–48. https://doi.org/10.1017/S0370164600014346.
32. Nadarajah, S., and Pogány, T.K. (2015). On the distribution of the product of correlated normal random variables. C. R. Math. *354*, 201–204. https://doi.org/10.1016/j.crma.2015.10.019.
33. Gaunt, R.E. (2022). The basic distributional theory for the product of zero mean correlated normal random variables. Stat. Neerl. *76*, 450–470. https://doi.org/10.1111/stan.12267.
34. Balding, D.J., and Nichols, R.A. (1995). A method for quantifying differentiation between populations at multi-allelic loci and its implications for investigating identity and paternity. Genetica *96*, 3–12. https://doi.org/10.1007/BF01441146.
35. Grau, J., Grosse, I., and Keilwagen, J. (2015). PRROC: computing and visualizing precision-recall and receiver operating characteristic curves in R. Bioinformatics *31*, 2595–2597. https://doi.org/10.1093/bioinformatics/btv153.
36. Mitchell, B.D., Kammerer, C.M., Blangero, J., Mahaney, M.C., Rainwater, D.L., Dyke, B., Hixson, J.E., Henkel, R.D., Sharp, R.M., Comuzzie, A.G., et al. (1996). Genetic and environmental contributions to cardiovascular risk factors in Mexican Americans. The San Antonio Family Heart Study. Circulation *94*, 2159–2170. https://doi.org/10.1161/01.cir.94.9.2159.
37. Conomos, M.P., Laurie, C., Stilp, A., Gogarten, S., McHugh, C., Nelson, S., Sofer, T., Fernández-Rhodes, L., Justice, A., Graff, M., et al. (2016). Genetic Diversity and Association Studies in US Hispanic/Latino Populations: Applications in the Hispanic Community Health Study/Study of Latinos. Am. J. Hum. Genet. *98*, 165–184. https://doi.org/10.1016/j.ajhg.2015.12.001.
38. Listgarten, J., Lippert, C., Kadie, C.M., Davidson, R.I., Eskin, E., and Heckerman, D. (2012). Improved linear mixed models for genome-wide association studies. Nat. Methods *9*, 525–526. https://doi.org/10.1038/nmeth.2037.
39. Cheng, R., Parker, C.C., Abney, M., and Palmer, A.A. (2013). Practical Considerations Regarding the Use of Genotype and Pedigree Data to Model Relatedness in the Context of Genome-Wide Association Studies. G3 *3*, 1861–1867. https://doi.org/10.1534/g3.113.007948.

40. Yang, J., Zaitlen, N.A., Goddard, M.E., Visscher, P.M., and Price, A.L. (2014). Advantages and pitfalls in the application of mixed-model association methods. Nat. Genet. *46*, 100–106. https://doi.org/10.1038/ng.2876.
41. Shchur, V., and Nielsen, R. (2018). On the number of siblings and p-th cousins in a large population sample. J. Math. Biol. *77*, 1279–1298. https://doi.org/10.1007/s00285-018-1252-8.
42. Pirastu, N., Cordioli, M., Nandakumar, P., Mignogna, G., Abdellaoui, A., Hollis, B., Kanai, M., Rajagopal, V.M., Parolo, P.D.B., Baya, N., et al. (2021). Genetic analyses identify widespread sex-differential participation bias. Nat. Genet. *53*, 663–671. https://doi.org/10.1038/s41588-021-00846-7.
43. Wood, A.R., Esko, T., Yang, J., Vedantam, S., Pers, T.H., Gustafsson, S., Chu, A.Y., Estrada, K., Luan, J., Kutalik, Z., et al. (2014). Defining the role of common variation in the genomic and biological architecture of adult human height. Nat. Genet. *46*, 1173–1186. https://doi.org/10.1038/ng.3097.
44. Berg, J.J., Harpak, A., Sinnott-Armstrong, N., Joergensen, A.M., Mostafavi, H., Field, Y., Boyle, E.A., Zhang, X., Racimo, F., Pritchard, J.K., and Coop, G. (2019). Reduced signal for polygenic adaptation of height in UK Biobank. eLife *8*, e39725. https://doi.org/10.7554/eLife.39725.
45. Sohail, M., Maier, R.M., Ganna, A., Bloemendal, A., Martin, A.R., Turchin, M.C., Chiang, C.W., Hirschhorn, J., Daly, M.J., Patterson, N., et al. (2019). Polygenic adaptation on height is overestimated due to uncorrected stratification in genome-wide association studies. eLife *8*, e39702. https://doi.org/10.7554/eLife.39702.
46. Loh, P.-R., Kichaev, G., Gazal, S., Schoech, A.P., and Price, A.L. (2018). Mixed-model association for biobank-scale datasets. Nat. Genet. *50*, 906–908. https://doi.org/10.1038/s41588-018-0144-6.
47. Bulik-Sullivan, B.K., Loh, P.R., Finucane, H.K., Ripke, S., Yang, J., Patterson, N., Daly, M.J., Price, A.L., and Neale, B.M. (2015). LD Score regression distinguishes confounding from polygenicity in genome-wide association studies. Nat. Genet. *47*, 291–295. https://doi.org/10.1038/ng.3211.
48. Mugo, J.W., Mulder, N., and Chimusa, E.R. (2025). Data simulation to optimize frameworks for genome-wide association studies in diverse populations. Front. Genet. *16*, 1559496. https://doi.org/10.3389/fgene.2025.1559496.
49. Turley, P., Walters, R.K., Maghzian, O., Okbay, A., Lee, J.J., Fontana, M.A., Nguyen-Viet, T.A., Wedow, R., Zacher, M., Furlotte, N.A., et al. (2018). Multi-trait analysis of genome-wide association summary statistics using MTAG. Nat. Genet. *50*, 229–237. https://doi.org/10.1038/s41588-017-0009-4.
50. Manichaikul, A., Mychaleckyj, J.C., Rich, S.S., Daly, K., Sale, M., and Chen, W.M. (2010). Robust relationship inference in genome-wide association studies. Bioinformatics *26*, 2867–2873. https://doi.org/10.1093/bioinformatics/btq559.
51. Gusev, A., Lowe, J.K., Stoffel, M., Daly, M.J., Altshuler, D., Breslow, J.L., Friedman, J.M., and Pe'er, I. (2009). Whole population, genome-wide mapping of hidden relatedness. Genome Res. *19*, 318–326. https://doi.org/10.1101/gr.081398.108.
52. Dimitromanolakis, A., Paterson, A.D., and Sun, L. (2019). Fast and Accurate Shared Segment Detection and Relatedness Estimation in Un-phased Genetic Data via TRUFFLE. Am. J. Hum. Genet. *105*, 78–88. https://doi.org/10.1016/j.ajhg.2019.05.007.
53. Seidman, D.N., Shenoy, S.A., Kim, M., Babu, R., Woods, I.G., Dyer, T.D., Lehman, D.M., Curran, J.E., Duggirala, R., Blangero, J., and Williams, A.L. (2020). Rapid, Phase-free Detection of Long Identity-by-Descent Segments Enables Effective Relationship Classification. Am. J. Hum. Genet. *106*, 453–466. https://doi.org/10.1016/j.ajhg.2020.02.012.
54. Yang, J., Weedon, M.N., Purcell, S., Lettre, G., Estrada, K., Willer, C.J., Smith, A.V., Ingelsson, E., O'Connell, J.R., Mangino, M., et al. (2011). Genomic inflation factors under polygenic inheritance. Eur. J. Hum. Genet. *19*, 807–812. https://doi.org/10.1038/ejhg.2011.39.

HGGA, Volume *7*

Supplemental information

Genetic association meta-analysis is susceptible to confounding by between-study cryptic relatedness

Tiffany Tu and Alejandro Ochoa

Supplemental Figures

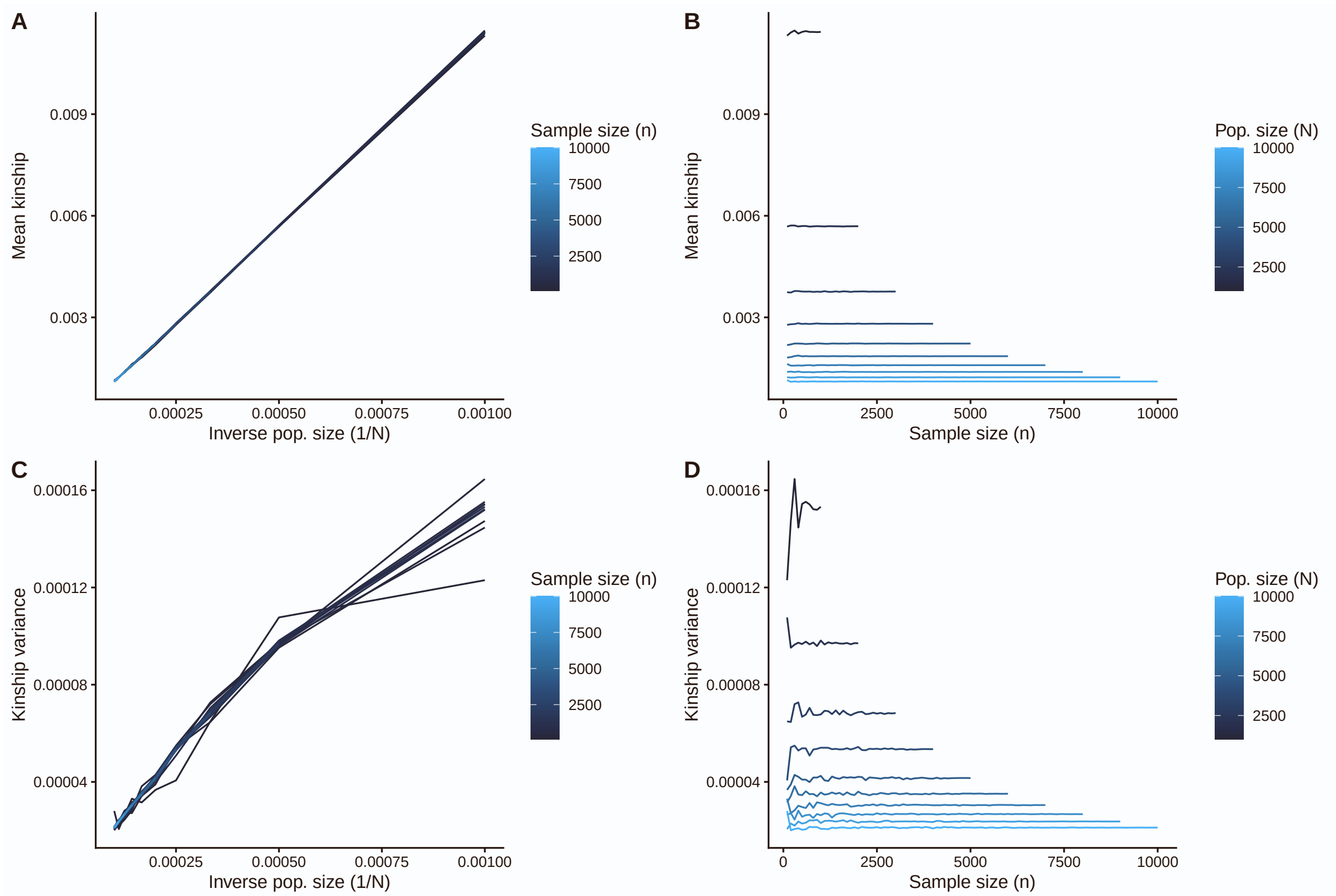

Fig. S1: **Mean and variance of kinship values between studies depend on population size N but not sample size n.** We drew random large population pedigrees, random subsets of them to form samples, and calculated kinship mean and variance between two random partitions of the sample. Sample estimates plotted are averaged over 10 replicates. **A.** Mean kinship is very well predicted by the inverse of the population size (sample size slices are shown in color, they all overlap). Our best model $\bar{\varphi} = 11.29/N$ had a practically perfect fit ($R^2 = 0.9999$). **B.** Mean kinship does not depend on sample size given the population size (note every population size slice is approximately constant). **C.** Kinship variance is again well predicted by the inverse of the population size, with more noise at the smaller sample size slices. Our best model $\sigma^2_\varphi \approx 0.1125/N + 0.001419/\sqrt{N}$ was not visually as good fit ($R^2 = 0.9958$ on the curve averaged over sample sizes weighted by sample size) compared to our model for the mean. **D.** Kinship variance also does not depend on sample size given the population size, but are again noisier for smaller n.

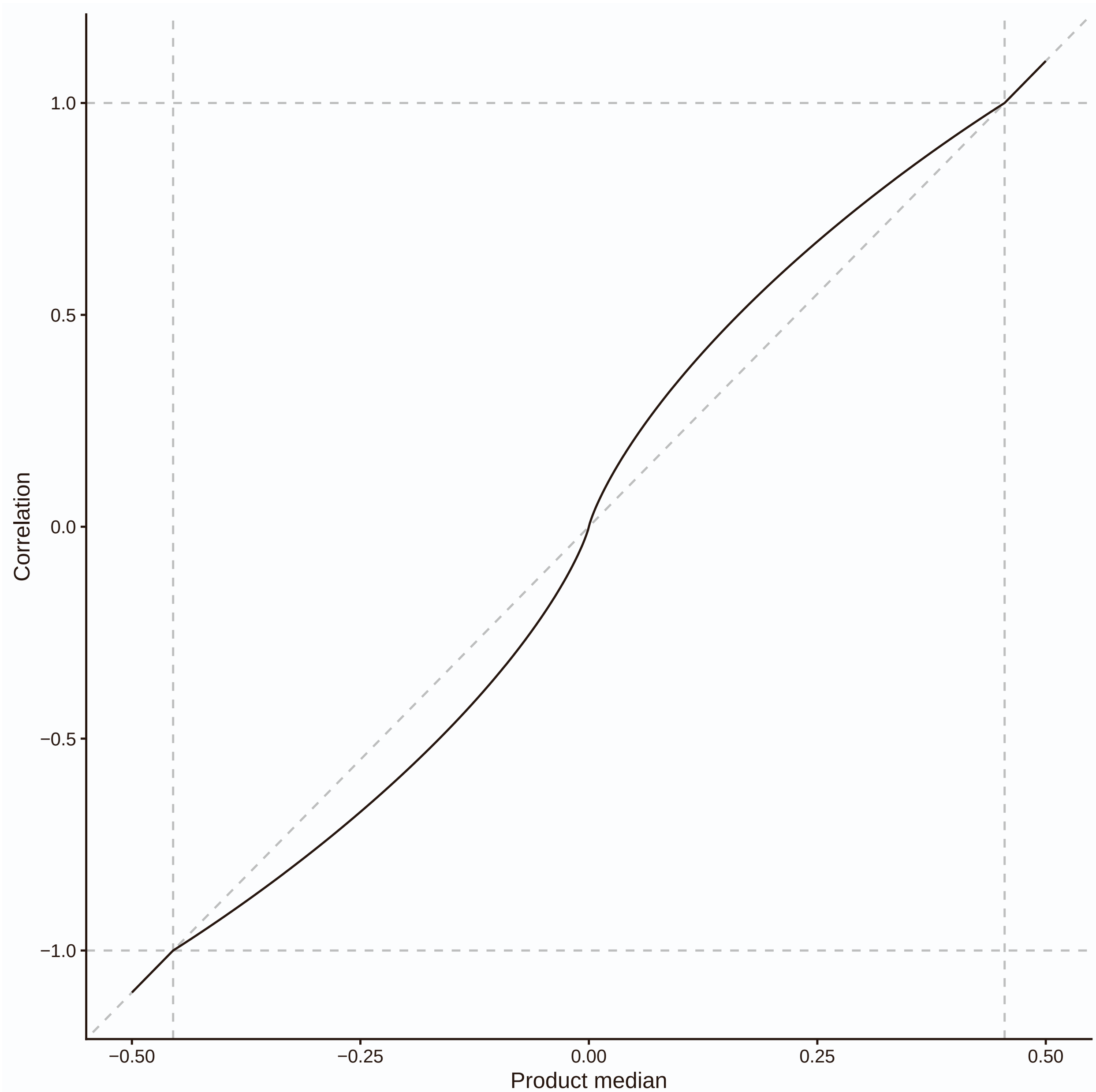

Fig. S2: **Function mapping correlated z-score product medians to correlation parameters.** The vertical gray lines mark $x = -q$ and $x = q$, where $q \approx 0.455$ is the median χ^2_1. The horizontal gray lines mark $y = -1$ and $y = 1$. The diagonal line corresponds to $y = x/q$, which is the standard inflation factor when the two z-scores in the product are equal. The curve and the diagonal line cross at the origin. Thus, for $0 < x < q$, the naive estimate x/q underestimates the true correlation ρ.

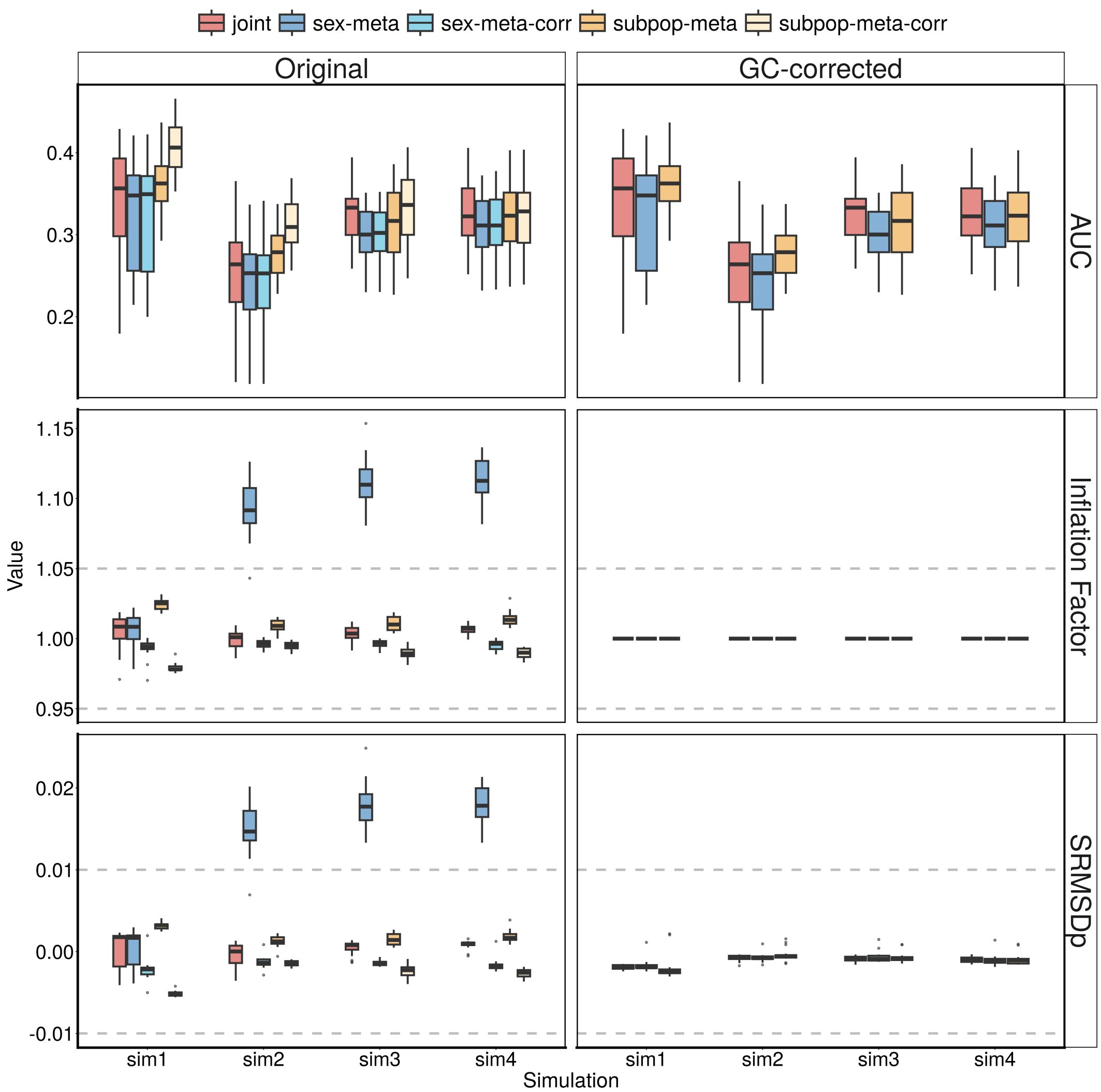

Fig. S3: **Simulation results for binary traits.** Results from 20 replicates of binary traits exhibit similar patterns as for quantitative traits (Figure 4) across all four simulation scenarios.

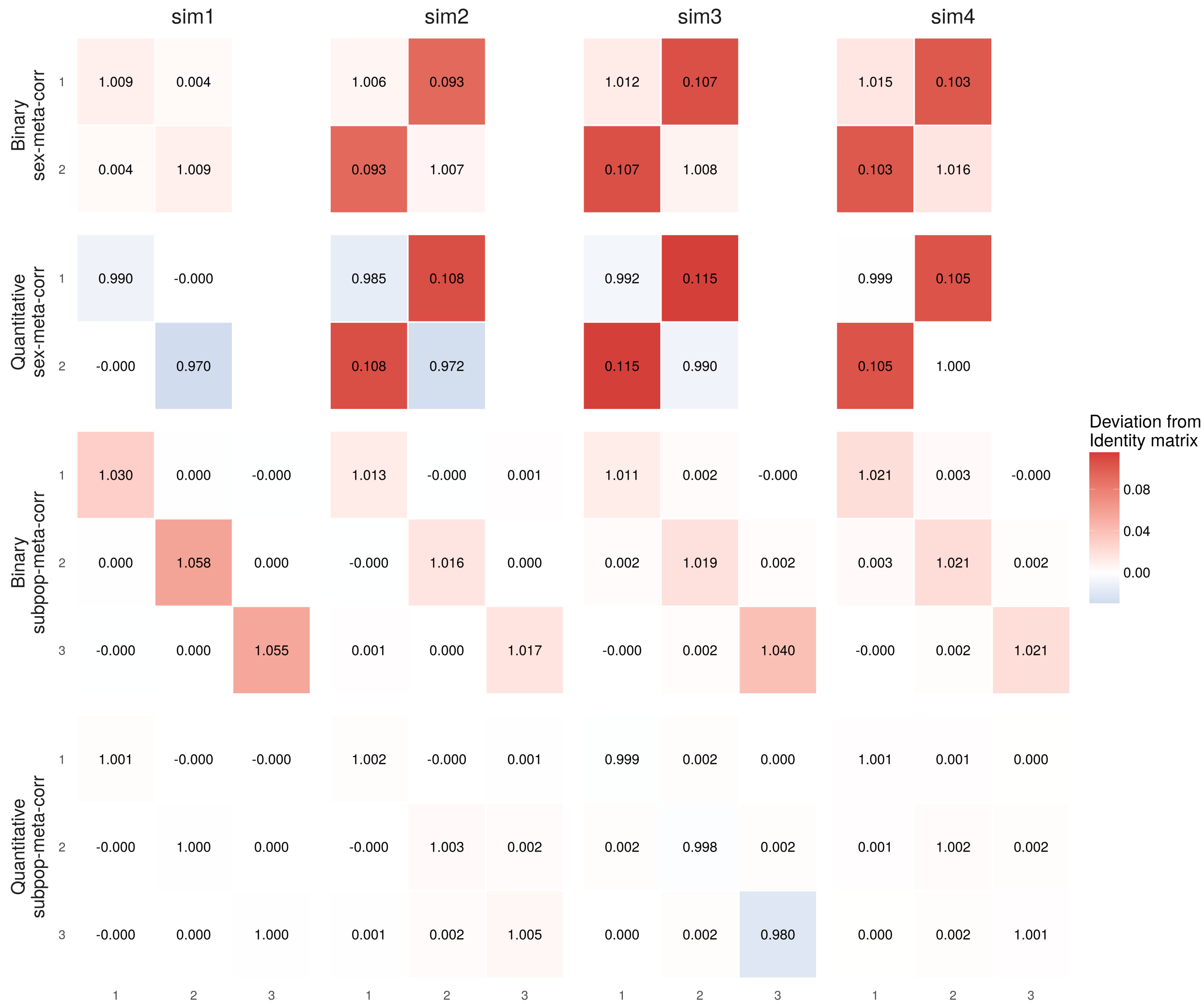

Fig. S4: **Estimated correlation matrices R from meta-corr.** Each cell displays in the number the raw $\mathbf{R}$ value, and in color the value of $\mathbf{R} - \mathbf{I}$, with white corresponds to no deviation from the standard meta-analysis assumption of $\mathbf{R} = \mathbf{I}$ (cases where studies are independent for off-diagonal values, calibrated for diagonal). Deviations reveal where our method provides a correction. All values are averages over the 20 replicates. Correlation matrices are estimated using the median approach.

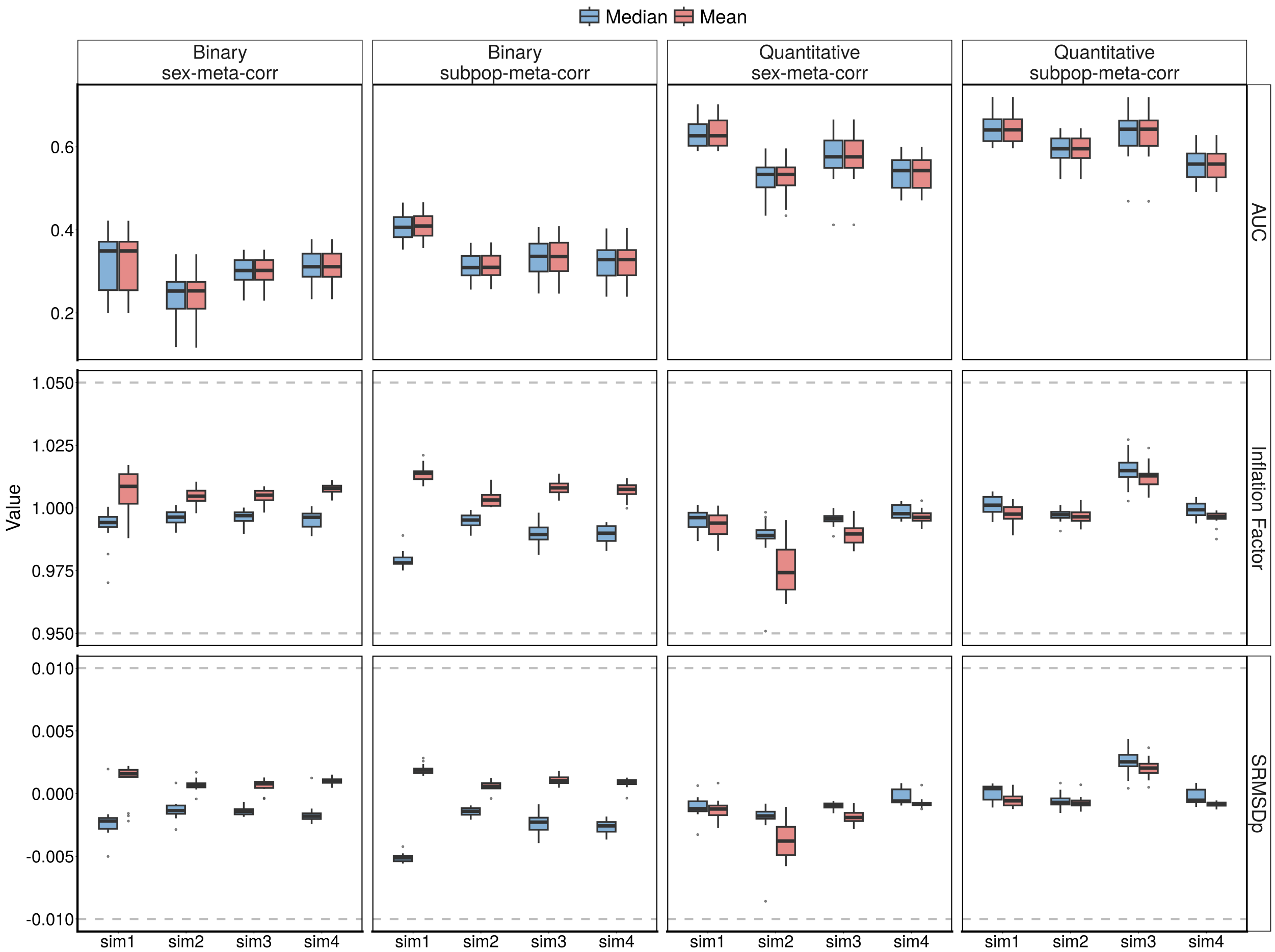

Fig. S5: **Median-based estimation of the covariance matrix performs marginally better than the mean-based approach with 100 causal variants.** We compare median and mean estimators of the covariance in our proposed meta-analysis method for both sex-stratified and subpopulation-stratified meta-analyses. AUC_{PR}, $SRMSD_p$ and inflation are comparable between the two estimators across trait types. Note that all values are well within the thresholds for calibration. For binary traits, however, the mean-based approach produced relatively higher inflation factors.

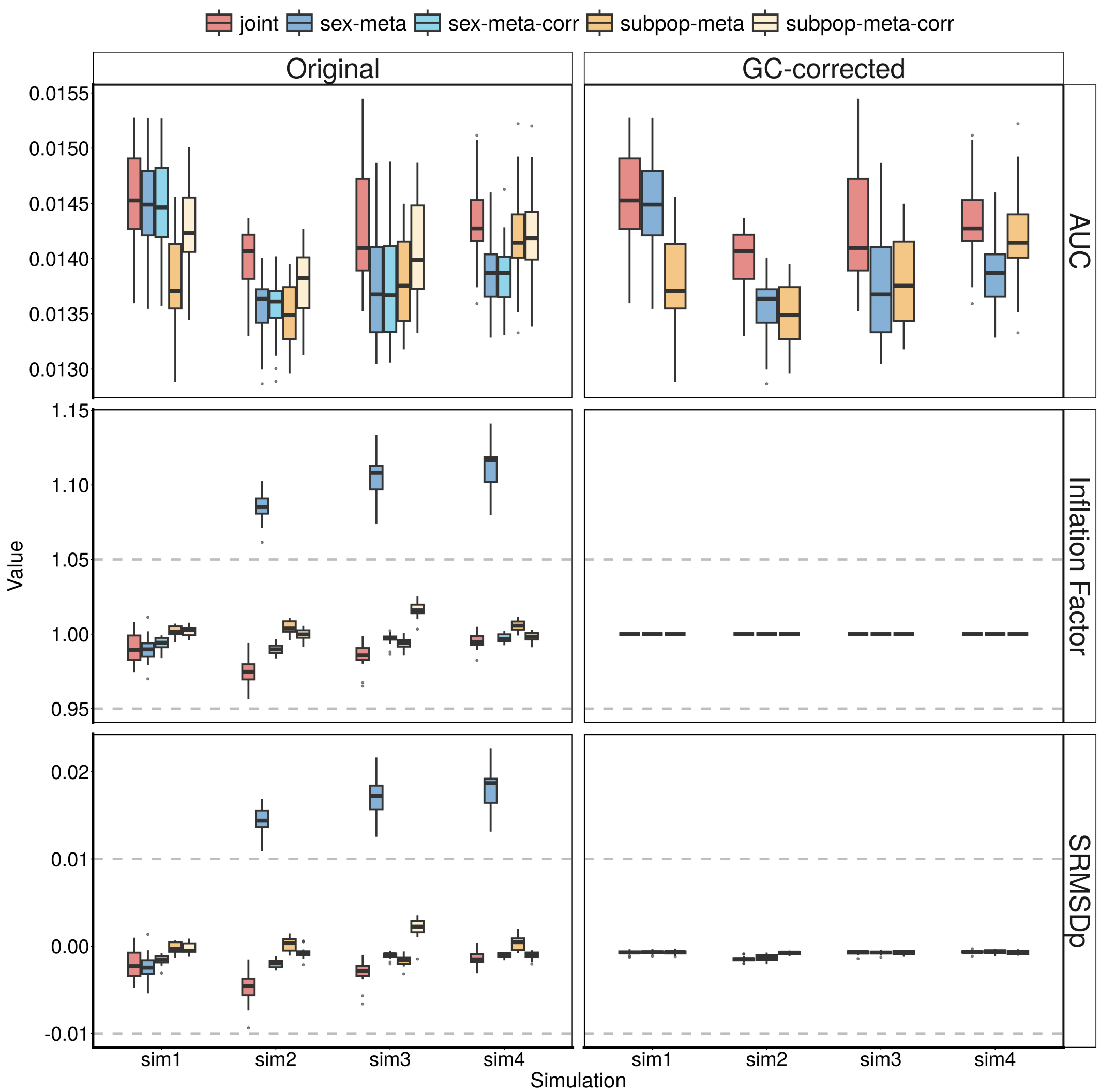

Fig. S6: **Simulation results for quantitative traits with 5,000 causal variants.** Across all four simulations, results from 20 replicates of 5,000 causal variants exhibit similar pattern as the main quantitative analysis (with 100 causal variants; Figure 4). Only trait is re-simulated, keeping genotypes, allele frequencies, and population structure unchanged. AUC_{PR} values are substantially lower partly due to the absense of LD in our simulation framework.

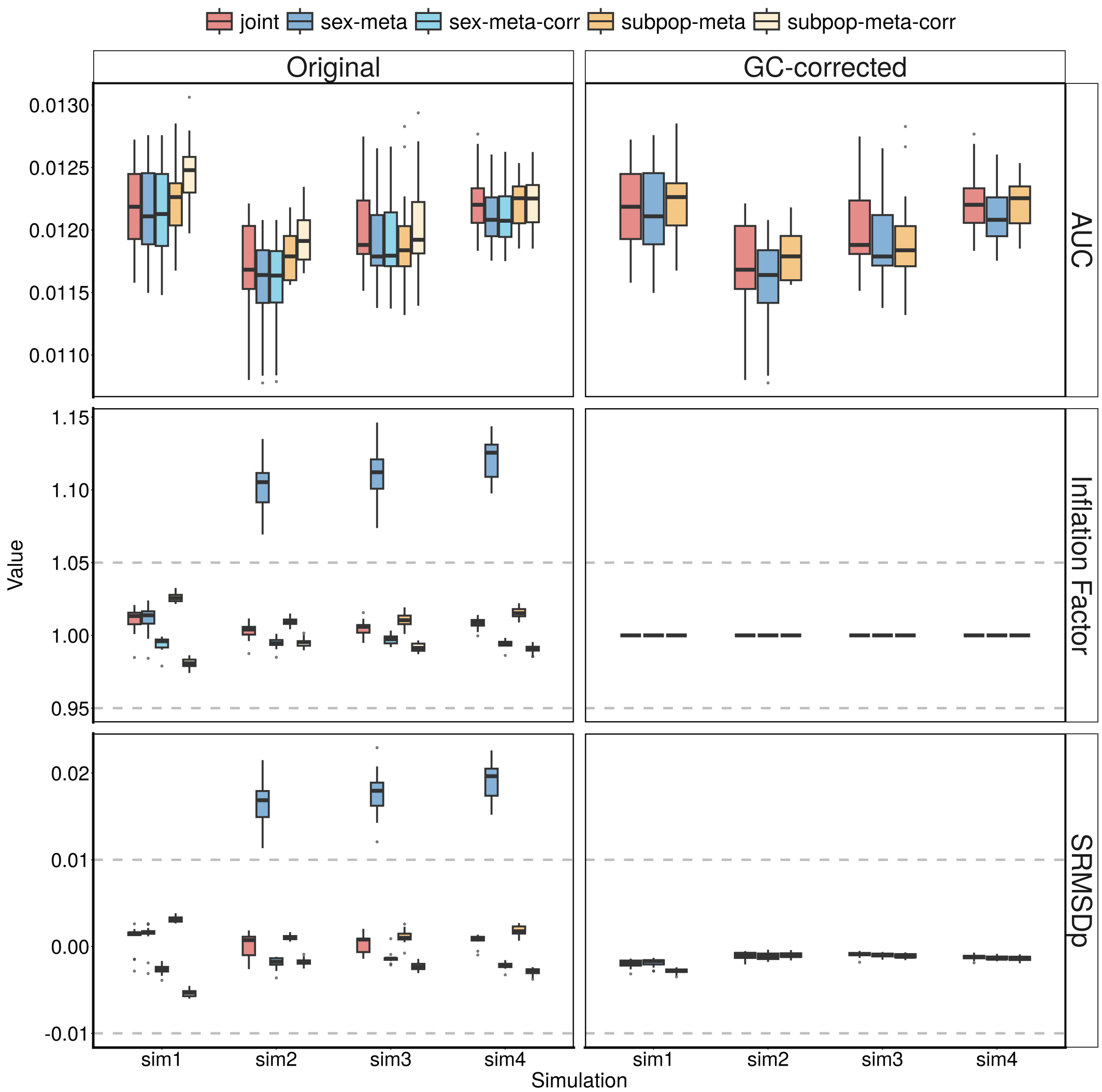

Fig. S7: **Simulation results for binary traits with 5,000 causal variants.** Across all four simulations, results from 20 replicates of 5,000 causal variants exhibit similar pattern as the main binary analysis (with 100 causal variants; Figure S3). Similar to Figure S6, AUC_{PR} values are substantially lower than in the main analysis.

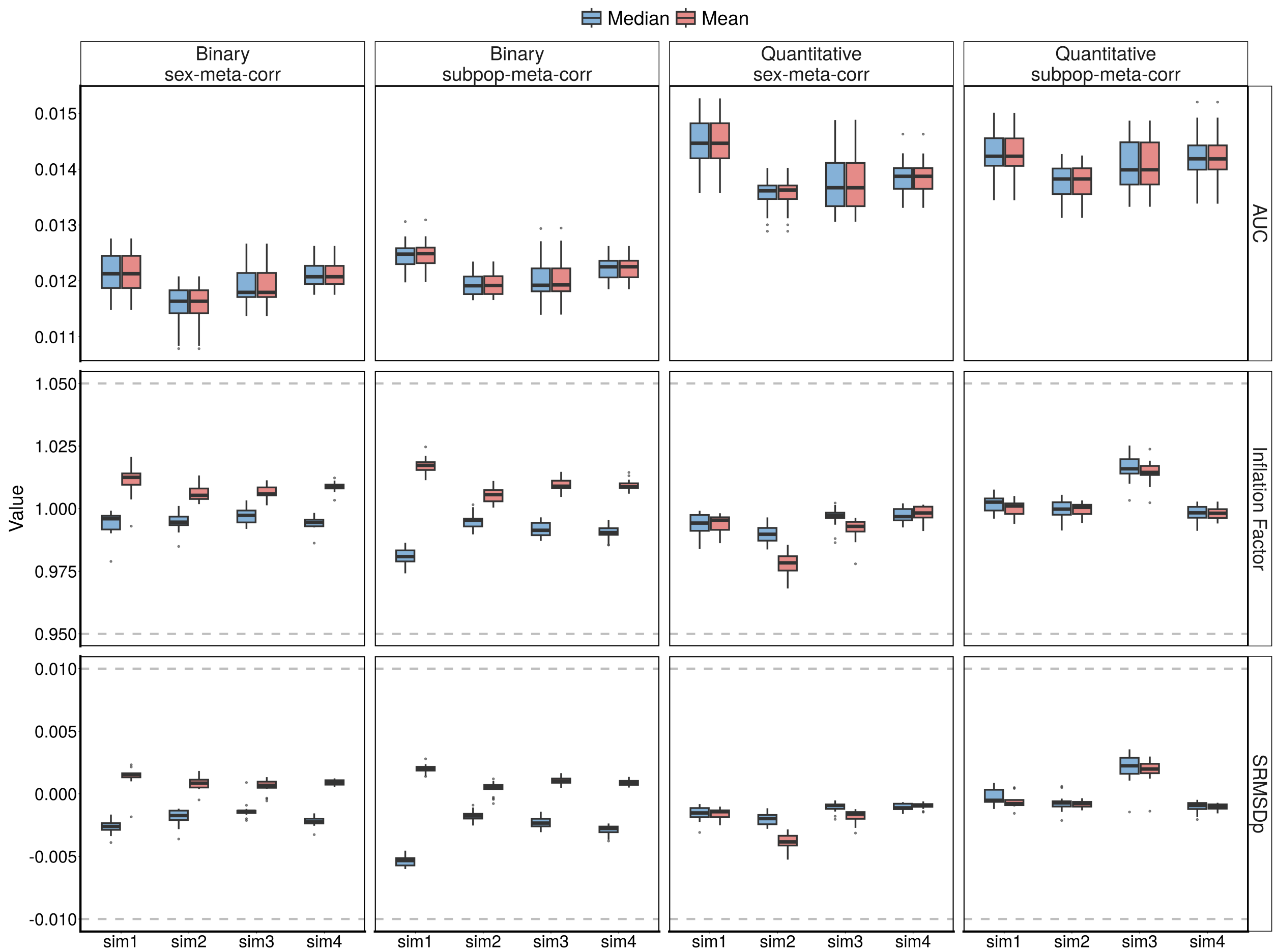

Fig. S8: **Under a more polygenic model of 5,000 causal variants, median-based covariance estimation maintains its marginal advantage over mean-based approach.** We repeat the median and mean estimator comparisons from Figure S5 after re-simulating traits with 50 times more causal SNPs. Inflation factors and $SRMSD_p$ remain well calibrated within thresholds for both estimators, and the mean-based approach again shows higher inflation factors for binary traits. AUC_{PR} values collapse to a much narrower range.

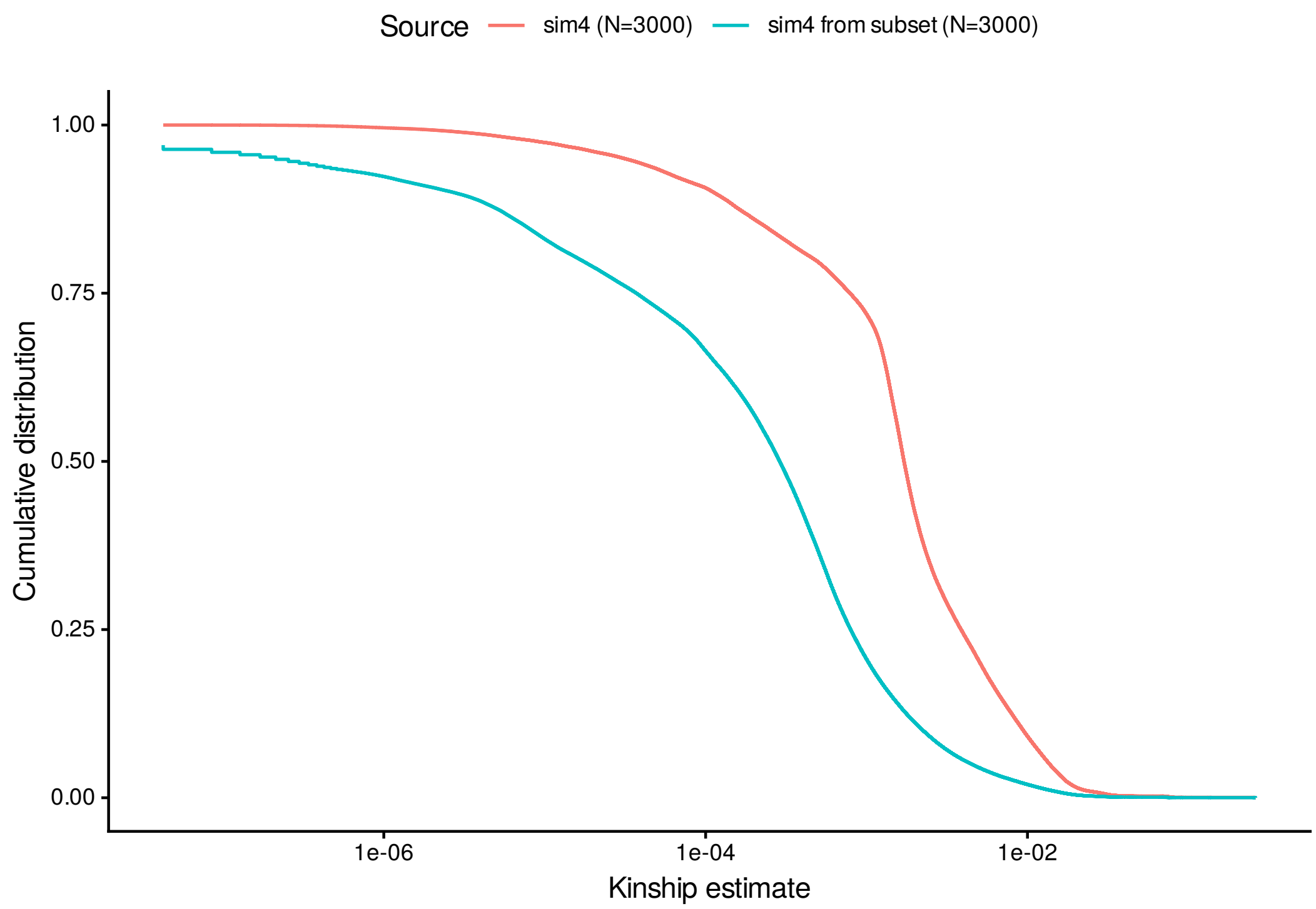

Fig. S9: **Cumulative distribution of pairwise kinship estimates at $n = 3,000$ under different starting population sizes.** True kinship values from each simulation were used. Directly simulating 3,000 individuals over 30 generations (sim4) gives us higher pairwise kinship overall, whereas simulating $n = 10,000$ first and then subsetting to $n = 3,000$ produces proportionally fewer large kinship values (for example, many fewer sibling and first cousin pairs). A typical fourth-degree relatedness cutoff value is 2.2e-02. One replicate from each simulation is plotted. Note x-axis is on a log-scale.

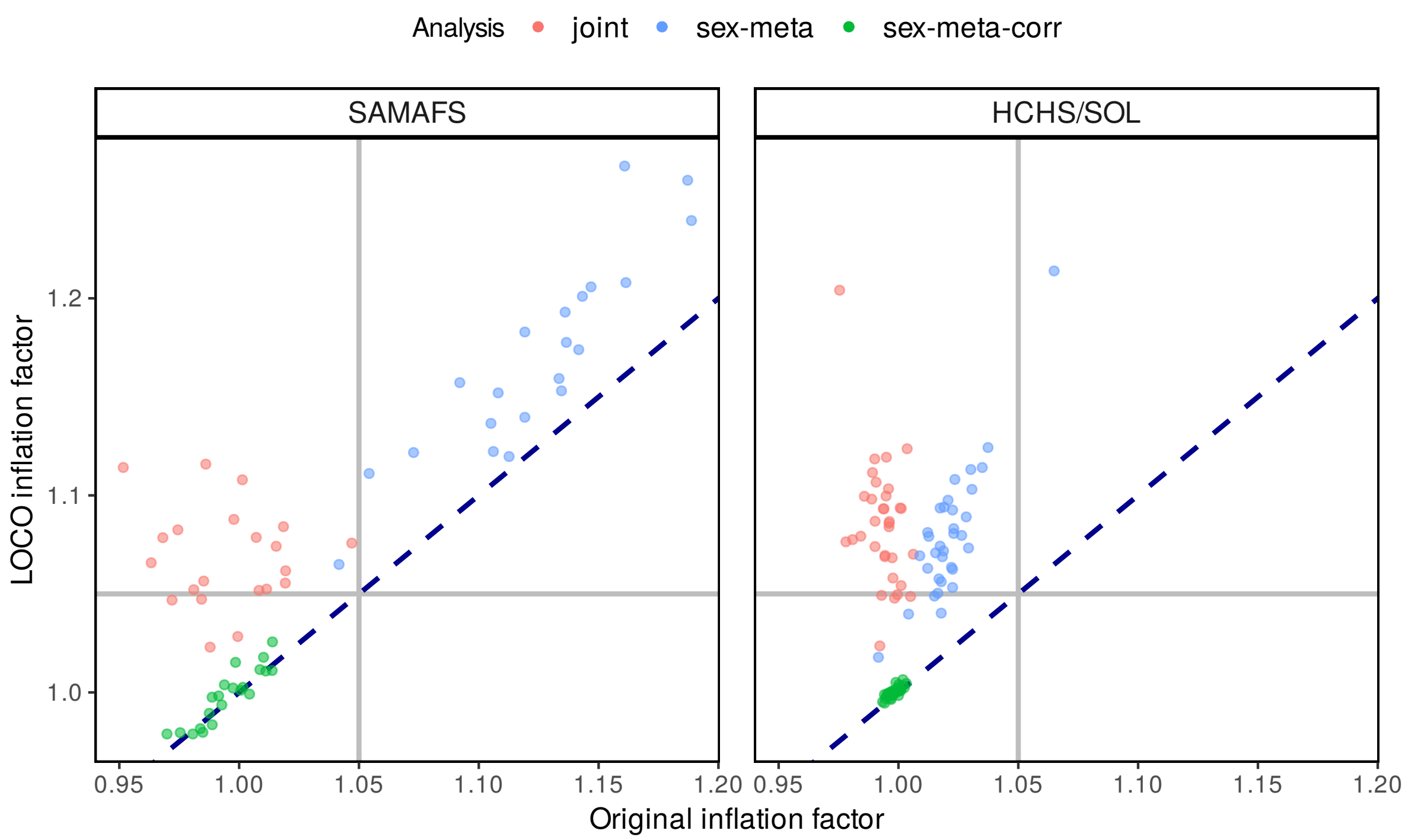

Fig. S10: **LOCO increases inflation factors, but corrected sex-stratified meta-analysis recovers well-calibrated test statistics.** Every trait in both SAMAFS and HCHS/SOL shows greater genomic inflation under LOCO compared to the original analysis for joint and sex-meta (all points above the $y = x$ diagonal). This inflation is successfully eliminated in sex-meta-corr, which clusters near $\lambda = 1.0$.

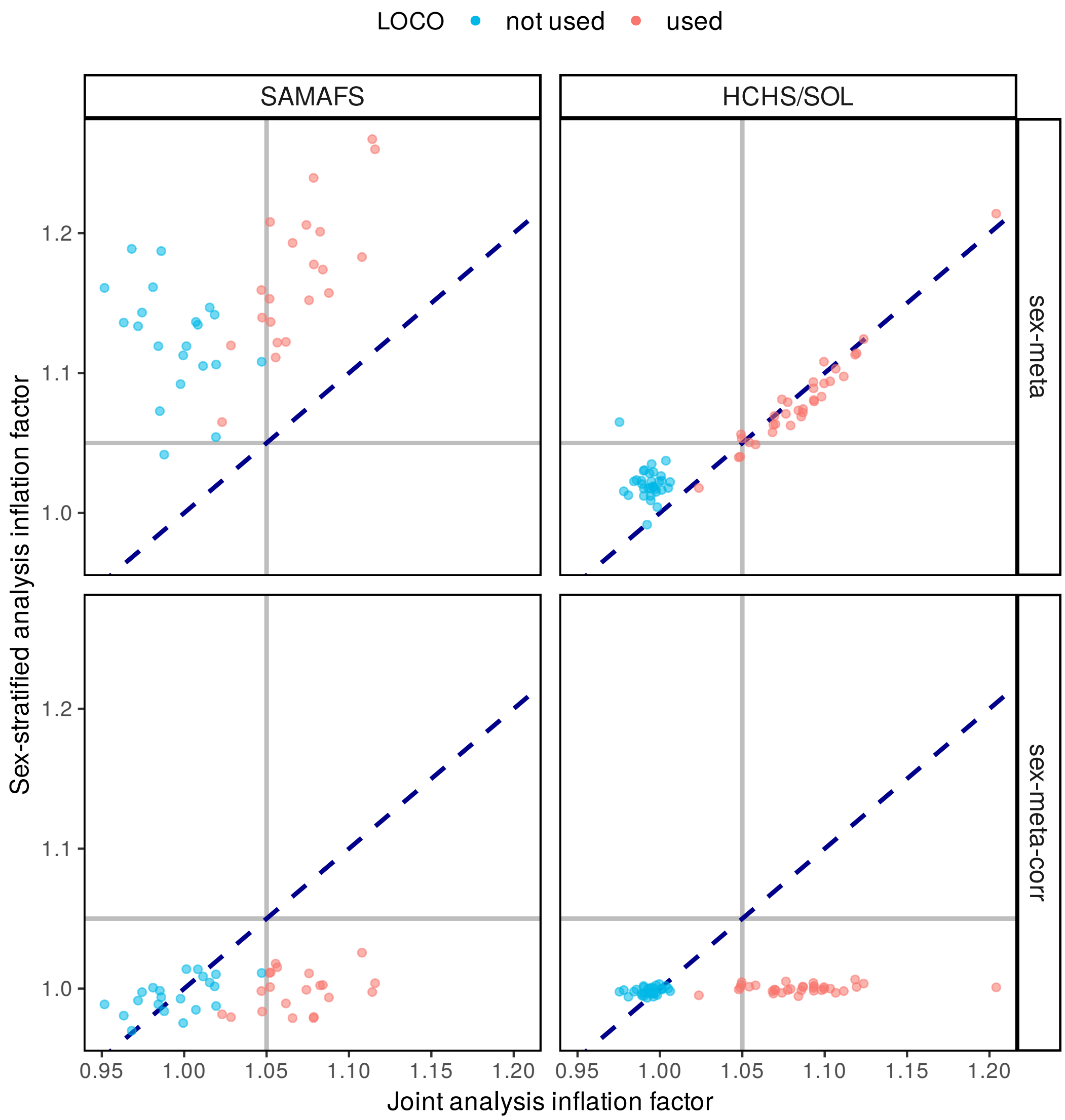

Fig. S11: **Effect of LOCO on inflation factors of joint and sex-stratified meta-analyses.** For joint and sex-meta analyses (top row), inflation factors are greater using LOCO compared to not using it. In SAMAFS, the increases in inflation due to LOCO are such that our main conclusion still holds, namely that meta-analysis has greater inflation compared to the joint analysis (pink points above the $y = x$ diagonal). However, in HCHS/SOL the conclusion no longer holds when using LOCO (pink points are around or below the diagonal). In contrast, sex-meta-corr (bottom row) yields inflation factors close to 1.0, so the correlated meta-analysis method effectively corrects inflated test statistics regardless of use of LOCO.

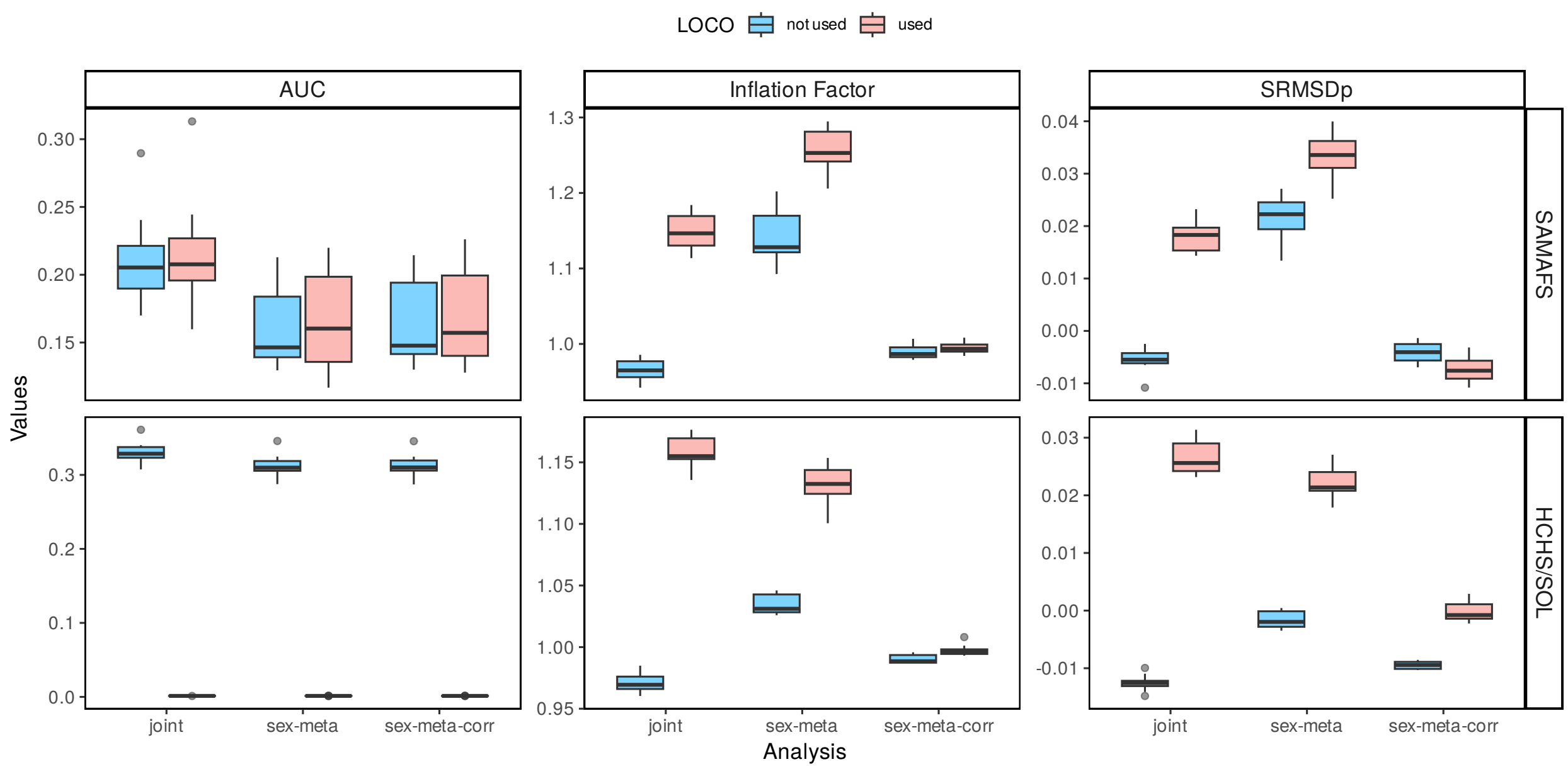

Fig. S12: **Evaluation metrics for LOCO in joint versus sex-stratified meta-analysis on real datasets with simulated traits of 10 replicates.** For both SAMAFS and HCHS/SOL, we observe more favorable metrics when LOCO is not used, with higher AUC_{PR} and lower inflation factors. LOCO inflation is most severe in sex-meta, consistent with our earlier findings. Sex-meta-corr substantially reduces both inflation factor and $SRMSD_p$ relative to sex-meta.

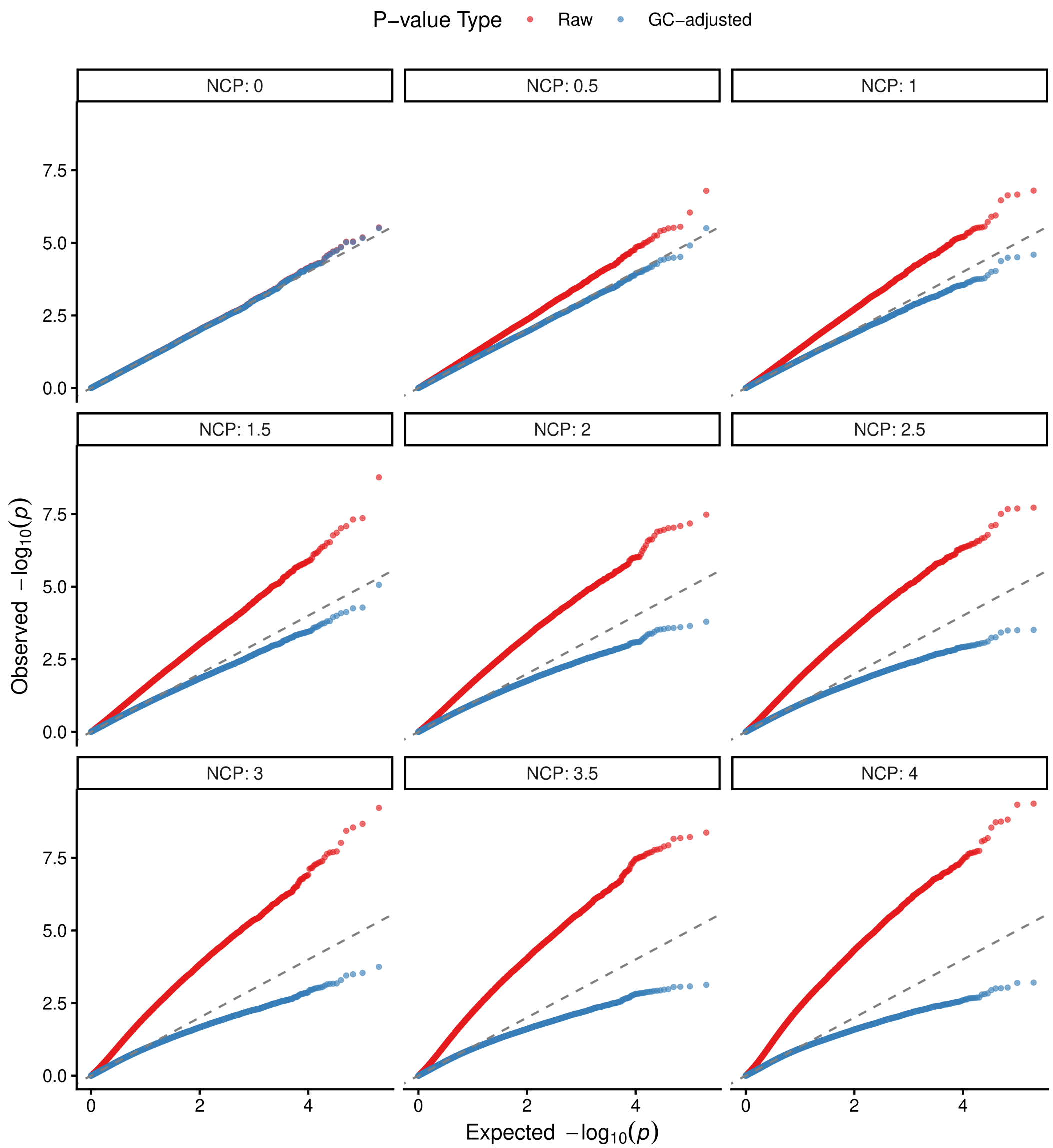

Fig. S13: **Quantile-quantile plots for simulated high polygenicity and high power p-values before and after GC adjustment.** Each panel simulated 200,000 p-values, half from a uniform distribution (null) and half from an alternative distribution, thus mimicking a highly polygenic trait. Alternative test statistics are drawn from $t \sim \chi^2_1(\lambda_{\mathrm{NCP}})$, which is a chi-squared distribution with one degree of freedom and a non-centrality parameter (NCP) λ_{NCP}, then p-values are calculated assuming the null hypothesis $t \sim \chi^2_1(0)$. The NCP increases the mean of the alternative statistics to $1 + \lambda_{\mathrm{NCP}}$ (increasing the resulting power), and is varied by panel: 0 represents a case with no power to detect alternatives, and 4 is very high power. GC overcorrects more strongly when there is higher power, which manifests as a large segments of the curve well below the $y = x$ diagonal (dashed gray line).

Supplemental Tables

Table S1: Traits included in SAMAFS analysis, including data transformation applied.

Trait	Code	Min	Max	Data Transformation
Cystatin C	cystatin_c	0.72	998.45	Log-transformed
2-hour Insulin	a2h_ins	2.4	686.05	Log-transformed
Creatinine	creatinine	35.36	769.08	Log-transformed
Adiponectin	adiponectin	0.48	87.28	Log-transformed
Leptin	leptin	0.01	161.96	Log-transformed
Cholesterol	chol	1.99	9.88	Log-transformed
LDL-cholesterol	ldl	0.51	6.95	Log-transformed
HDL-cholesterol	hdl	0.44	3.21	Log-transformed
Triglycerides	tg	0.33	15.35	Log-transformed
Body mass index	bmi	14.43	69.67	Log-transformed
Hip circumference	hipc	52	230.5	Log-transformed
Waist circumference	wasitc	63.5	223	Log-transformed
Wasit to hip ratio	whr	0.44	1.69	Log-transformed
Diastolic blood pressure	dbp	44	126	Log-transformed
Height	height	121	188.65	None
Fasting glucose	fast_glu	2.35	21.04	Log-transformed
Systolic blood pressure	sbp	92	232.5	Log-transformed
Fasting insulin	fast_ins	1.25	264.21	Log-transformed
2-hour glucose	a2h_glu	2.33	31.91	Log-transformed
Weight	weight	38.33	187.97	Log-transformed
Type 2 diabetes	t2d	0	1	None (binary)

Table S2: Traits included in HCHS/SOL analysis, including data transformation applied.

Trait	Code	Units	Min	Max	Data Transformation
Height	HEIGHT	cm	122	199	None
Body mass index	BMI	kg/m2	14.28	70.35	Log-transformed
Waist to hip ratio	WASIT_HIP	NA	0.52	1.31	Log-transformed
Insulin fasting	INSULIN_FAST	calibrated to mU/L	0.33	726	Log-transformed
Glucose fasting	LABA70	mg/dL	52	642	Log-transformed
Glucose post Oral Glucose Tolerance Test (OGTT)	LABA71	mg/dL	29	389	Log-transformed
Cystatin c.	LABA101	mg/L	0.34	10.1	Log-transformed
High-sensitivity c-reactive protein	LABA91	mg/L	0.11	316.9	Log-transformed
Total cholesterol	LABA66	mg/dL	62	587	Log-transformed
Triglycerides	LABA67	mg/dL	20	6366	Log-transformed
HDL-cholesterol	LABA68	mg/dL	9	141	Log-transformed
LDL-cholesterol	LABA69	mg/dL	22	417	Log-transformed
Weight	ANTA4	kg	34.5	189.2	Log-transformed
Waist girth	ANTA10A	cm	31	202	Log-transformed
Hip girth	ANTA10B	cm	35	306	Log-transformed
Average systolic	SBPA5	mmHg	74	232	Log-transformed
Average diastolic	SBPA6	mmHg	39	135	Log-transformed
Insulin post OGTT	INSULIN_OGTT	calibrated to mU/L	1.5	960.5	Log-transformed
White blood count	LABA1	count × 10e9	1.2	31.8	None
% Neutrophils	LABA10	percentage	0	92	None
% Lymphocytes	LABA11	percentage	5	90	None
% Monocytes	LABA12	percentage	0	47	None
% Eosinophils	LABA13	percentage	0	36	None
% Basophils	LABA14	percentage	0	5.5	None
Red blood cell count	LABA2	count × 10e12	2.21	7.61	Log-transformed
Hemoglobin	LABA3	g/dL	4.3	20.6	Log-transformed
Platelet count	LABA9	count × 10e9	26	874	Log-transformed
Alanine Aminotransferase	LABA74	U/L	5	651	Log-transformed
Aspartate Aminotransferase	LABA75	U/L	5	515	Log-transformed
Gamma-Glutamyl Transferase (GGT)	LABA102	U/L	2	1660	Log-transformed
Ferritin	LABA103	ug/L	7	12745	Log-transformed
Iron	LABA82	ug/dL	8	320	Log-transformed
Apnea/Hypopnea Index	SLPA54	events/hour	0	141.67	Log-transformed

Table S3: Significance of AUC_{PR} overperformance of subpop-meta-corr versus joint.

Num causal	Simulation	Trait type	P-value[a]
100	sim1	Quant	5.08e-04 *
100	sim2	Quant	9.54e-07 *
100	sim3	Quant	6.04e-03
100	sim4	Quant	1.00
100	sim1	Binary	2.38e-05 *
100	sim2	Binary	1.61e-04 *
100	sim3	Binary	0.194
100	sim4	Binary	0.536
5,000	sim1	Quant	1.00
5,000	sim2	Quant	1.00
5,000	sim3	Quant	1.00
5,000	sim4	Quant	1.00
5,000	sim1	Binary	2.93e-04 *
5,000	sim2	Binary	2.93e-04 *
5,000	sim3	Binary	0.478
5,000	sim4	Binary	0.449

[a]All are paired one-sided Wilcoxon tests with 20 replicates. P=1 indicates that joint performed better than subpop-meta-corr.

*Significant at 0.05 level after Bonferroni correction for 16 tests (threshold of 3.13e-03).

Supplemental Acknowledgments

We would like to thank the staff and participants of the SAMAFS and HCHS/SOL study for their important contributions. The research reported in this article was supported by National Institutes of Health grants. The genetic and phenotypic data were provided by the San Antonio Family Heart Study (SAFHS) investigators and supported by the National Heart, Lung, and Blood Institute (NHLBI) [P01 HL045222] and the San Antonio Family Diabetes/Gallbladder Study (SAFDGS) investigators and supported by the National Institute of Diabetes and Digestive and Kidney Diseases (NIDDK) [R01 DK047482, R01 DK053889]. The phenotypic data were also provided by the Veterans Administration Genetic Epidemiology Study (VAGES) investigators and supported by the Health Services Research and Development, U.S., Department of Veteran Affairs and the Family Investigation of Nephropathy and Diabetes (FIND) - San Antonio (FIND-SA) Component and its extension called the Extended FIND (E-FIND) [U01 DK57295] investigators and supported by the NIDDK. The SAFHS gene expression assays were supported by a donation from the Azar and Shepperd families. The exome sequencing, exome chip genotypic, and whole genome sequencing data were provided by the T2D-GENES Consortium grants U01 DK085524, U01 DK085584, U01 DK085501, U01 DK085526, and U01 DK085545 and investigators supported by the NIDDK. This manuscript was not prepared in collaboration with investigators of the T2D-GENES SAMAFS/Consortium and does not necessarily reflect the opinions or views of the members of the T2D-GENES SAMAFS/Consortium, or the NIDDK. The Hispanic Community Health Study/Study of Latinos is supported by contracts from the National Heart, Lung, and Blood Institute (NHLBI) to the University of North Carolina at Chapel Hill, Chapel Hill, NC (N01-HC65233), University of Miami, Miami, FL (N01-HC65234), Albert Einstein College of Medicine, Bronx NY (N01-HC65235), University of Illinois, Chicago IL (N01-HC65236), and San Diego State University, San Diego CA (N01-HC65237). The following Institutes/Centers/Offices contribute to the HCHS/SOL through a transfer of funds to the NHLBI: National Center on Minority Health and Health Disparities, the National Institute of Deafness and Other Communications Disorders, the National Institute of Dental and Craniofacial Research, the National Institute of Diabetes and Digestive and Kidney Diseases, The National Institute of Neurological Disorders and Stroke, and the Office of Dietary Supplements.
